# Improving the usefulness of US mortality data: new methods for reclassification of underlying cause of death

**DOI:** 10.1186/s12963-016-0082-4

**Published:** 2016-04-28

**Authors:** Kyle J. Foreman, Mohsen Naghavi, Majid Ezzati

**Affiliations:** Department of Epidemiology and Biostatistics, Imperial College London, 10 Elephant Lane, SE16-4JD London, UK; Institute for Health Metrics and Evaluation, University of Washington, Seattle, USA

**Keywords:** Vital registration data, Cause of death certification, Garbage codes, Statistical cause of death models

## Abstract

**Background:**

Mortality data are affected by miscertification of the medical cause of death deaths and changes to cause of death classification systems. We present both mappings of ICD9 and ICD10 to a unified list of causes, and a new statistical model for reducing the impact of misclassification of cause of death.

**Methods:**

We propose a Bayesian mixed-effects multinomial logistic model that can be run on individual record level death certificates to reclassify “garbage-coded” deaths onto causes that are more meaningful for public health purposes. The model uses information on the contributing causes of death and demographic characteristics of each decedent to make informed predictions of the underlying cause of death. We apply our method to death certificate data in the US from 1979 to 2011, creating more directly comparable series of cause-specific mortality for 25 major causes of death.

**Results:**

We find that many death certificates coded to garbage codes contain other information that provides strong clues about the valid underlying cause of death. In particular, a plausible underlying cause often appears in the contributing causes of death, implying that it may be incorrect ordering of the causal chain and not missed cause assignment that leads to many garbage-coded deaths. We present an example that redistributes 48 % of heart failure deaths to other cardiovascular diseases, 25 % to ischemic heart disease, and 15 % to chronic respiratory diseases.

**Conclusions:**

Our methods take advantage of more detailed micro-level data than is typically considered in garbage code redistribution algorithms, making it a useful tool in circumstances in which detailed death certificate data needs to be aggregated for public health purposes. We find that this method gives different redistribution results than commonly used methods that only consider population-level proportions.

**Electronic supplementary material:**

The online version of this article (doi:10.1186/s12963-016-0082-4) contains supplementary material, which is available to authorized users.

## Background

Information on mortality from different diseases is an important input to public health decision-making. However, even in countries with vital registration systems and medical certification of deaths, there are difficulties in assessing the levels and trends in cause-specific death rates for two reasons.

First, there are difficulties with cause of death assignment even within a well-defined system like the International Classification of Diseases (ICD). While the ICD has rules for determining a single underlying cause of death in each case (as opposed to possibly multiple contributing, intermediate, or immediate causes of death), it is a complicated rubric that can be disrupted by local coding practices, the particulars of a case, or physician experience [10, 13]. In some countries, software—like the Automated Classification of Medical Entry, ACME, in the United States—attempts to catch and correct some common errors in choosing the underlying cause of death by examining the entire causal chain listed on each death certificate, but it only covers several common issues [3, 8, 12]. Among these, deaths are often attributed to causes that should not be considered causes of death either because they are impossible or do not provide useful public health information, often termed “garbage codes.” For instance, ICD10 contains many codes that are useful in classifying morbidity but are not themselves causes of mortality, such as those within Chapter 18 “Symptoms, Signs and Abnormal Clinical and Laboratory Findings, Not Elsewhere Classified.” Other codes, such as heart failure or septicemia, describe intermediate causes of death that most likely have a different underlying cause that would be a better target for public health intervention [16]. As far back as 1948, heart disease classification has been described as a “convenient statistical ‘wastepaper basket’” [22].

Second, the ICD system is currently on its tenth revision, introduced in 1992 [24], and an 11^th^ revision is expected in 2017 [25]. Each revision brings with it new and more specific causes of death, expanding from under 200 to nearly 15,000 codes [11]. While various “bridge coding” exercises attempt to correct for classification changes [6, 18], they often rely on comparability ratios that do not preserve things like total mortality over time or work with small areas data.

We have confronted these problems in our attempts to model over time cause-specific mortality by US county, age, and sex. Drawing on previous research, we have developed mappings of ICD9 and ICD10 to a mutually exclusive and collectively exhaustive set of 25 causes of death that are of public health importance in the US and other high-income countries. These causes provide a relatively detailed view of the cause composition of mortality while avoiding small numbers issues in modeling. We also have developed a new method for correcting garbage codes which takes into account the entire death certificate and is generalizable to multiple types of garbage codes.

## Methods

### Data sources

We used individual level vital registration data, obtained from the US National Center for Health Statistics (NCHS). These data include records for every registered death in the US, with information on decedents’ age, sex, race, state of residence, and cause of death certified and coded according to the ICD system (ICD9 for 1979 through 1998 and ICD10 for 1999 through 2011) [23, 24]. Data on population by age, sex, and state were obtained from the US Census Bureau prior to 1990 and from the NCHS for subsequent years [7].

### Cause list

One of the challenges of using vital registration data for analyzing trends in cause-specific mortality is that each death is assigned a single underlying cause, using an automated algorithm in the ACME software package. In ICD10 at the most detailed level there are thousands of different 5-digit codes to which a death can be assigned, far more than might be used for public health applications. The ICD provides a way to condense diseases into “chapters,” but these aggregations are occasionally too broad for public health purposes (e.g. “cancers”) and are not comparable across ICD revisions [2, 9]. Thus, it is necessary to create a cause of death classification by creating a “map” from ICD codes to a condensed list of mutually exclusive and collectively exhaustive causes of death. The goals when designing such a classification scheme include capturing important causes of death within the country, distinguishing between causes that suggest different public health and health system interventions, minimizing small numbers issues by not preserving too much detail, and clustering together diseases that are epidemiologically related and have relatively similar patterns in time, age, or geography. In order to identify consistent groups of causes that balance between detailed causes and public health utility, we have developed a mapping of each three- or four-digit ICD9 and ICD10 code to a list of 25 collectively exhaustive and mutually exclusive causes of death (Table 1). Detailed mappings for each ICD code can be found in Additional file 1.

**Table 1:** Table of the mutually exclusive, collectively exhaustive causes of death chosen for analysis in the US, not including garbage codes

A Communicable diseases	B Non-communicable diseases	C Injuries
A.1 HIV/AIDS & Tuberculosis	B.1 Cancers	C.1 Unintentional injuries
A.2 Respiratory infections	B.1.1 Lung cancer	C.1.1 Road traffic injuries
A.3 Maternal	B.1.2 Liver cancer	C.1.2 Other unintentional injuries
A.4 Perinatal	B.1.3 Breast cancer	C.2 Intentional injuries
A.5 Intestinal infections	B.1.4 Digestive cancers	C.2.1 Suicide
A.6 Other communicable diseases	B.1.5 Lymphomas & leukemias	C.2.2 Homicide/War
	B.1.6 Other cancers	
	B.2 Diabetes	
	B.3 Cardiovascular Diseases	
	B.3.1 Ischaemic Heart Disease	
	B.3.2 Stroke	
	B.3.3 Other Cardiovascular Diseases	
	B.4 Chronic Respiratory Diseases	
	B.5 Cirrhosis	
	B.6 Renal Failure	
	B.7 Other Non-communicable Diseases	
	B.8 Mental and Neurological	

This mapping is based on the Global Burden of Disease 2010 cause of death hierarchy [16], abbreviated to account for small numbers issues when analyzing data at the state level, to minimize the effects of the shift from ICD9 to ICD10, and to reflect the causes of interest in the US across all demographic groups. For example, causes like malaria and schistosomiasis are important for global estimates but are no longer relevant to the US; thus, we combine such deaths with many others under the aggregate category of Other Communicable Diseases. On the other hand, causes of death like diabetes and renal failure are major contributors to US mortality and should be analyzed separately.

### Garbage codes

We classify garbage code deaths into nine different categories depending on the information presented by the underlying cause of death: heart failure (2.3 % of ICD10 deaths in the US), cancers of ill-defined site (1.2 %), septicemia (1.4 %), volume depletion or fluid and electrolyte imbalance (0.3 %), ill-defined cardiovascular disease (2.2 %), injuries of undetermined intent (0.2 %), ill-defined injuries (0.1 %, only found in ICD9), ill-defined infectious diseases (<0.1 %), and ill-defined or unknown cause of death (2.2 %). In total, 9.9 % of deaths in the ICD10 era are assigned and coded to these garbage codes in the US. The proportion ranges from a low of 5.3 % in 15 to 19 year olds, up to 12.5 % in 85 years and older and 14 % in children under 5. Some, such as cancers of ill-defined site or injuries of undetermined intent, lack specificity but still contain some information about the underlying cause of death by indicating what family of causes they likely belong to [4]. Others, such as heart failure or septicemia, only describe the immediate cause of death without much indication as to the underlying cause [16]. And still others, such as ill-defined or unknown cause of death (e.g. the “R” codes in ICD10), contain no information as to what killed the person.

### Statistical methods

We first identified which of the valid underlying causes of death from our grouping could conceivably be an appropriate underlying cause of death for each garbage code, referred to as its “target” causes (Table 2). For instance, in the case of unknown or ill-defined causes of death, we assumed that any of the valid underlying causes could have potentially caused the death; for ill-defined cancers, we only included cancers; and for heart failure we included non-communicable causes of death, excluding cancers and mental and neurological conditions.

**Table 2:** Garbage codes and their target underlying causes. Possible underlying causes are listed in the left column, and garbage codes are listed along the top row. Check marks represent which underlying causes were chosen as potential targets for a given garbage code

Possible true underlying cause	Garbage code								
	Septicemia	Heart failure	Ill-defined cancer	Volume depletion	Ill-defined	Ill-defined cardiovascular	Ill-defined injury	Undetermined intent	Ill-defined infectious
A.1 HIV and tuberculosis	✓			✓	✓				✓
A.2 Respiratory infections	✓			✓	✓				✓
A.3 Maternal conditions	✓			✓	✓				✓
A.4 Perinatal conditions	✓			✓	✓				✓
A.5 Other communicable diseases	✓			✓	✓				✓
B.1.1 Lung cancer	✓		✓	✓	✓				
B.1.2 Liver cancer	✓		✓	✓	✓				
B.1.3 Breast cancer	✓		✓	✓	✓				
B.1.4 Digestive cancers	✓		✓	✓	✓				
B.1.5 Lymphomas and leukaemias	✓		✓	✓	✓				
B.1.6 Other cancers	✓		✓	✓	✓				
B.2 Diabetes mellitus	✓	✓		✓	✓	✓			
B.3.1 Ischaemic heart disease	✓	✓		✓	✓	✓			
B.3.2 Stroke	✓	✓		✓	✓	✓			
B.3.3 Other cardiovascular diseases	✓	✓		✓	✓	✓			
B.4 Chronic respiratory diseases	✓	✓		✓	✓	✓			
B.5 Cirrhosis	✓	✓		✓	✓	✓			
B.6 Renal failure	✓	✓		✓	✓	✓			
B.7 Other non-communicable diseases	✓	✓		✓	✓	✓			
B.8 Mental and neurological conditions					✓				
C.1.1 Road traffic injuries	✓			✓	✓		✓		
C.1.2 Other unintentional injuries	✓			✓	✓		✓	✓	
C.2.1 Suicide	✓			✓	✓		✓	✓	
C.2.2 Homicide and war	✓			✓	✓		✓	✓	

We then used a statistical model to reapportion garbage code deaths to target underlying causes by utilizing all the relevant information found on the death certificate as described below. Specifically, when a garbage code was used as the underlying cause of death, our model used the other information on the death certificate to predict the true underlying cause of death. It accomplished this by comparing to death certificates that listed the garbage code as a contributing cause but assigned a valid underlying cause to the death (with the valid cause coming from the list of targets for that garbage code).

We then used the coefficients estimated using this “training” dataset (i.e., those on which the garbage code was listed as a contributing cause, but one of the target causes was listed as the underlying cause) to predict a non-garbage underlying cause of death for those death certificates which have the garbage code listed as their underlying cause. ICD codes listed on line six were excluded from the training and prediction datasets, as they come from Part II of the death certificate, which corresponds to “other significant conditions contributing to death but not resulting in death” [5].

To achieve this, we ran a Bayesian mixed-effects multinomial logistic regression (Equation 1, described below), with the outcome being the assignment of each target cause as the underlying cause of death.

$$ {y}_i\sim Categorical\left(\frac{ \exp \left({\theta}_i\right)}{{\displaystyle {\sum}_{u=1}^U \exp \left({\theta}_i^{\left[u\right]}\right)}}\right) $$

For u = 1:

$$ {\theta}^{\left[u\right]}=0 $$

For u in [2,U]:

$$ {\theta}^{\left[u\right]} = {\alpha}^{\left[u\right]}+\left({\beta}^{\left[u\right]}\times year\right)+\left({\gamma}^{\left[u\right]}\times \mathrm{\mathcal{M}}\right) + {\pi}_{state}^{\left[u\right]}+{\pi}_{place}^{\left[u\right]}+{\pi}_{race}^{\left[u\right]} $$

The mixed-effects multinomial logistic regression predicts the probability that a particular death (*y*_*i*_) was caused by a underlying cause *u*. We treat the first candidate target cause as a reference category in order to ensure identifiability, setting *θ*^[1]^ to 0, and all other target causes are modeled as relative risk ratios (RRRs) representing the probability of each target being the underlying cause compared to the reference category. For each target cause *u* out of *U* possible causes, the model has a fixed intercept (*α*), a fixed effect on year (*β*) that measures the change over time in the likelihood of a death being attributable to the underlying cause, and fixed effects (*γ*) for the presence of each (non-garbage) cause on the death certificate. These are used at the time of prediction to re-distribute those deaths assigned to garbage codes to corresponding targets causes. It also has random effects on the state in which the person lived, the place of death (e.g. in-patient, out-patient or ER, hospice, nursing home or long-term care, home, or other), and the decedent’s race. These random effects take into account variations across space, place of death, and race in the assignment of garbage codes; they change the absolute probability of assigning a death to a specific target cause after accounting for the RRRs of contributing causes listed on the death certificate.

Weakly informative prior distributions were used for each model component (with details presented in Additional file 2: Appendix A). The model was fit using the Bayesian modeling software Stan, utilizing its No U-Turn Sampler algorithm (Stan model code is available in Additional file 3) [20].

The fixed effects on the presence of each cause of death use binary variables for each of the valid underlying causes of death that indicated whether an ICD code corresponding to that category was found anywhere on the first five lines of the death certificate. The model includes these effects for both target causes and other non-garbage causes, meaning that the presence of a cause of death which is not a conceivable target can still provide information on the underlying cause of death. We then exponentiated the fixed effects (*γ*) on causes listed on the death certificate in order to find the RRRs that a death certificate containing that cause should be properly classified to each target underlying cause, adjusted for the other parameters in the model (state, race, place of death, and year).

After fitting the model on the training data, its parameters were used to predict the probability that each garbage-coded death in the test data was actually due to each target cause. That death can then be proportionally attributed to each target cause based on this prediction or be simply reassigned to the target cause with the highest probability. Since we are interested in population level statistics, we reassigned them proportionally.

We ran separate models for ICD9 (1979–1998) and ICD10 (1999–2011), since there are different coding practices and problems between the two revisions. We also run our analysis separately by sex and age group (under 1 years, 1 to 4 years, and 5-year age groups up to 85-plus years of age) since there are some conditions that only affect certain demographics.

## Results

Table 3 shows, as an example of how the model redistributes garbage codes, the RRR for the redistribution of heart failure deaths to other causes in men aged 70–74 years in ICD10 data, with diabetes mellitus used as the reference category. In other words, the RRR describes how much more likely it is, relative to diabetes, for each death to be attributed to a particular underlying cause after adjustment for other factors (state, place of death, race, and year) on the death certificate. Results for every garbage code, sex, and age group are available upon request from the authors.

**Table 3:** Relative risk ratios for possible actual underlying causes of deaths attributed to heart failure in men aged 70 to 74 in ICD10

Relative risk ratio of underlying cause								
Contributing cause	Diabetes	IHD	Stroke	Other CVD	Chron Resp	Cirrhosis	Renal Failure	Other NCD
HIV & Tuberculosis	1.00x	1.14x	0.92x	0.80x	1.19x	1.01x	0.95x	1.05x
Respiratory infections	1.00x	1.45x	4.46x	0.73x	1.60x	2.05x	1.20x	1.82x
Other communicable	1.00x	1.82x	0.49x	1.74x	1.84x	1.07x	0.98x	0.70x
Lung cancer	1.00x	0.96x	0.98x	1.26x	1.24x	1.01x	1.80x	1.10x
Liver cancer	1.00x	0.71x	0.96x	1.92x	1.14x	1.08x	1.09x	0.86x
Breast cancer	1.00x	0.92x	1.01x	1.02x	1.10x	1.02x	1.03x	1.02x
Digestive cancers	1.00x	2.03x	0.86x	1.46x	3.70x	0.65x	0.81x	1.05x
Lymphomas/Leukemias	1.00x	1.93x	≤0.1x	2.27x	≤0.1x	≤0.1x	1.81x	≤0.1x
Other cancers	1.00x	3.95x	≤0.1x	6.96x	4.02x	≤0.1x	2.32x	7.05x
Diabetes mellitus	1.00x	≤0.1x	≤0.1x	≤0.1x	≤0.1x	≤0.1x	≤0.1x	≤0.1x
Ischemic heart disease	1.00x	≥10x	0.21x	≤0.1x	0.37x	0.13x	1.08x	0.67x
Stroke	1.00x	1.43x	≥10x	1.19x	0.51x	≤0.1x	1.65x	0.43x
Other CVD	1.00x	0.71x	0.58x	≥10x	0.64x	0.18x	1.07x	0.85x
Chronic respiratory diseases	1.00x	3.06x	3.72x	2.43x	≥10x	4.20x	3.31x	4.50x
Cirrhosis	1.00x	1.41x	1.63x	1.10x	1.68x	≥10x	0.92x	1.60x
Renal failure	1.00x	0.59x	1.07x	0.81x	0.48x	0.20x	≥10x	0.44x
Other NCDs	1.00x	0.59x	1.42x	1.06x	0.90x	0.75x	0.62x	≥10x
Mental & neurological	1.00x	1.97x	2.97x	1.51x	2.58x	≥10x	0.97x	1.17x
Road traffic injuries	1.00x	≥10x	≤0.1x	≤0.1x	≤0.1x	≥10x	≥10x	≥10x
Other unintentional injuries	1.00x	1.14x	1.17x	0.95x	0.28x	2.38x	0.92x	1.11x

As expected, having one of the target causes of death present on the death certificate is the strongest indicator of underlying cause; e.g. if ischemic heart disease is listed anywhere on a death certificate containing heart failure, it is far and away the most likely underlying cause. The RRRs are more nuanced for causes that are not themselves in the target list and hence likely underlying causes of death. For instance, a death certificate with heart failure as underlying cause, and tuberculosis as a contributory cause, is most likely to be redistributed to chronic respiratory condition (RRR of 1.19), based on the patterns seen in other death certificates for which chronic respiratory conditions are listed as the underlying cause of death and on which both heart failure and tuberculosis appear as contributing causes. Similarly, a death certificate with heart failure listed as an underlying cause, and a respiratory infection listed as a contributing cause, is most commonly attributed to stroke (RRR of 4.46).

**Fig. 1:**
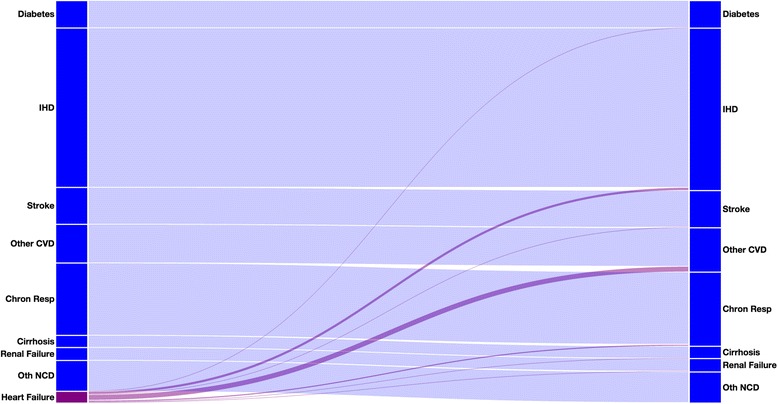
Sankey chart of how heart failure inn men aged 70 to 74 in ICD10 is redistributed. The heights of the left-hand bar represents the proportions of the causes shown prior to redistribution; the right-hand bar shows proportions of causes following redistribution. The connections represent the flow from before to after redistribution, with the purple components representing deaths that were redistributed from heart failure onto other causes

The Sankey chart in Fig. 1 demonstrates the effect of redistributing heart failure deaths in males aged 70 to 74 for ICD10 using our regression results. Even though it is only the third largest target cause of death, other cardiovascular diseases receive more redistributed heart failure deaths (48 %) than either ischemic heart disease (25 %) or chronic respiratory diseases (15 %). This happens because among death certificates with heart failure as the underlying cause of death, and with both ischemic heart disease and other cardiovascular diseases listed, it is most common for the other cardiovascular disease to be chosen as the underlying cause, perhaps because it contains causes like hypertensive heart disease, which are hard to identify themselves but often lead to heart failure as the pathway to death. This is in contrast to a strictly proportional redistribution method, which would redistribute most deaths to ischemic heart disease.

**Fig. 2:**
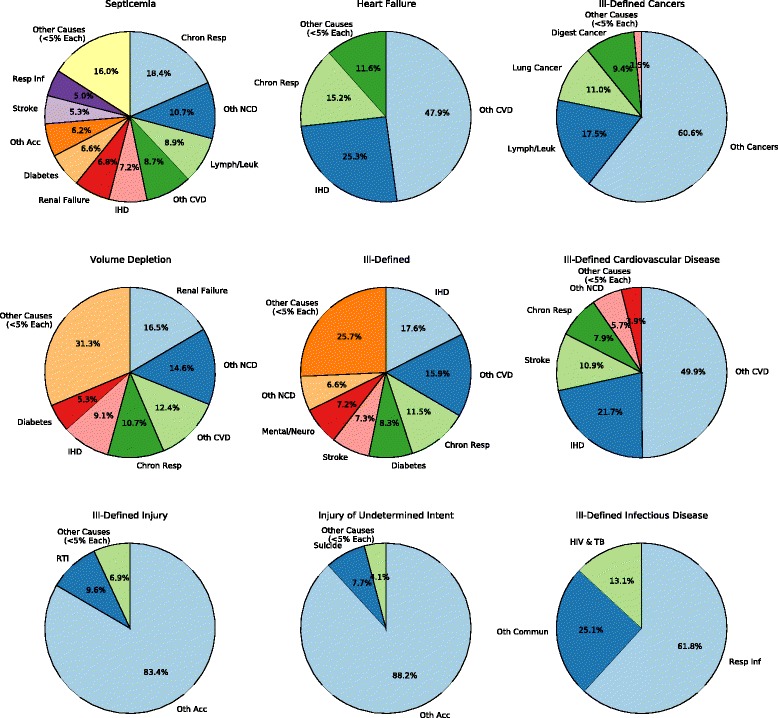
Pie charts showing the redistribution proportions of each of the 9 garbage codes in men aged 70 to 74 in ICD10

Figure 2 shows where deaths from each of the garbage codes present in men ages 70 to 74 in ICD10 are redistributed (charts for each age group, sex, and ICD version can be found in Additional file 4). After redistribution, other cardiovascular diseases gains the most deaths (receiving 26 % of all garbage-coded deaths, mostly coming from heart failure and ill-defined cardiovascular diseases), followed by ischemic heart disease (15 %), other cancers (13 %), and chronic respiratory diseases (10 %).

## Discussion

We have developed a method that uses data on underlying and contributing causes of death to take into account more information when attributing an appropriate underlying cause of death to a death certificate. By incorporating contributing causes as well as demographic data into our model, we are able to tailor our redistribution algorithms more specifically to a target than past methods have done. Our method attempted to create a data-driven algorithm that can generalize to all the types of garbage codes we have described above, including across ICD revisions.

This is in contrast to previous methods for correcting garbage codes, which typically either relied heavily on expert opinion to find reassignment proportions [16] or targeted a single category of garbage codes such as heart failure [1, 21]. Our method shares some similarities with previous studies that have used information from contributing causes not to redistribute garbage codes but to inform reassignment of causes presumed to be overused, such as attributing a portion of diabetes deaths to cardiovascular diseases [14].

Our model also gives different results for heart failure redistribution than previous regression models. For instance, Ahern et al. utilizes the global proportion of heart failure deaths and suggests that 100 % of such cases be redistributed to ischemic heart disease in men aged 50 plus in developed countries [1], compared to reassigning just 25 % of heart failure deaths to ischemic heart disease in our method. Murray et al. use a similar multinomial logistic regression but find that coronary (ischemic) heart disease receives more redistributed heart failure deaths than do other cardiovascular diseases [15]. Similarly, Stevens et al. use coarsened exact matching and redistribute 53 % of heart failure deaths to ischemic heart disease in the US [21].

The difficulty with assessing the “correctness” of any given garbage code redistribution method is that there is no “gold standard” data. Different implementations offer different definitions of what counts as a garbage code, and there are no known datasets with zero garbage; the closest we can come to identifying true underlying clinical cause of death is through autopsy, such as in the 1986 mortality followback study [17, 19]. Because of this lack of concrete data, we are unfortunately left with qualitative instead of quantitative methods for comparing methods.

One qualitative assessment is the extent to which results are driven by expert opinion versus the underlying data. Our method, like all others, uses expert opinion to define what is a garbage code. Informed decision-making also contributes to the assessment of which underlying causes are plausible for a given garbage code, so it is possible that some targets are excluded from redistribution. However, all subsequent steps rely solely on the data to inform regression coefficients and redistribution proportions.

Our method presumes that deaths are assigned to garbage codes either due to incomplete knowledge of the causal pathway, leading physicians to improperly attribute the death to the immediate mode of death (such as heart failure), or because of misapplication of the ICD rules for determining which of the causes in the causal pathway should be categorized as underlying. If, on the other hand, deaths are primarily misattributed to garbage codes due to misdiagnosis, then our algorithm will fail to provide meaningful results. While we assume that in the US health system misdiagnosis is less common than misattribution, absent an autopsy study to validate death certificates against true underlying cause of death we are unable to know definitively whether that is the case.

Additionally, underlying our algorithm is the assumption that the selected garbage codes will also appear on death certificates that have a valid underlying cause of death listed. This is commonly the case for causes like heart failure, renal failure, and others. However, it is much less common to find the ill-defined causes in the “R” chapter of ICD10 on death certificates with valid underlying causes, because these codes are typically only used when little or no information about the cause of death is known. We have applied our algorithm in these cases in order to have a consistent method for producing a mutually exclusive and collectively exhaustive set of cause of death estimates, but custom redistribution algorithms or simply proportional redistribution may be better candidates for correcting ill-defined causes of death.

Reliable methods for constructing comparable and accurate cause-specific mortality time series are necessary for understanding trends in health, which subsequently become inputs to research questions and policy decisions. Many countries have over a century’s worth of vital registration data, but its usefulness is hampered by problems like garbage codes and ICD transitions. As early as the 1940s, researchers have identified “problems of inaccurate diagnosis and improper medical certification,” with heart disease in particular being treated as “a convenient statistical ‘wastepaper basket’” [22]. While improved physician training and better technologies for certifying death have long promised to increase the utility of mortality statistics in the future, we are still left with over a hundred years of data which we can make good use of given better algorithms and statistical methods.

## Additional files

Additional file 1: Mappings of ICD9 and ICD10 codes to condensed set of causes of death, including garbage codes. (XLSX 1139 kb) Additional file 2: Appendix A. (DOCX 101 kb) Additional file 3: Code file used to run the statistical model in the Stan modeling language. (STAN 3 kb) Additional file 4: Pie charts of garbage code redistribution proportions for each age group, sex, and ICD version. (PDF 585 kb)
