# Supplementary material for: Improving the usefulness of US mortality data: new methods for reclassification of underlying cause of death
Source: Popul Health Metr. 2016 Apr 28;14:14. doi: 10.1186/s12963-016-0082-4 (PMC4848792; doi:10.1186/s12963-016-0082-4)
Supplement: Additional file 2: — Appendix A. (DOCX 101 kb) [file 12963_2016_82_MOESM2_ESM.docx]

# Appendix A – Garbage code redistribution model

We fit the following Bayesian mixed effects logistic model for each garbage code *i*, predicting the likelihood of the true underlying cause being cause *u*:

$$y_{i}\sim Categorical\left( \frac{exp(\theta_{i})}{\sum_{u=1}^{U} exp(\theta_{i}^{\left[ u \right]})} \right)$$

Where for *u* = 1:

$$\theta^{\left[ u \right]}=0$$

And for *u* in [2,U]:

$$\theta^{\left[ u \right]}= \alpha^{\left[ u \right]}+\left( \beta^{\left[ u \right]}\times year \right)+\left( \gamma^{\left[ u \right]}\mathcal{\times M} \right)+ \pi_{state}^{\left[ u \right]}+\pi_{place}^{\left[ u \right]}+\pi_{race}^{\left[ u \right]}$$

Where $\alpha$ is a global intercept representing the average likelihood of each cause *u* being the underlying cause, $\beta$ is a slope indicating how the probability of each possible underlying cause changes over time, *year* is the year of death, $\gamma$ is a vector of fixed effects representing how the presence of each cause on the death certificate influences the probability of *u* being the underlying cause, and $\mathcal{M}$ is a design matrix indicating which other causes were present on the death certificate. The $\pi$ terms are random effect vectors on state of residence, place where the death occurred, and the decedent’s race.

We put the following priors on the model parameters. We used weakly informative priors on all effects where possible so that the results are driven by the data. The one exception is that $\alpha$ is centered more tightly around 0 in order to aid in convergence; however, it is constant across causes and thus does not bias the model.

$$\alpha^{\left[ u \right]}\sim Normal\left( 0, 1 \right)$$

$$\beta^{\left[ u \right]}\sim Normal\left( 0, 1 \right)$$

$$\gamma^{\left[ u \right]}\sim Normal(0, 1)$$

$$\pi_{state}^{\left[ u \right]}\sim Normal(0, \sigma_{state}^{[u]})$$

$$\sigma_{state}^{\left[ u \right]}\sim Uniform(0,\infty)$$

$$\pi_{place}^{\left[ u \right]}\sim Normal(0, \sigma_{place}^{[u]})$$

$$\sigma_{place}^{\left[ u \right]}\sim Uniform(0,\infty)$$

$$\pi_{race}^{\left[ u \right]}\sim Normal(0, \sigma_{race}^{[u]})$$

$$\sigma_{race}^{\left[ u \right]}\sim Uniform(0,\infty)$$

We built the model in the Bayesian modeling software Stan and optimize it using Stan’s No U-Turn Sampler method.
