# Supplementary material for: Improving the usefulness of US mortality data: new methods for reclassification of underlying cause of death
Source: Popul Health Metr. 2016 Apr 28;14:14. doi: 10.1186/s12963-016-0082-4 (PMC4848792; doi:10.1186/s12963-016-0082-4)
Supplement: Additional file 4: — Pie charts of garbage code redistribution proportions for each age group, sex, and ICD version. (PDF 585 kb) [file 12963_2016_82_MOESM4_ESM.pdf]

ICD 9
Female, Age 0

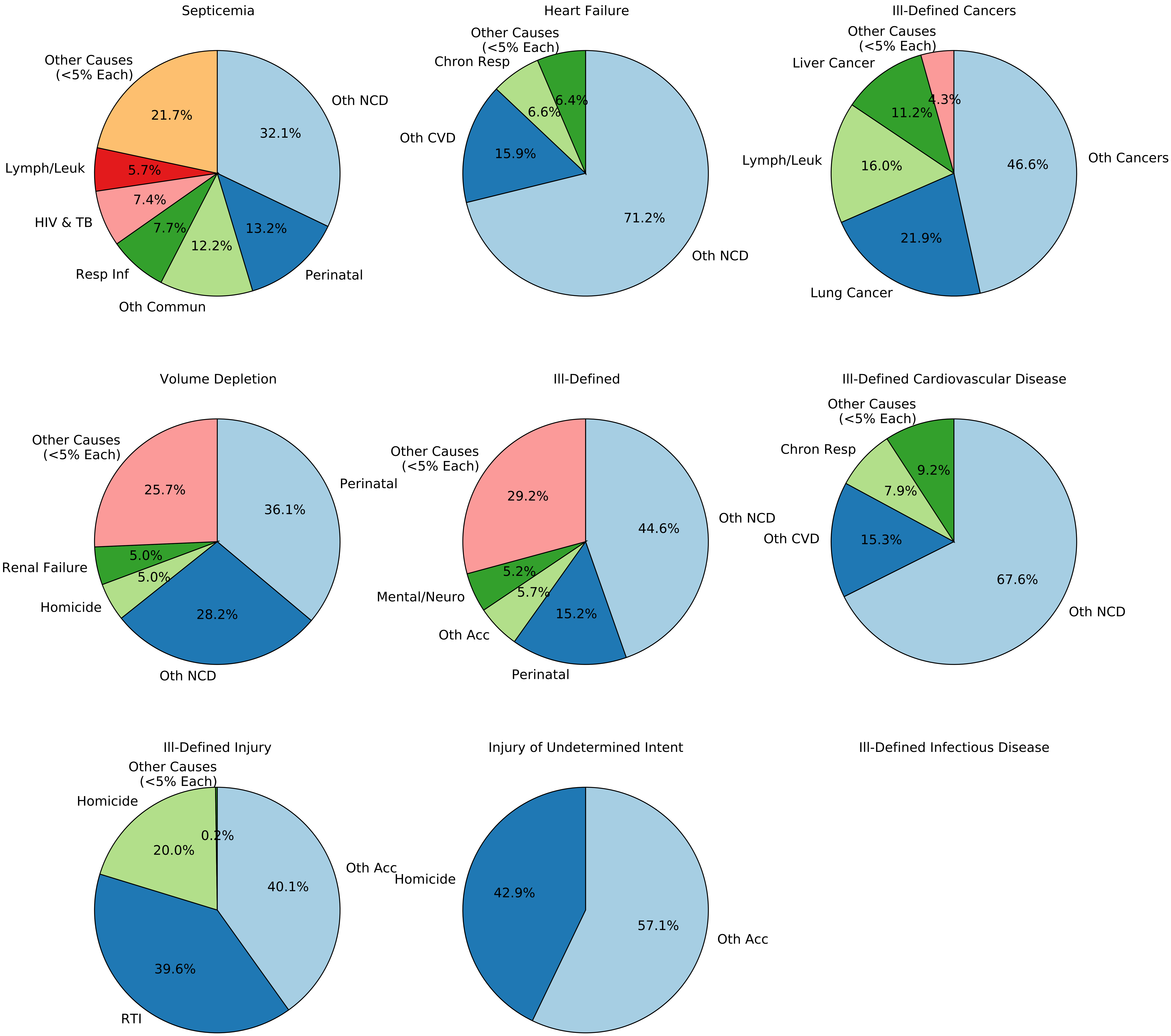

ICD 9
Female, Age 5

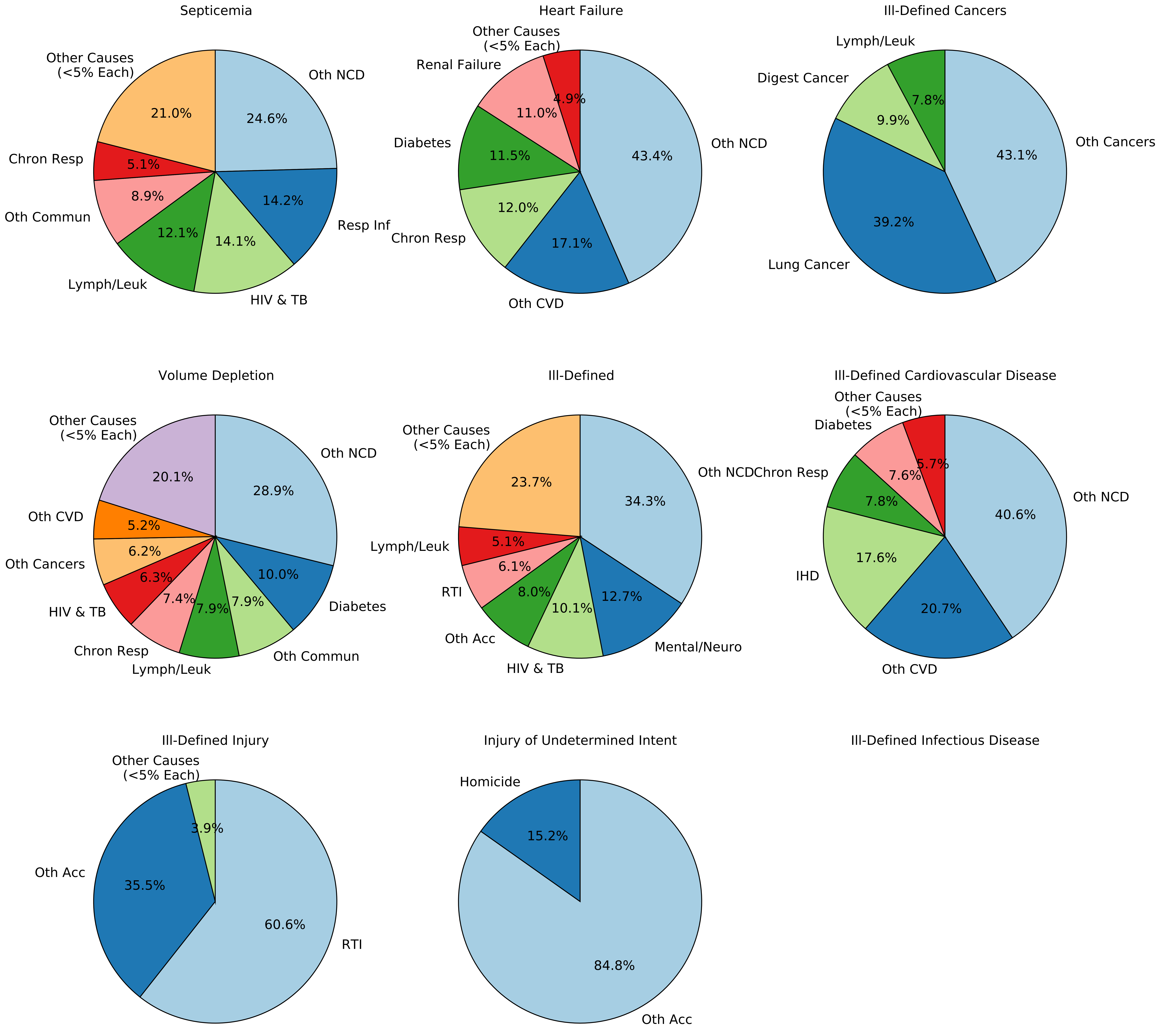

ICD 9
Female, Age 10

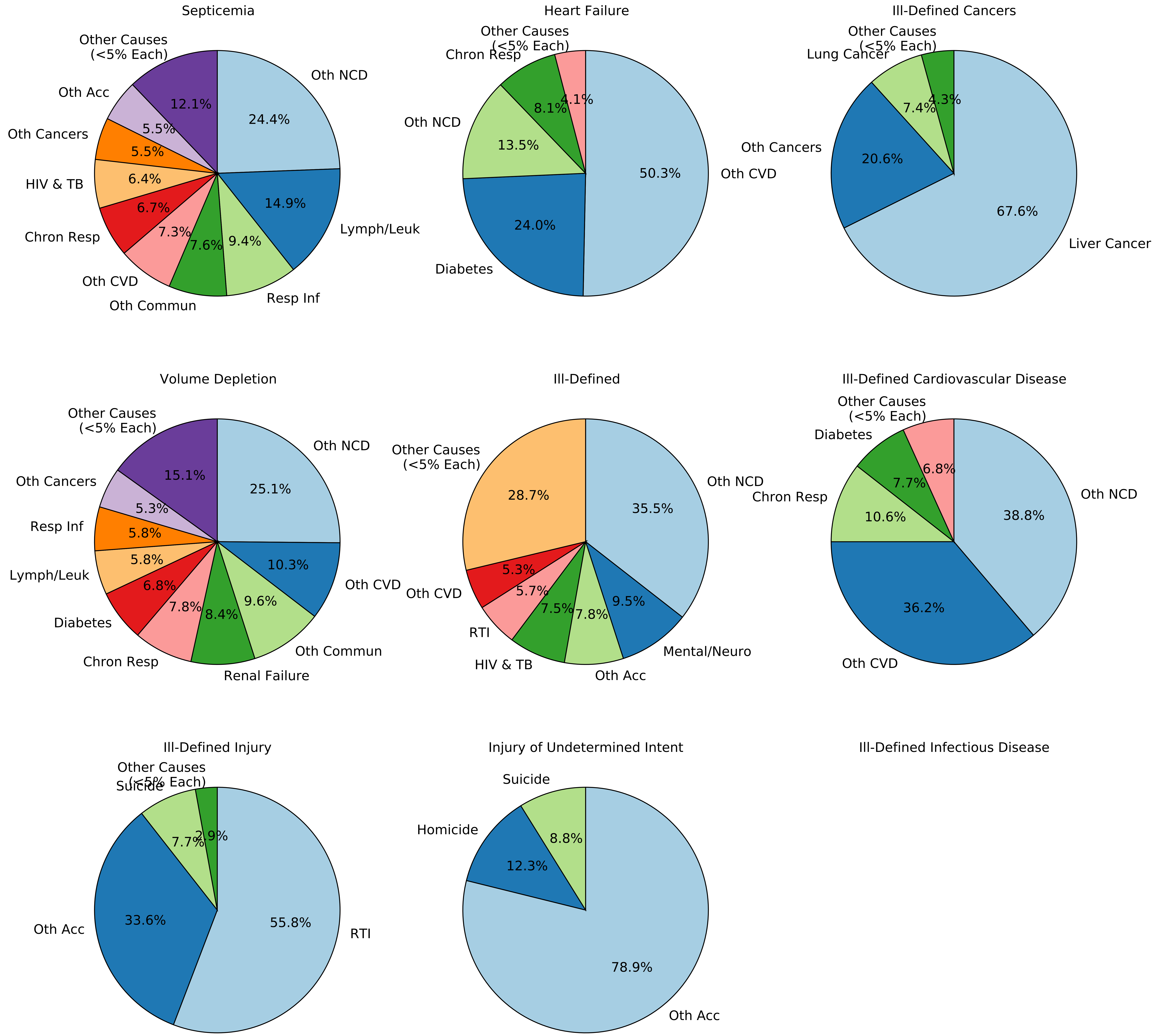

ICD 9
Female, Age 15

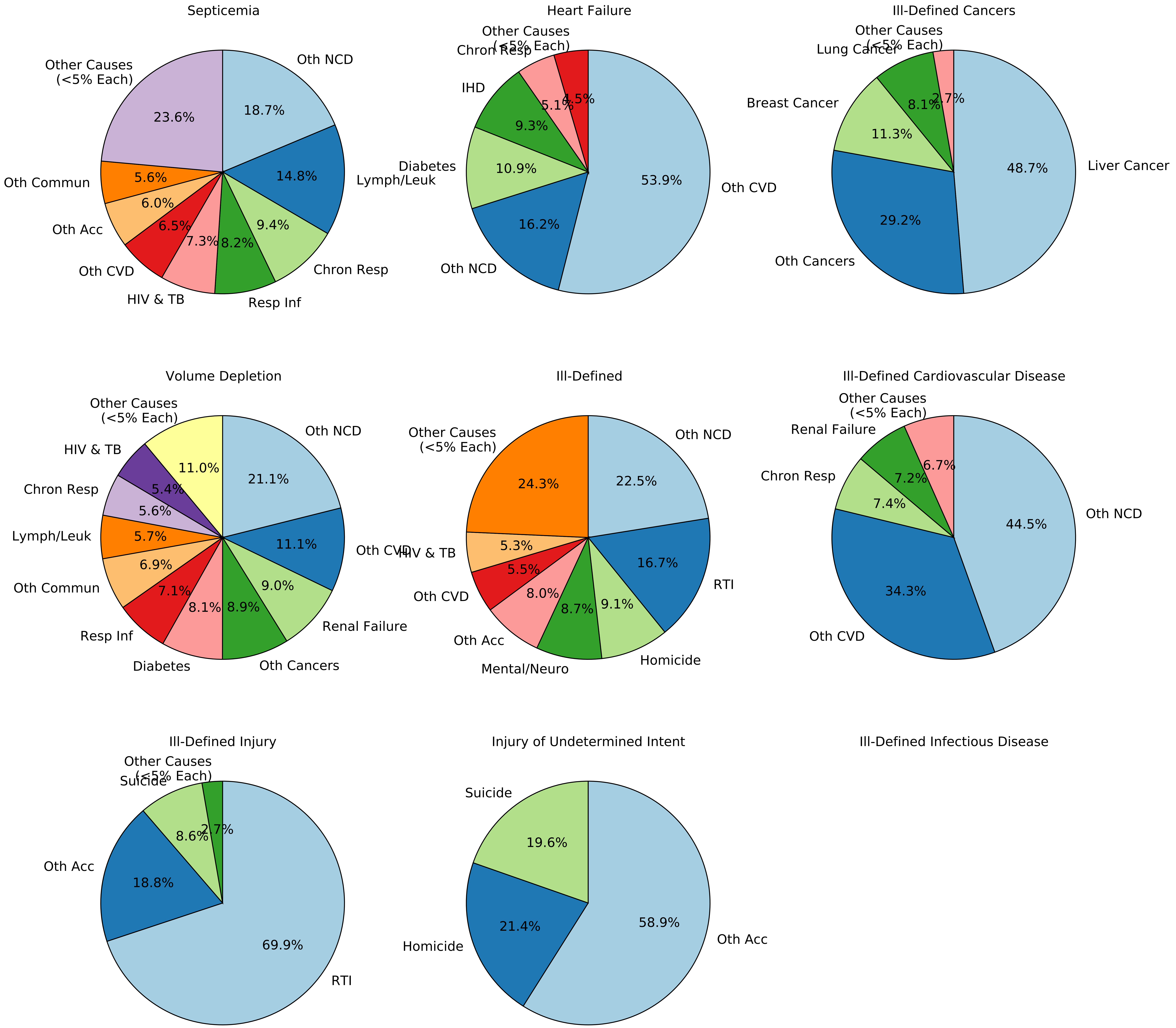

ICD 9
Female, Age 20

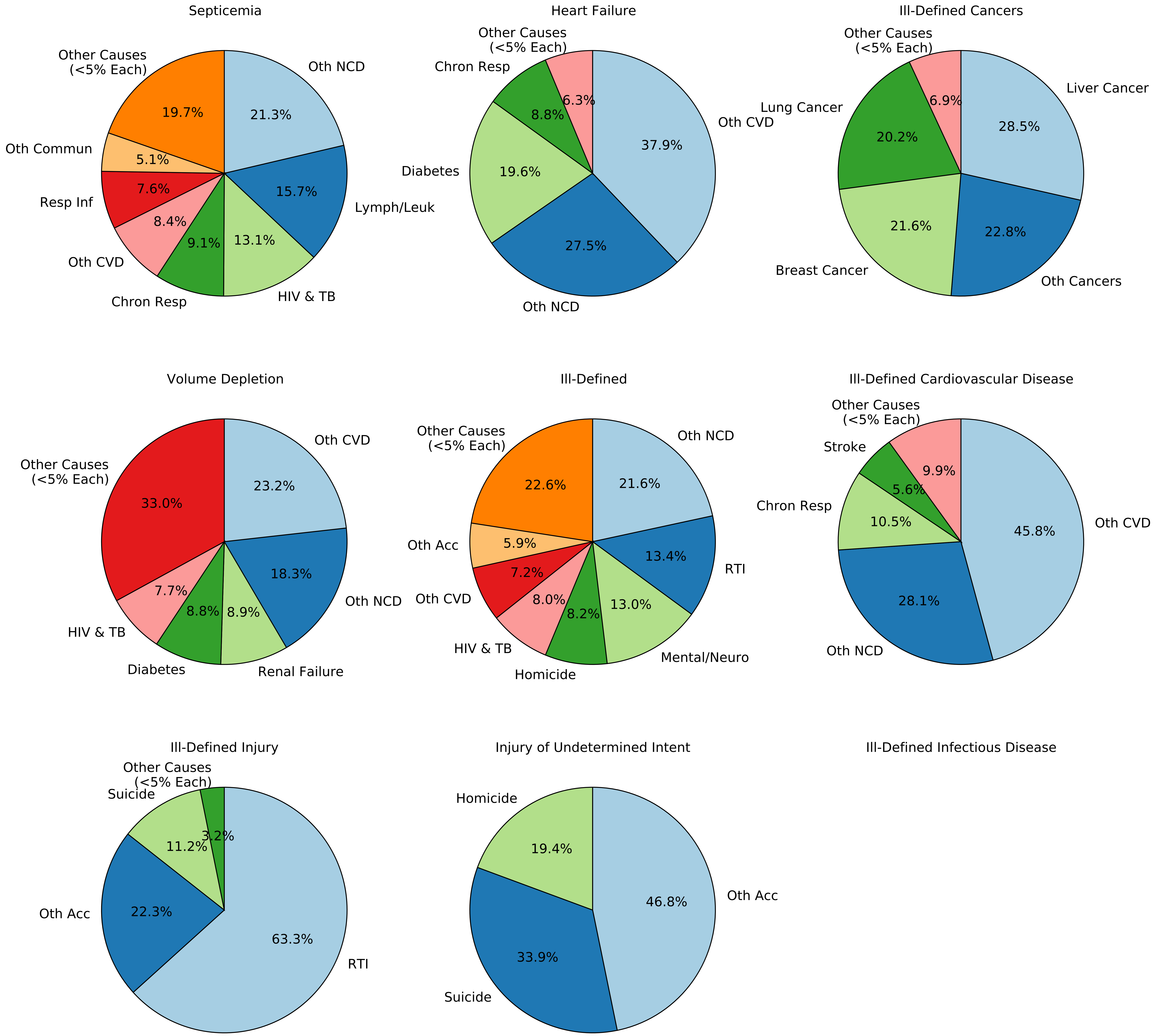

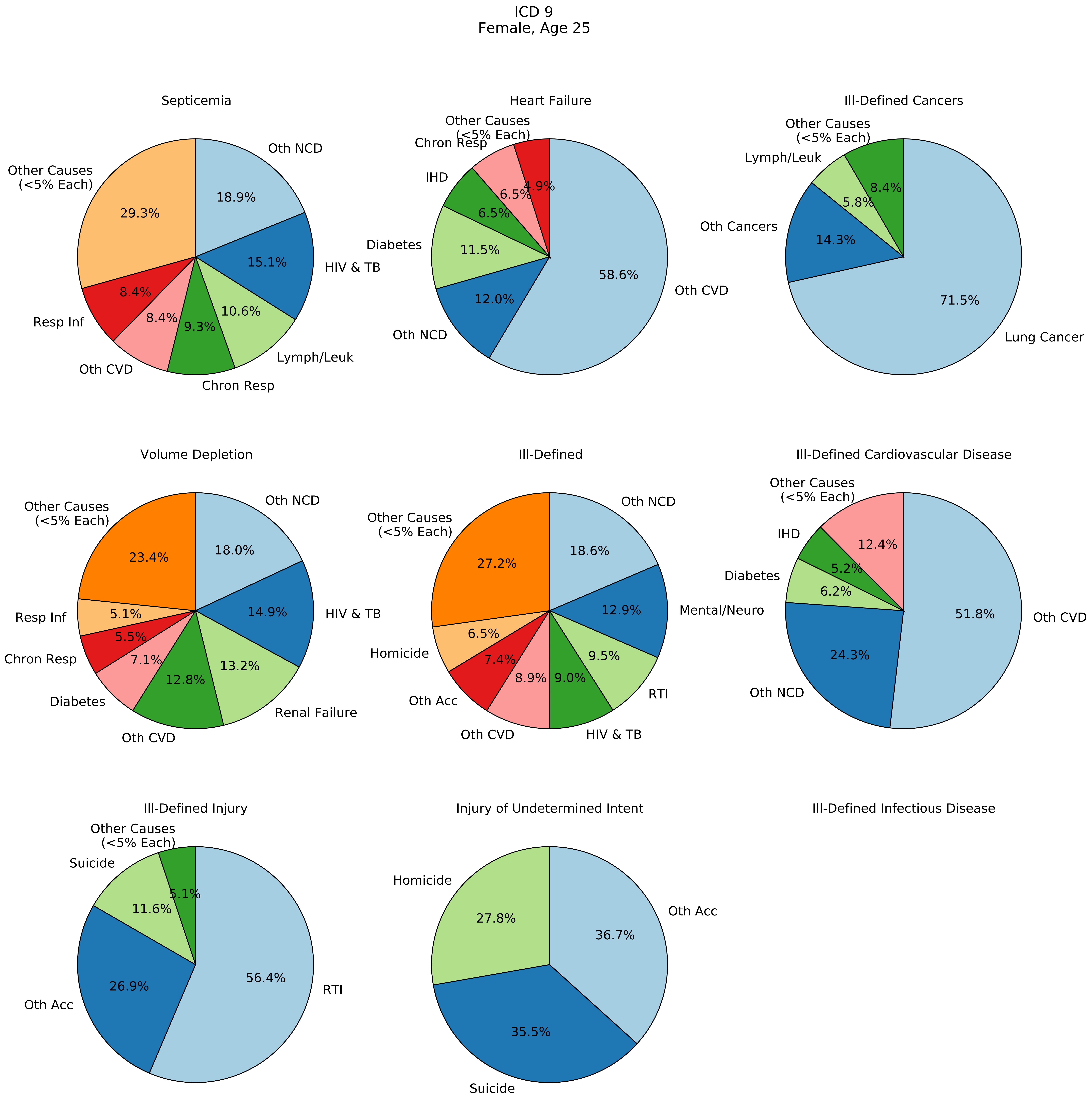
ICD 9
Female, Age 25
Septicemia
Oth NCD 18.9%
HIV & TB 15.1%
Lymph/Leuk 10.6%
Chron Resp 9.3%
Oth CVD 8.4%
Resp Inf 8.4%
Other Causes (<5% Each) 29.3%
Heart Failure
Oth CVD 58.6%
Oth NCD 12.0%
Diabetes 11.5%
IHD 6.5%
Chron Resp 6.5%
Other Causes (<5% Each) 4.9%
Ill-Defined Cancers
Lung Cancer 71.5%
Oth Cancers 14.3%
Lymph/Leuk 5.8%
Other Causes (<5% Each) 8.4%
Volume Depletion
Oth NCD 18.0%
HIV & TB 14.9%
Renal Failure 13.2%
Oth CVD 12.8%
Diabetes 7.1%
Chron Resp 5.5%
Resp Inf 5.1%
Other Causes (<5% Each) 23.4%
Ill-Defined
Oth NCD 18.6%
Mental/Neuro 12.9%
RTI 9.5%
HIV & TB 9.0%
Oth CVD 8.9%
Oth Acc 7.4%
Homicide 6.5%
Other Causes (<5% Each) 27.2%
Ill-Defined Cardiovascular Disease
Oth CVD 51.8%
Oth NCD 24.3%
Diabetes 6.2%
IHD 5.2%
Other Causes (<5% Each) 12.4%
Ill-Defined Injury
RTI 56.4%
Oth Acc 26.9%
Suicide 11.6%
Other Causes (<5% Each) 5.1%
Injury of Undetermined Intent
Oth Acc 36.7%
Suicide 35.5%
Homicide 27.8%
Ill-Defined Infectious Disease

ICD 9
Female, Age 30

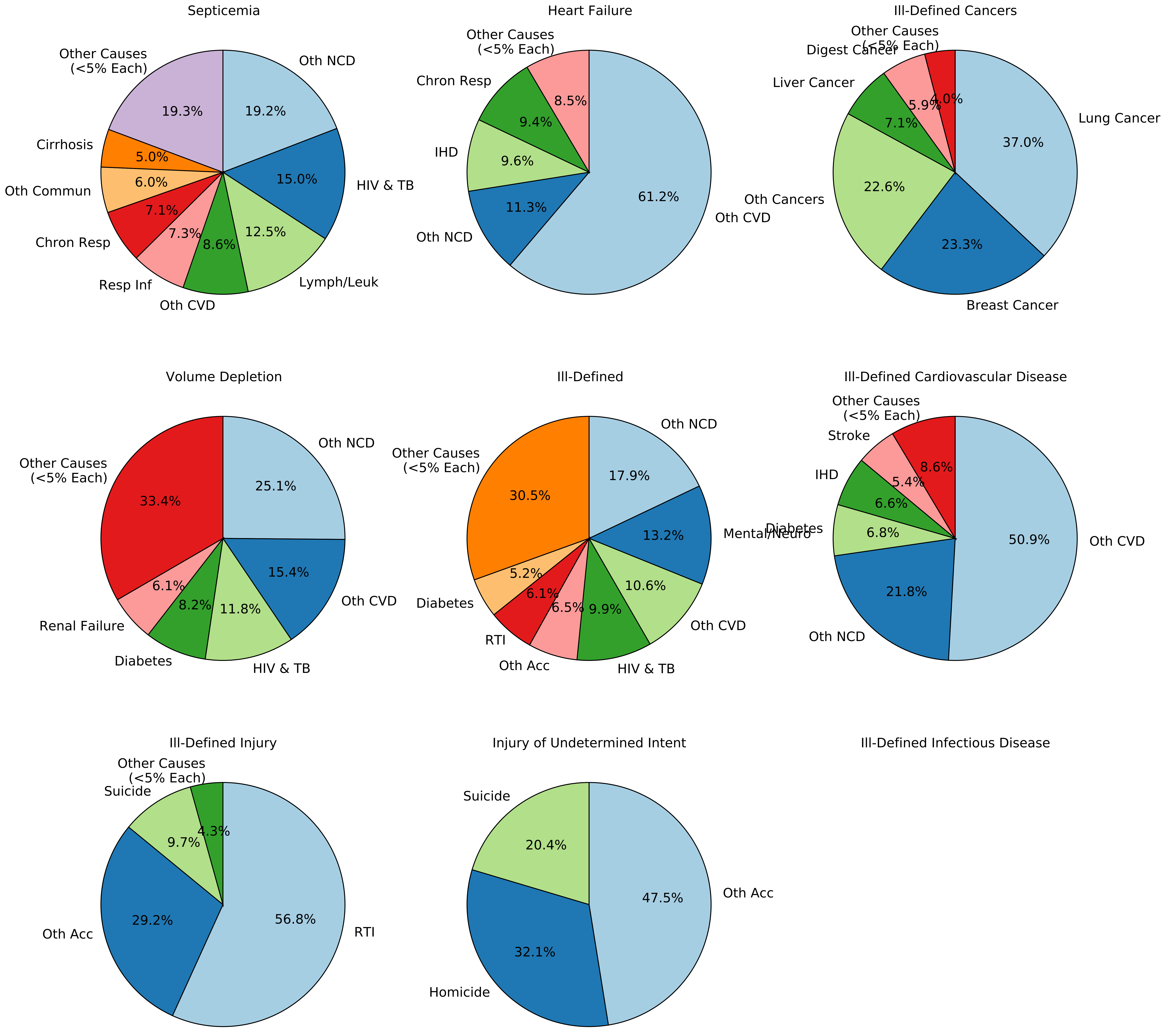

ICD 9
Female, Age 35

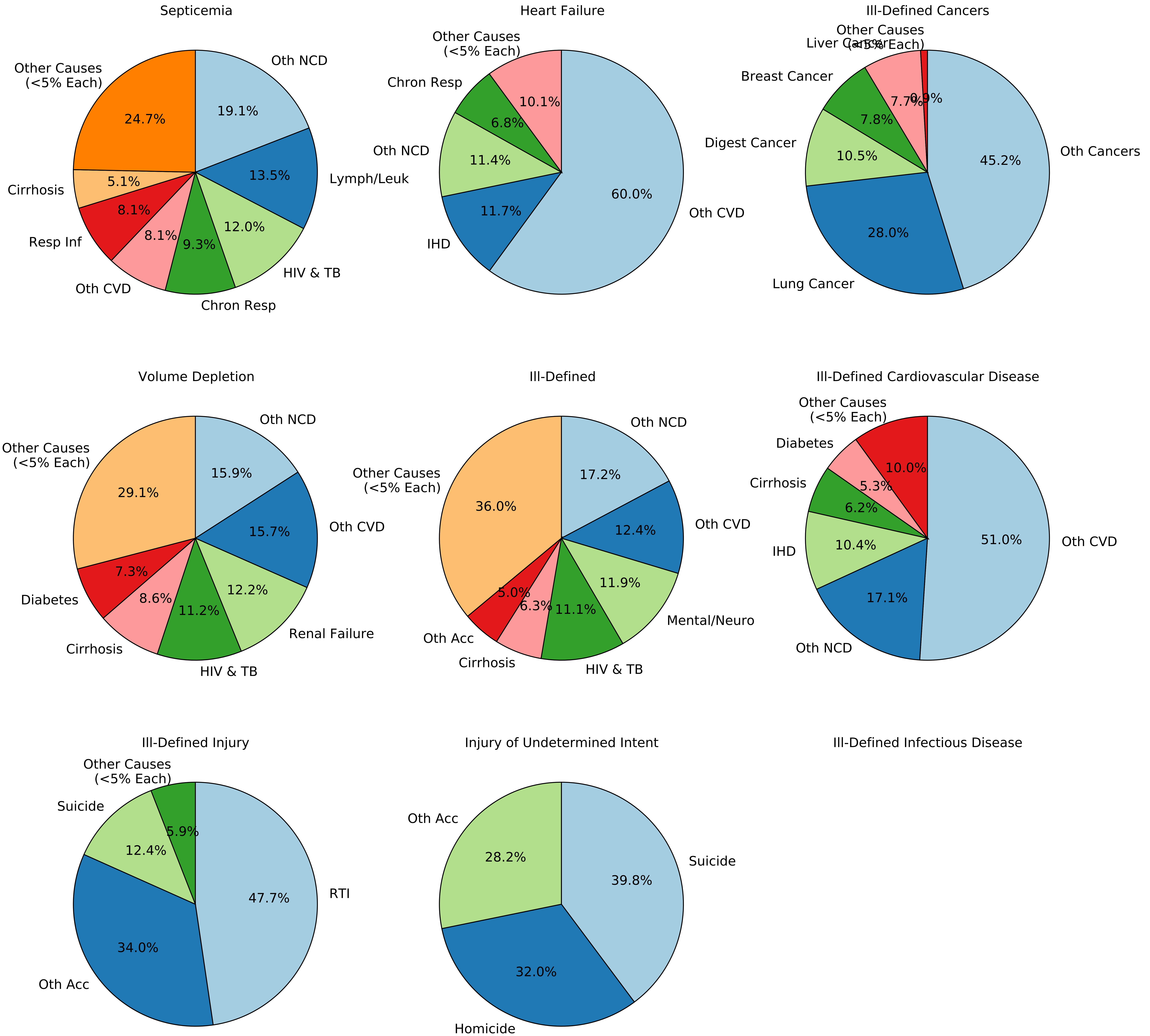

ICD 9
Female, Age 40

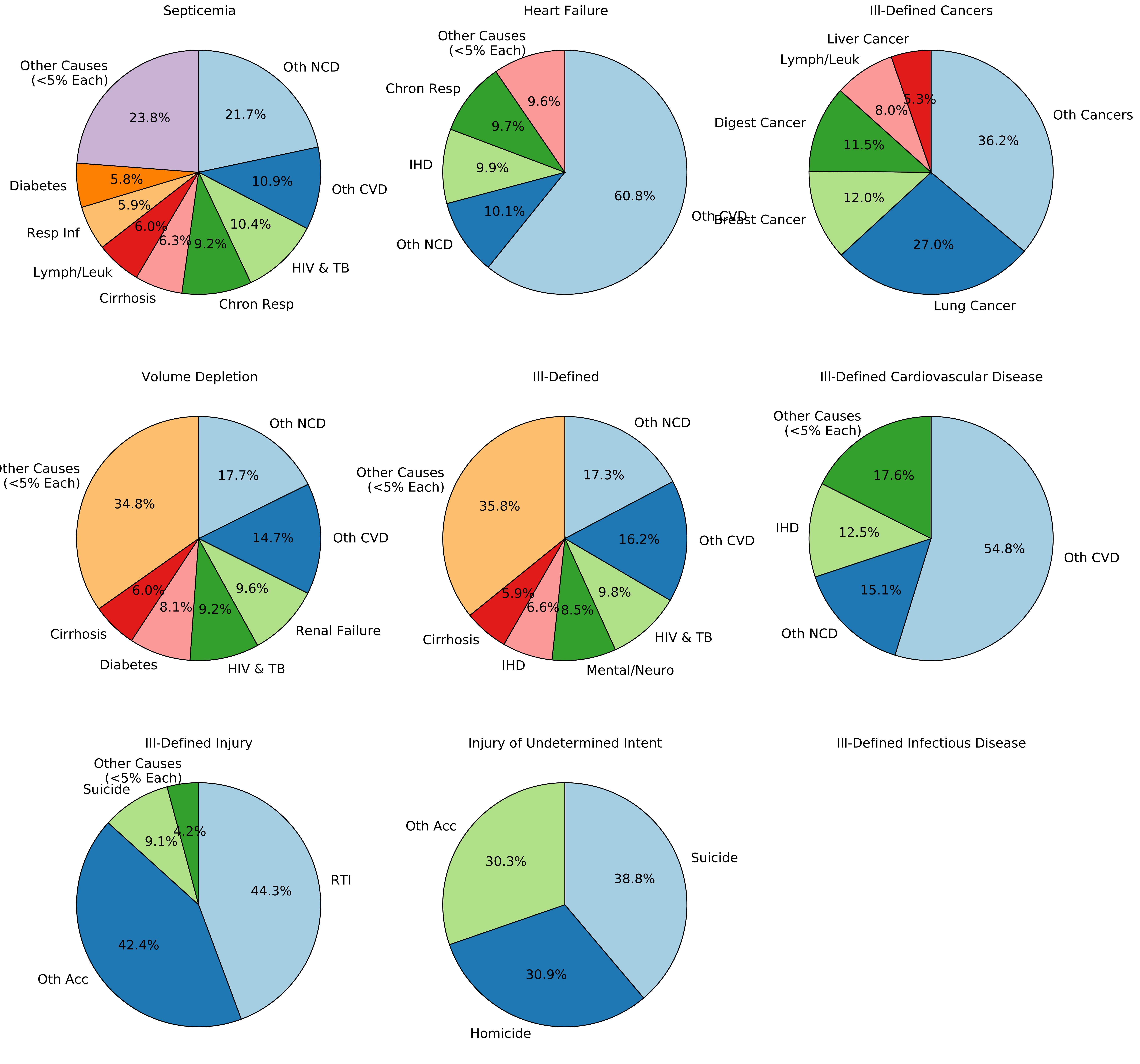

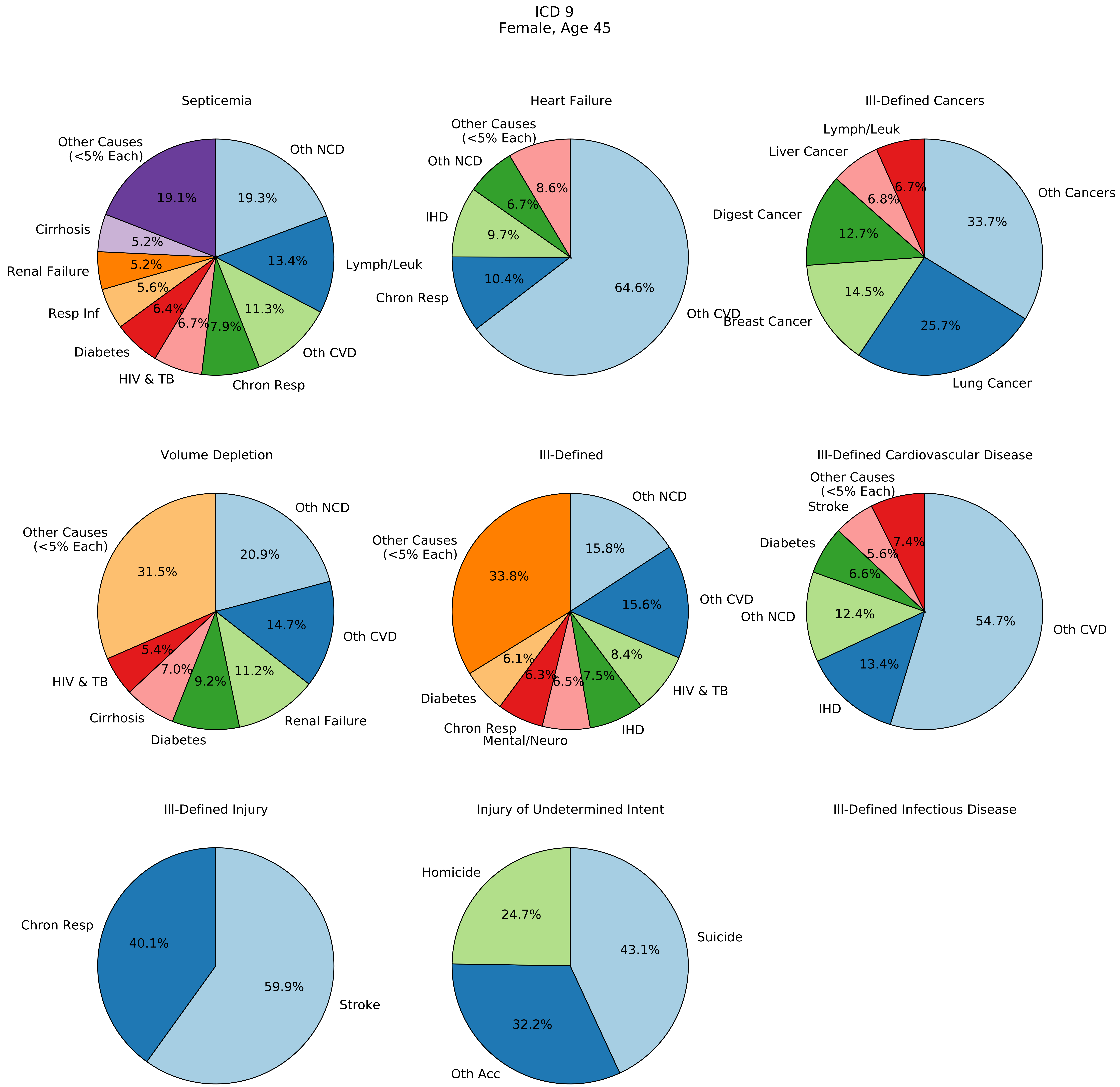
ICD 9
Female, Age 45
Septicemia
Oth NCD 19.3%
Lymph/Leuk 13.4%
Oth CVD 11.3%
Chron Resp 7.9%
HIV & TB 6.7%
Diabetes 6.4%
Resp Inf 5.6%
Renal Failure 5.2%
Cirrhosis 5.2%
Other Causes (<5% Each) 19.1%
Heart Failure
Oth CVD 64.6%
Chron Resp 10.4%
IHD 9.7%
Oth NCD 6.7%
Other Causes (<5% Each) 8.6%
Ill-Defined Cancers
Oth Cancers 33.7%
Lung Cancer 25.7%
Breast Cancer 14.5%
Digest Cancer 12.7%
Liver Cancer 6.8%
Lymph/Leuk 6.7%
Volume Depletion
Oth NCD 20.9%
Oth CVD 14.7%
Renal Failure 11.2%
Diabetes 9.2%
Cirrhosis 7.0%
HIV & TB 5.4%
Other Causes (<5% Each) 31.5%
Ill-Defined
Oth NCD 15.8%
Oth CVD 15.6%
HIV & TB 8.4%
IHD 7.5%
Mental/Neuro 6.5%
Chron Resp 6.3%
Diabetes 6.1%
Other Causes (<5% Each) 33.8%
Ill-Defined Cardiovascular Disease
Oth CVD 54.7%
IHD 13.4%
Oth NCD 12.4%
Diabetes 6.6%
Stroke 5.6%
Other Causes (<5% Each) 7.4%
Ill-Defined Injury
Stroke 59.9%
Chron Resp 40.1%
Injury of Undetermined Intent
Suicide 43.1%
Oth Acc 32.2%
Homicide 24.7%
Ill-Defined Infectious Disease

ICD 9
Female, Age 50

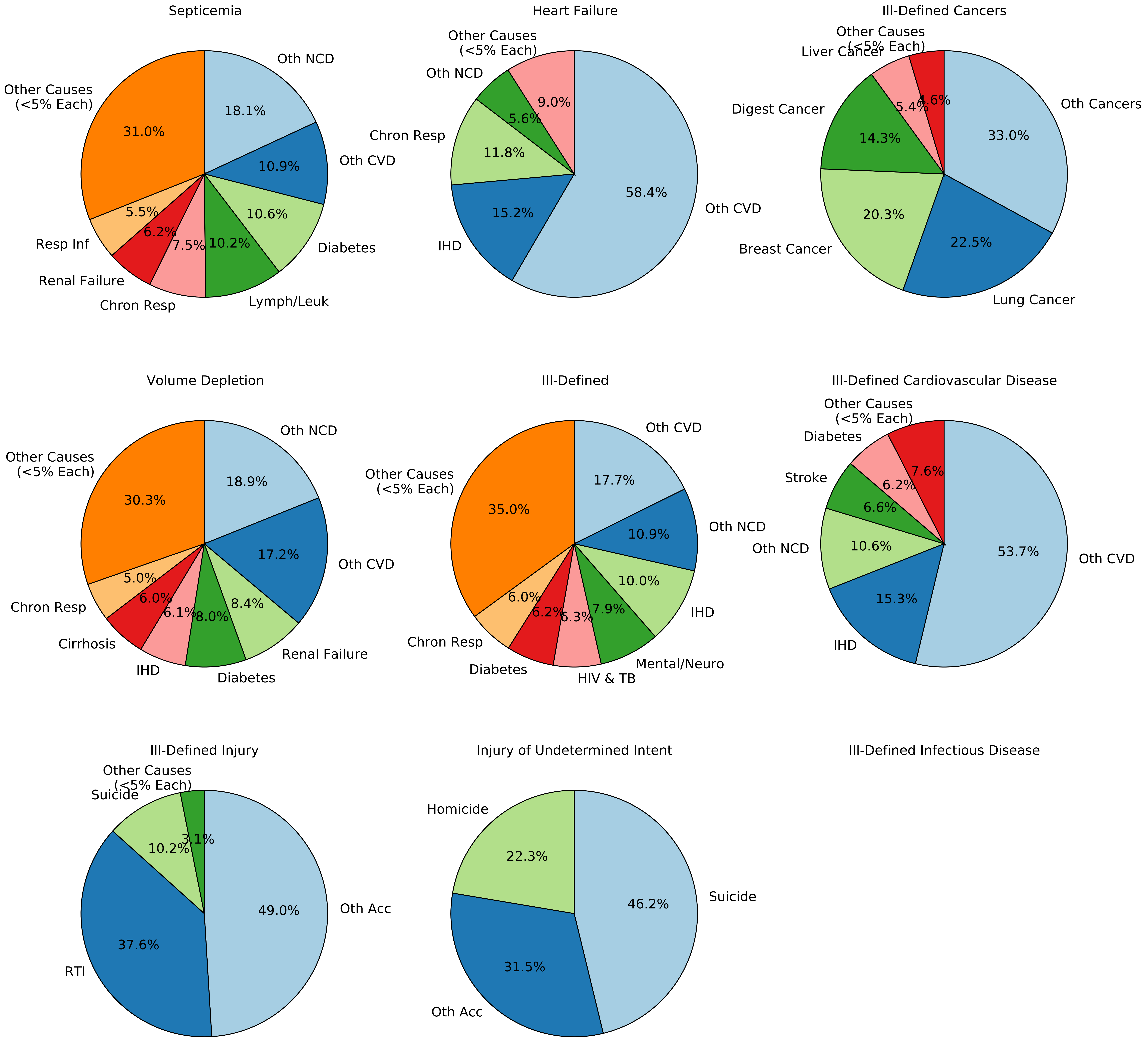

ICD 9
Female, Age 55

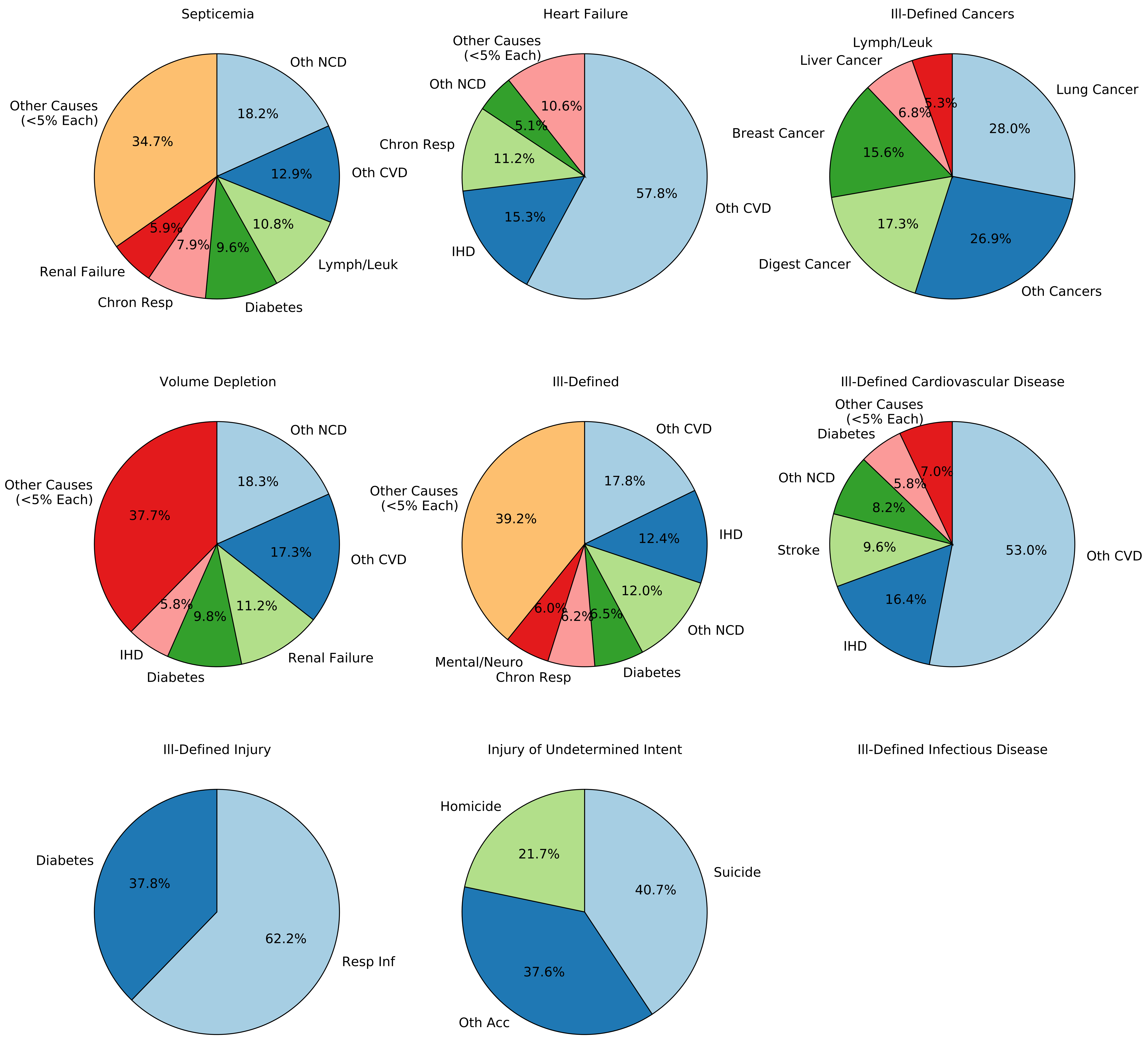

ICD 9
Female, Age 60

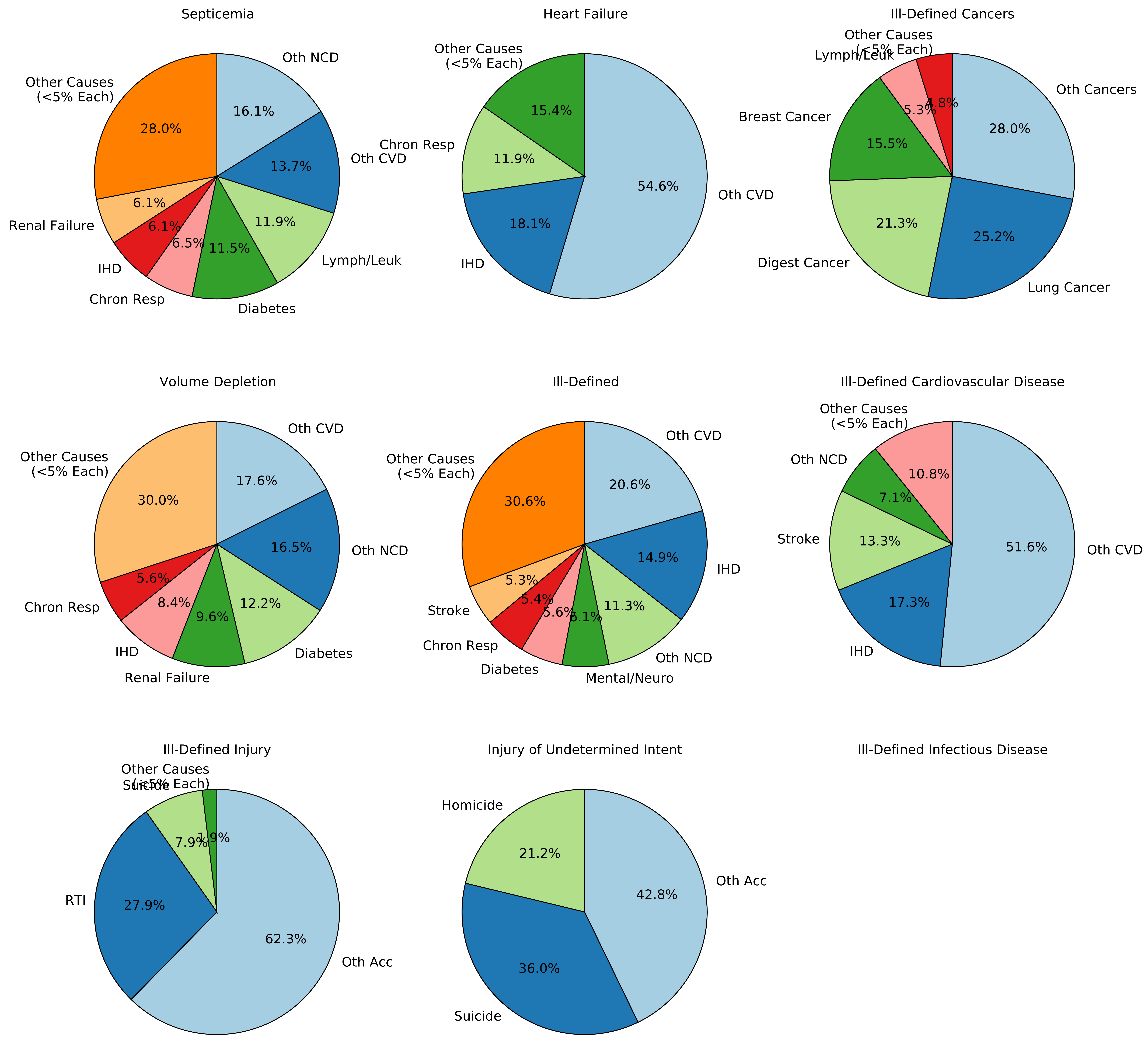

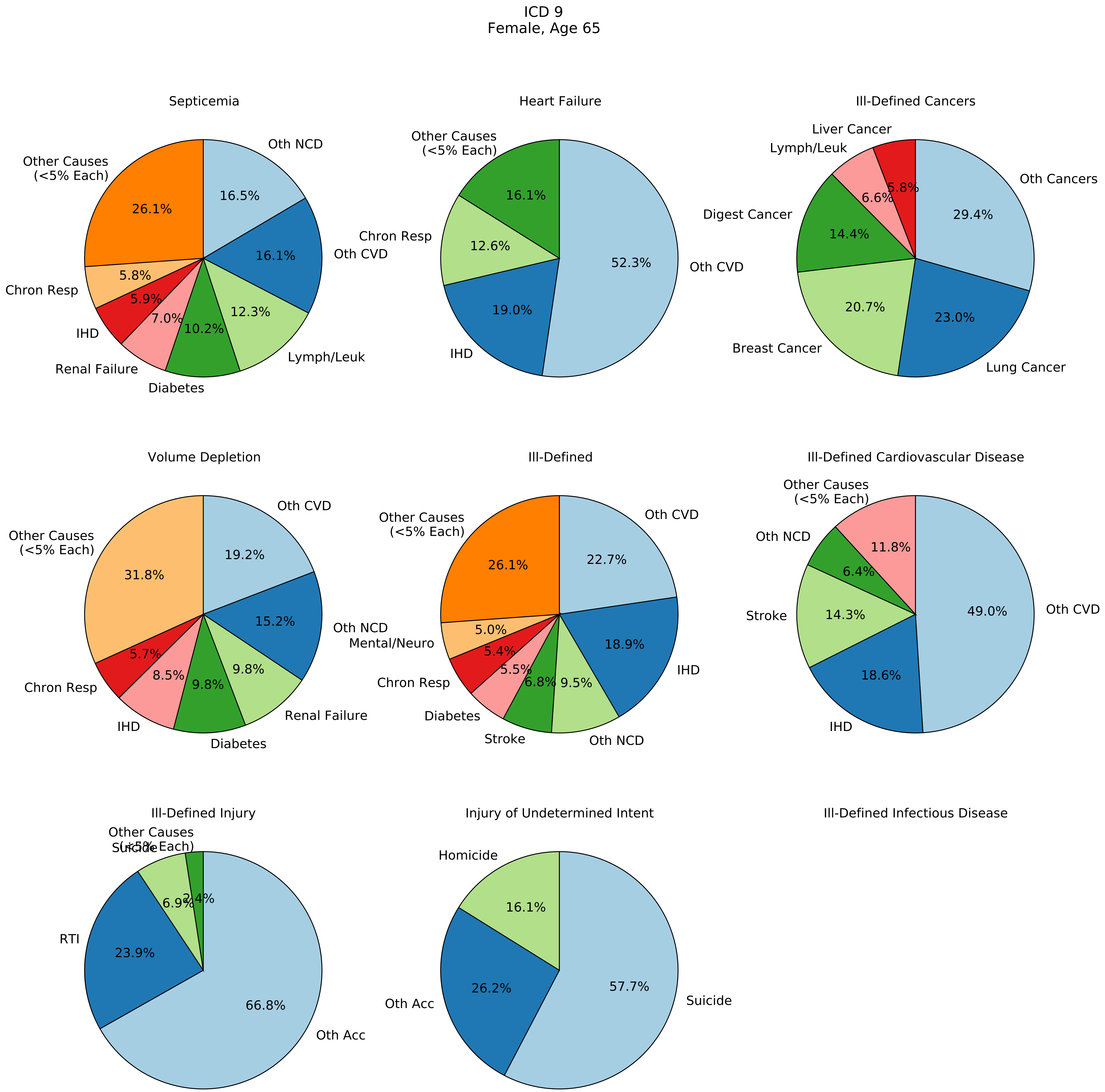
ICD 9
Female, Age 65
Septicemia
Oth NCD
16.5%
Oth CVD
16.1%
Lymph/Leuk
12.3%
Diabetes
10.2%
Renal Failure
7.0%
IHD
5.9%
Chron Resp
5.8%
Other Causes
(<5% Each)
26.1%
Heart Failure
Oth CVD
52.3%
IHD
19.0%
Chron Resp
12.6%
Other Causes
(<5% Each)
16.1%
Ill-Defined Cancers
Oth Cancers
29.4%
Lung Cancer
23.0%
Breast Cancer
20.7%
Digest Cancer
14.4%
Lymph/Leuk
6.6%
Liver Cancer
5.8%
Volume Depletion
Oth CVD
19.2%
Oth NCD
15.2%
Renal Failure
9.8%
Diabetes
9.8%
IHD
8.5%
Chron Resp
5.7%
Other Causes
(<5% Each)
31.8%
Ill-Defined
Oth CVD
22.7%
IHD
18.9%
Oth NCD
9.5%
Stroke
6.8%
Diabetes
5.5%
Chron Resp
5.4%
Mental/Neuro
5.0%
Other Causes
(<5% Each)
26.1%
Ill-Defined Cardiovascular Disease
Oth CVD
49.0%
IHD
18.6%
Stroke
14.3%
Oth NCD
6.4%
Other Causes
(<5% Each)
11.8%
Ill-Defined Injury
Oth Acc
66.8%
RTI
23.9%
Suicide
6.9%
Other Causes
(<5% Each)
2.4%
Injury of Undetermined Intent
Suicide
57.7%
Oth Acc
26.2%
Homicide
16.1%
Ill-Defined Infectious Disease

ICD 9
Female, Age 70

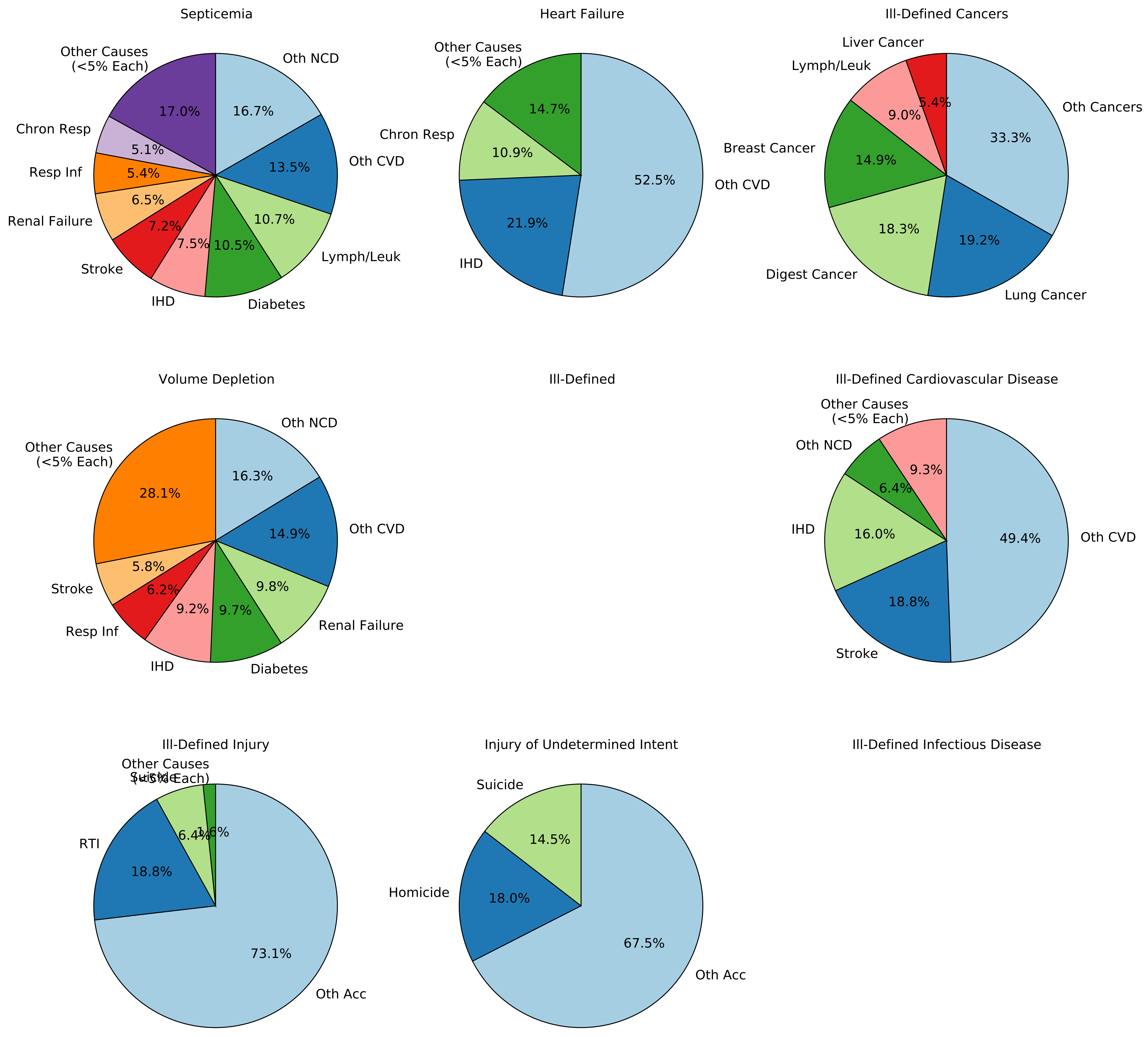

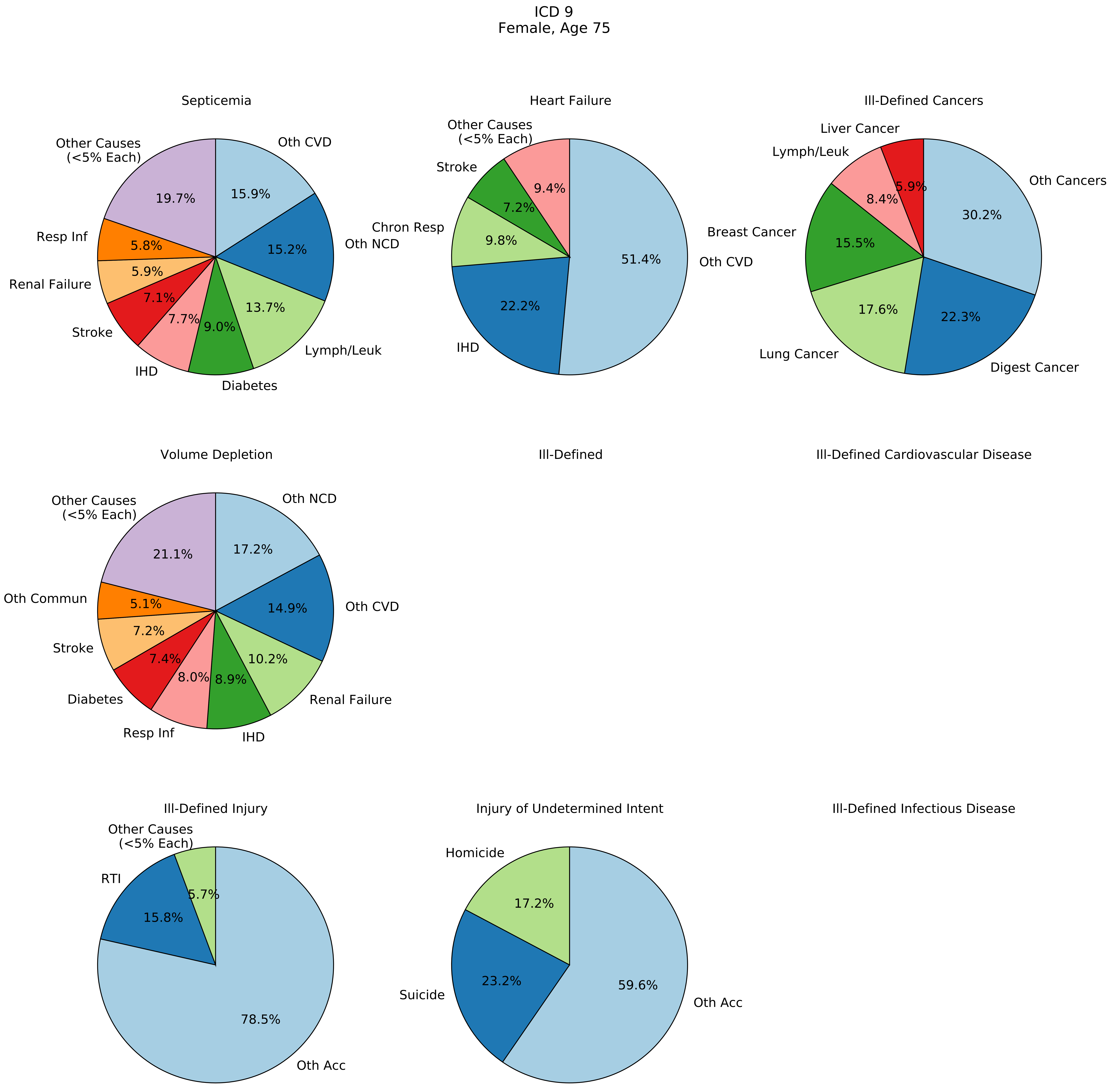
ICD 9
Female, Age 75
Septicemia
Other Causes (<5% Each)
19.7%
Oth CVD
15.9%
Oth NCD
15.2%
Lymph/Leuk
13.7%
Diabetes
9.0%
IHD
7.7%
Stroke
7.1%
Renal Failure
5.9%
Resp Inf
5.8%
Heart Failure
Other Causes (<5% Each)
9.4%
Oth CVD
51.4%
IHD
22.2%
Chron Resp
9.8%
Stroke
7.2%
Ill-Defined Cancers
Liver Cancer
5.9%
Oth Cancers
30.2%
Digest Cancer
22.3%
Lung Cancer
17.6%
Breast Cancer
15.5%
Lymph/Leuk
8.4%
Volume Depletion
Other Causes (<5% Each)
21.1%
Oth NCD
17.2%
Oth CVD
14.9%
Renal Failure
10.2%
IHD
8.9%
Resp Inf
8.0%
Diabetes
7.4%
Stroke
7.2%
Oth Commun
5.1%
Ill-Defined
Ill-Defined Cardiovascular Disease
Ill-Defined Injury
Other Causes (<5% Each)
5.7%
RTI
15.8%
Oth Acc
78.5%
Injury of Undetermined Intent
Homicide
17.2%
Suicide
23.2%
Oth Acc
59.6%
Ill-Defined Infectious Disease

ICD 9
Female, Age 80

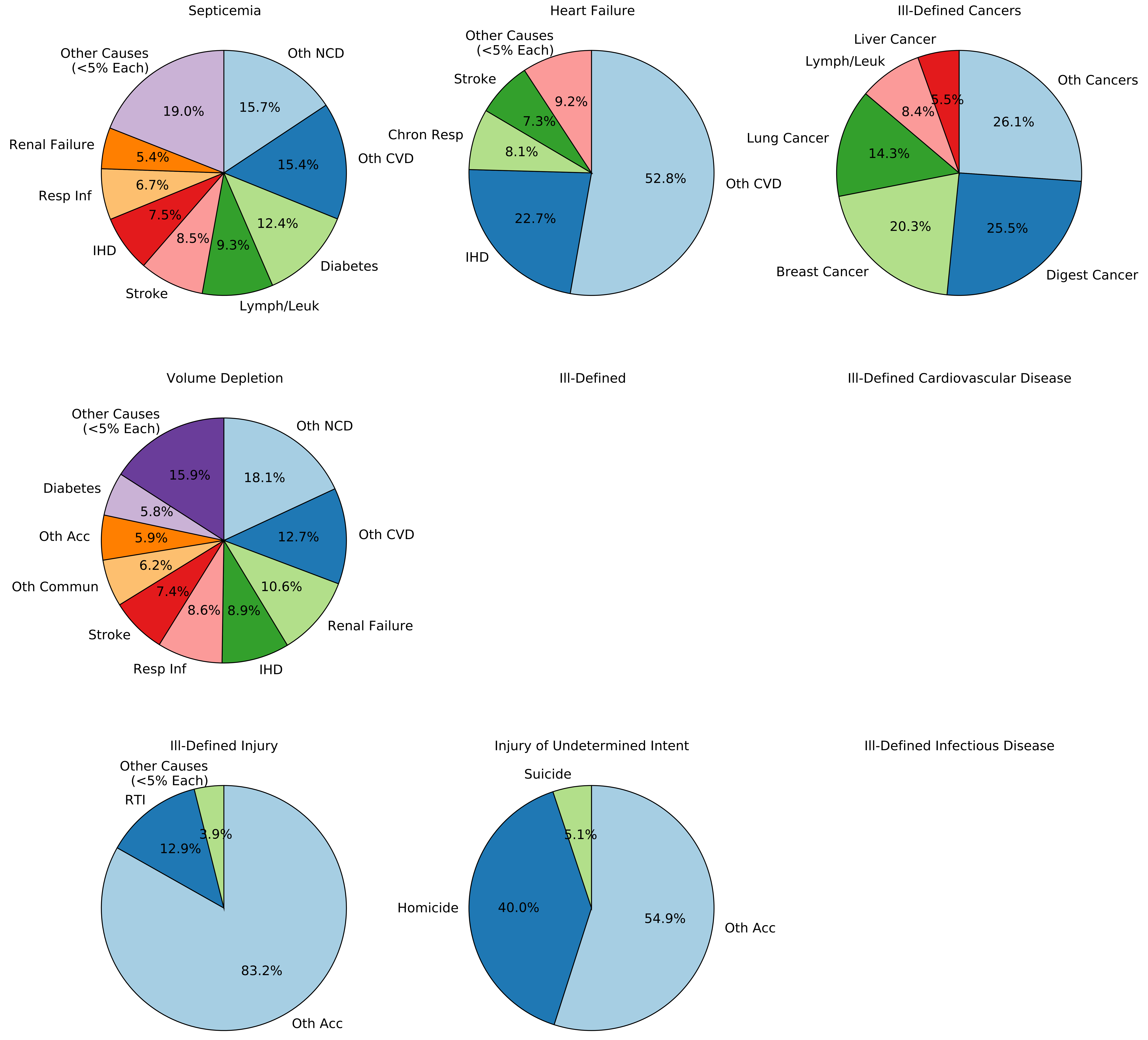

ICD 9
Female, Age 85

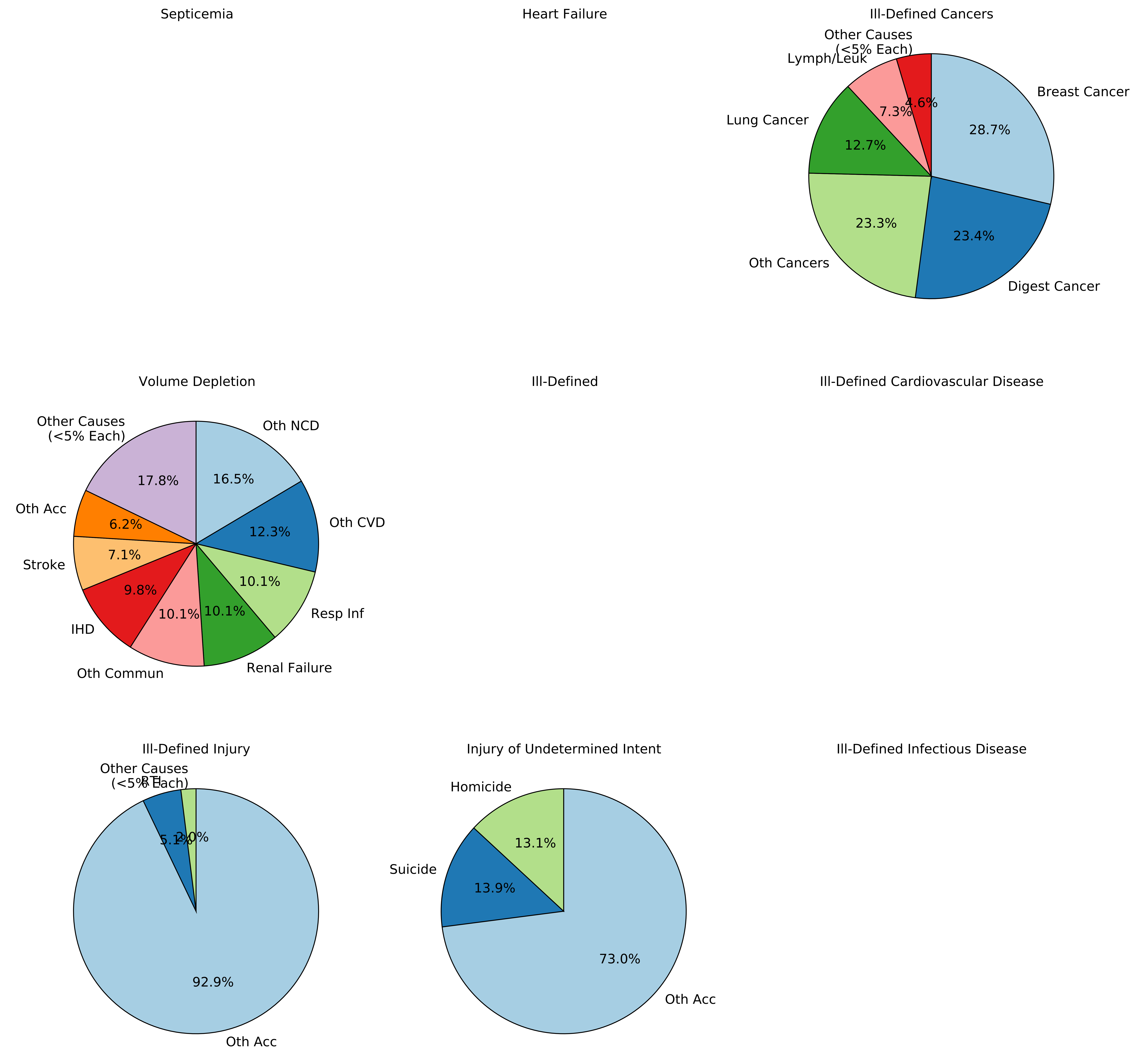

ICD 9
Male, Age 0

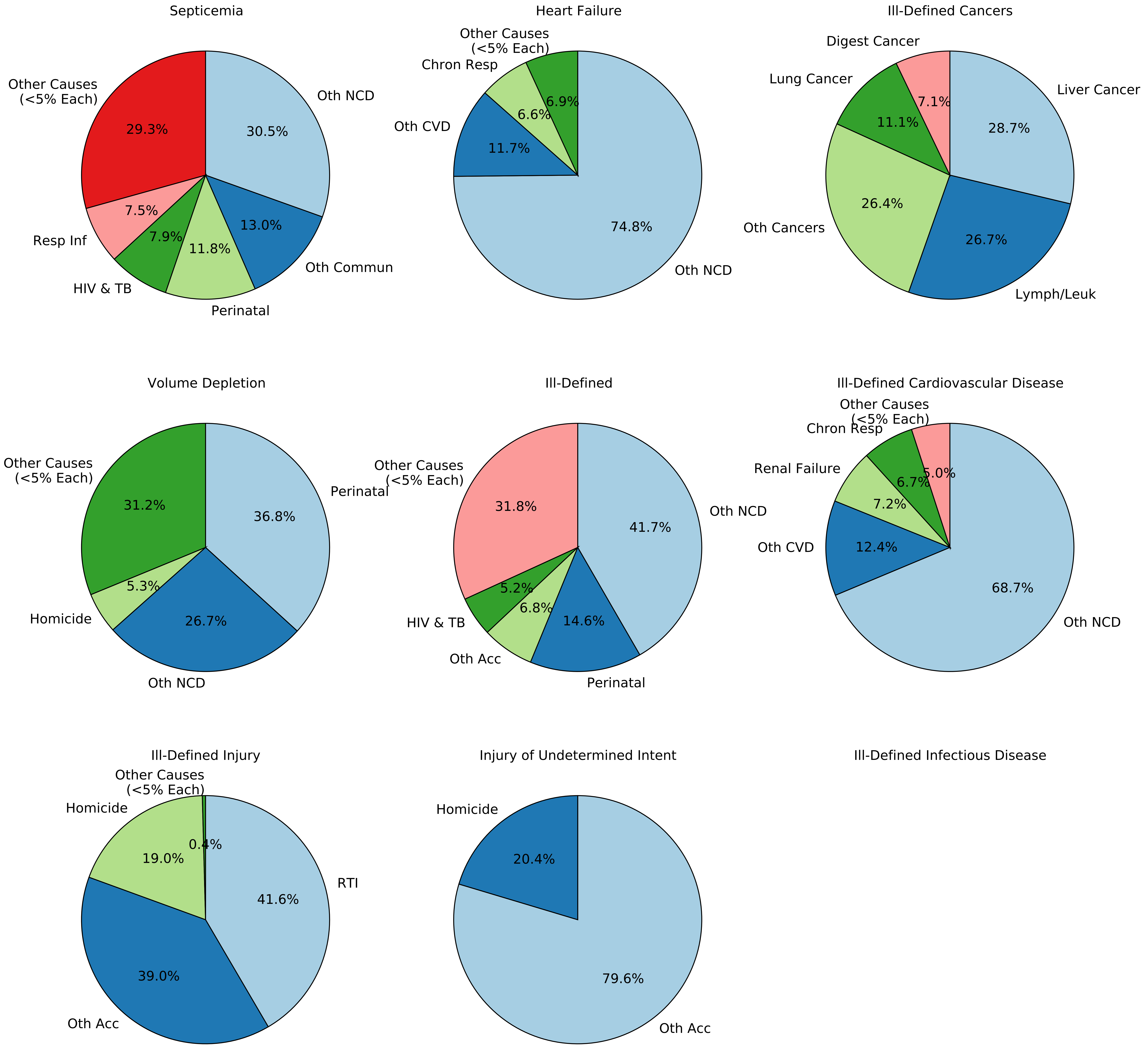

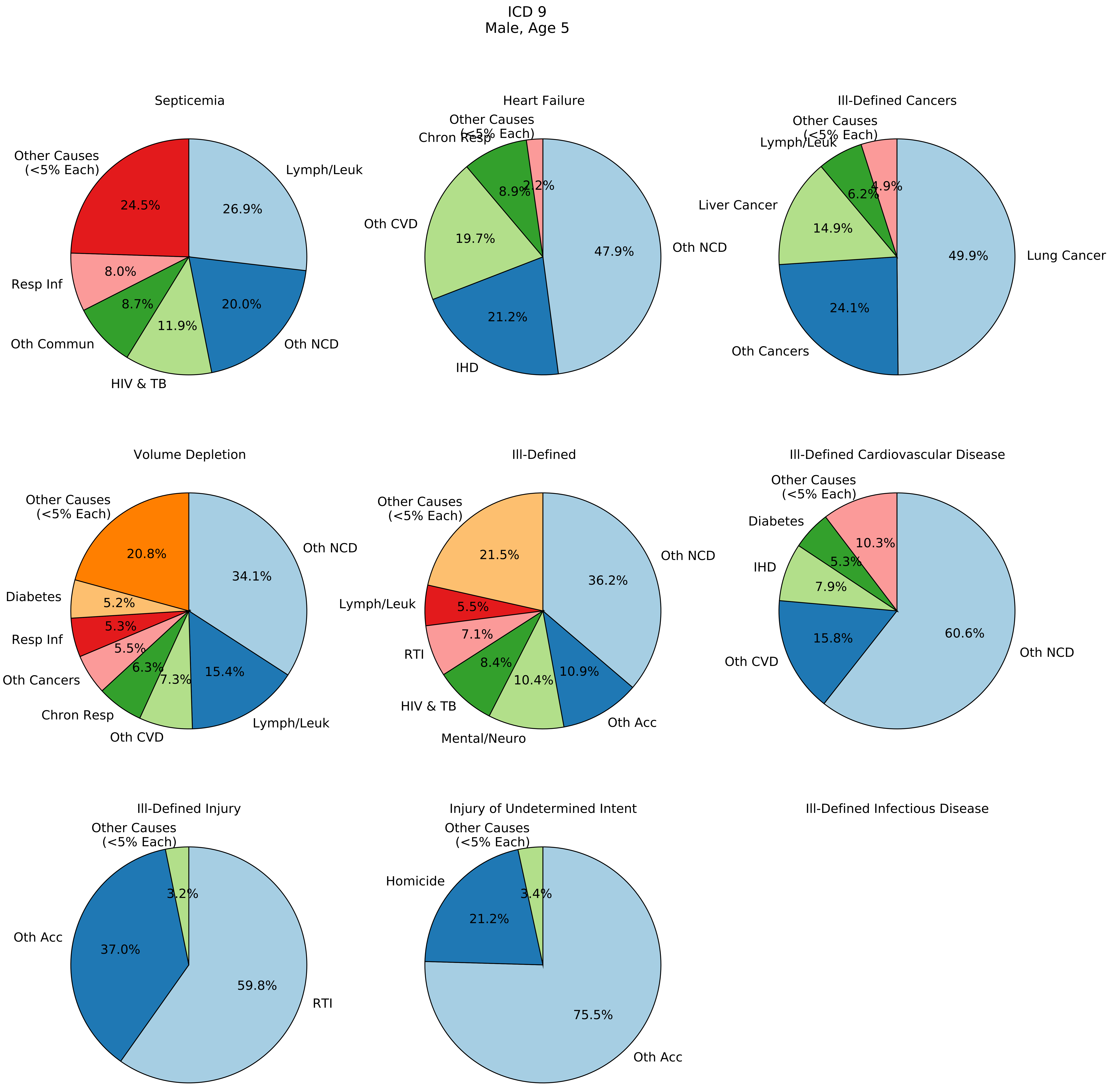

ICD 9
Male, Age 5
Septicemia
Other Causes (<5% Each) 24.5%
Lymph/Leuk 26.9%
Oth NCD 20.0%
HIV & TB 11.9%
Oth Commun 8.7%
Resp Inf 8.0%
Heart Failure
Other Causes (<5% Each) 2.2%
Chron Resp 8.9%
Oth CVD 19.7%
IHD 21.2%
Oth NCD 47.9%
Ill-Defined Cancers
Other Causes (<5% Each) 4.9%
Lymph/Leuk 6.2%
Liver Cancer 14.9%
Oth Cancers 24.1%
Lung Cancer 49.9%
Volume Depletion
Other Causes (<5% Each) 20.8%
Oth NCD 34.1%
Diabetes 5.2%
Resp Inf 5.3%
Oth Cancers 5.5%
Chron Resp 6.3%
Oth CVD 7.3%
Lymph/Leuk 15.4%
Ill-Defined
Other Causes (<5% Each) 21.5%
Oth NCD 36.2%
Lymph/Leuk 5.5%
RTI 7.1%
HIV & TB 8.4%
Mental/Neuro 10.4%
Oth Acc 10.9%
Ill-Defined Cardiovascular Disease
Other Causes (<5% Each) 10.3%
Diabetes 5.3%
IHD 7.9%
Oth CVD 15.8%
Oth NCD 60.6%
Ill-Defined Injury
Other Causes (<5% Each) 3.2%
Oth Acc 37.0%
RTI 59.8%
Injury of Undetermined Intent
Other Causes (<5% Each) 3.4%
Homicide 21.2%
Oth Acc 75.5%
Ill-Defined Infectious Disease

ICD 9
Male, Age 10

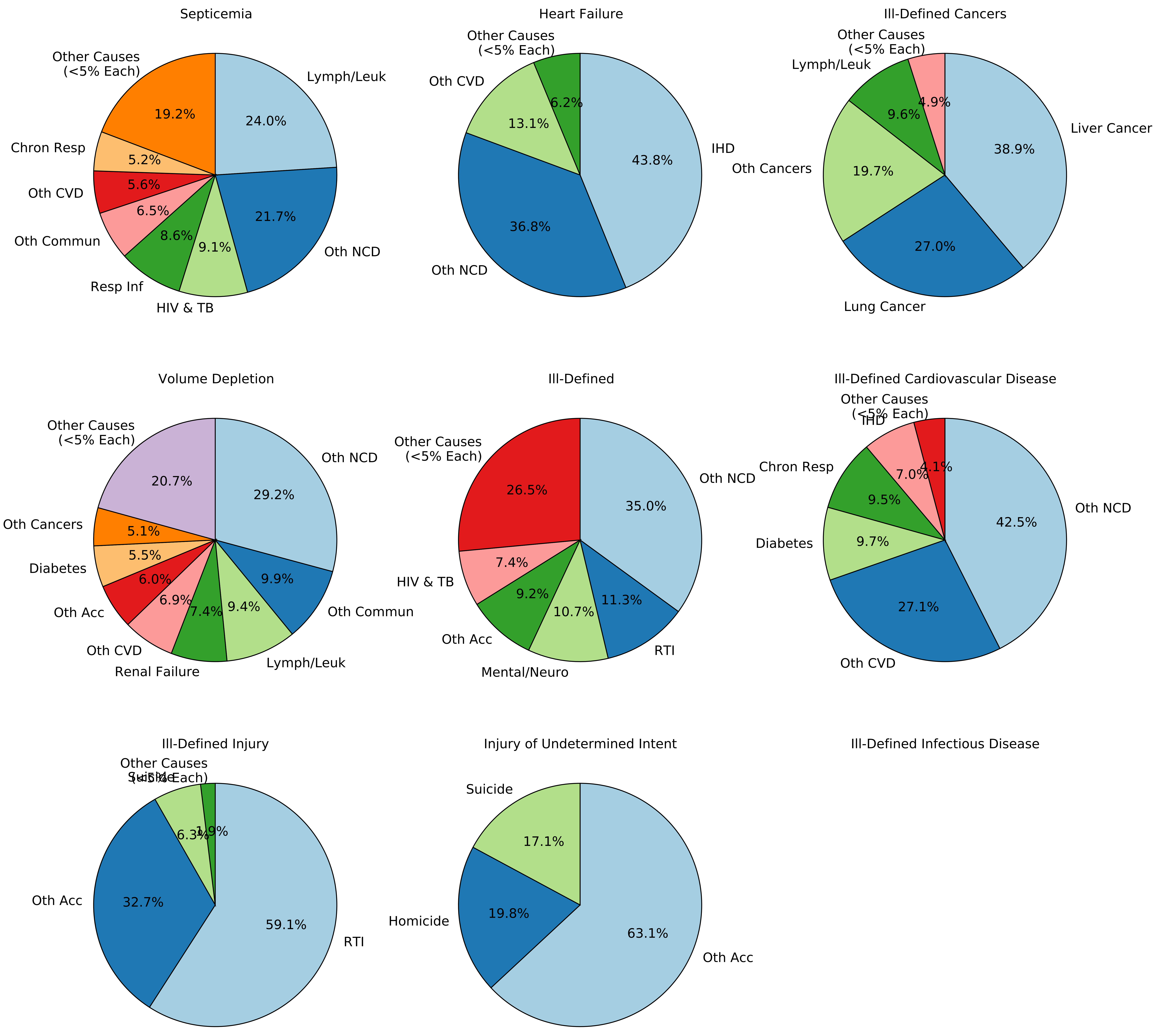

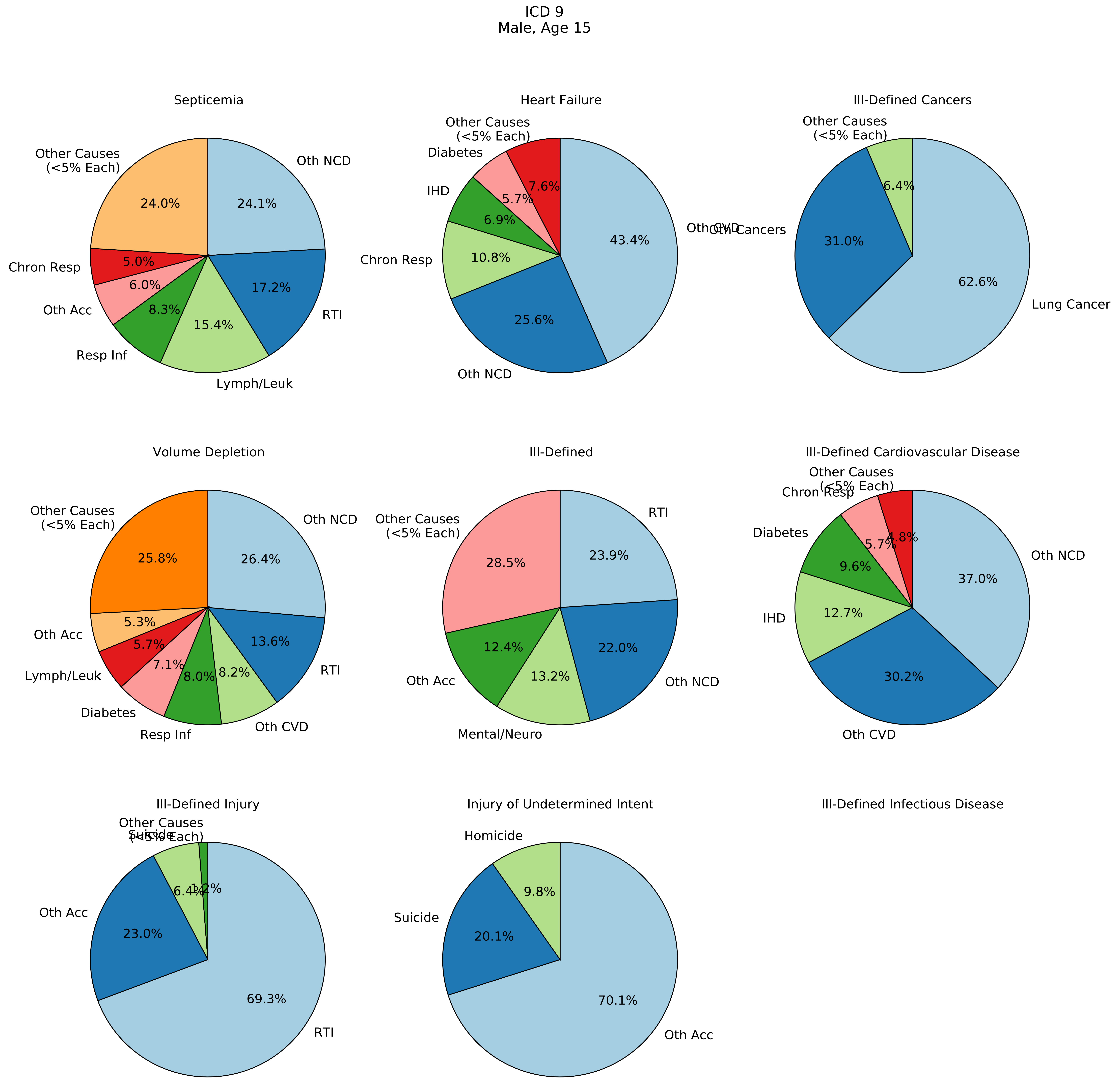
ICD 9
Male, Age 15
Septicemia
Other Causes (<5% Each)
24.0%
Oth NCD
24.1%
Chron Resp
5.0%
Oth Acc
6.0%
Resp Inf
8.3%
Lymph/Leuk
15.4%
RTI
17.2%
Heart Failure
Other Causes (<5% Each)
7.6%
Diabetes
5.7%
IHD
6.9%
Chron Resp
10.8%
Oth CVD
43.4%
Oth NCD
25.6%
Ill-Defined Cancers
Other Causes (<5% Each)
6.4%
Oth Cancers
31.0%
Lung Cancer
62.6%
Volume Depletion
Other Causes (<5% Each)
25.8%
Oth NCD
26.4%
Oth Acc
5.3%
Lymph/Leuk
5.7%
Diabetes
7.1%
Resp Inf
8.0%
Oth CVD
8.2%
RTI
13.6%
Ill-Defined
Other Causes (<5% Each)
28.5%
RTI
23.9%
Oth Acc
12.4%
Mental/Neuro
13.2%
Oth NCD
22.0%
Ill-Defined Cardiovascular Disease
Other Causes (<5% Each)
4.8%
Chron Resp
5.7%
Diabetes
9.6%
IHD
12.7%
Oth CVD
30.2%
Oth NCD
37.0%
Ill-Defined Injury
Other Causes (<5% Each)
1.2%
Suicide
6.4%
Oth Acc
23.0%
RTI
69.3%
Injury of Undetermined Intent
Homicide
9.8%
Suicide
20.1%
Oth Acc
70.1%
Ill-Defined Infectious Disease

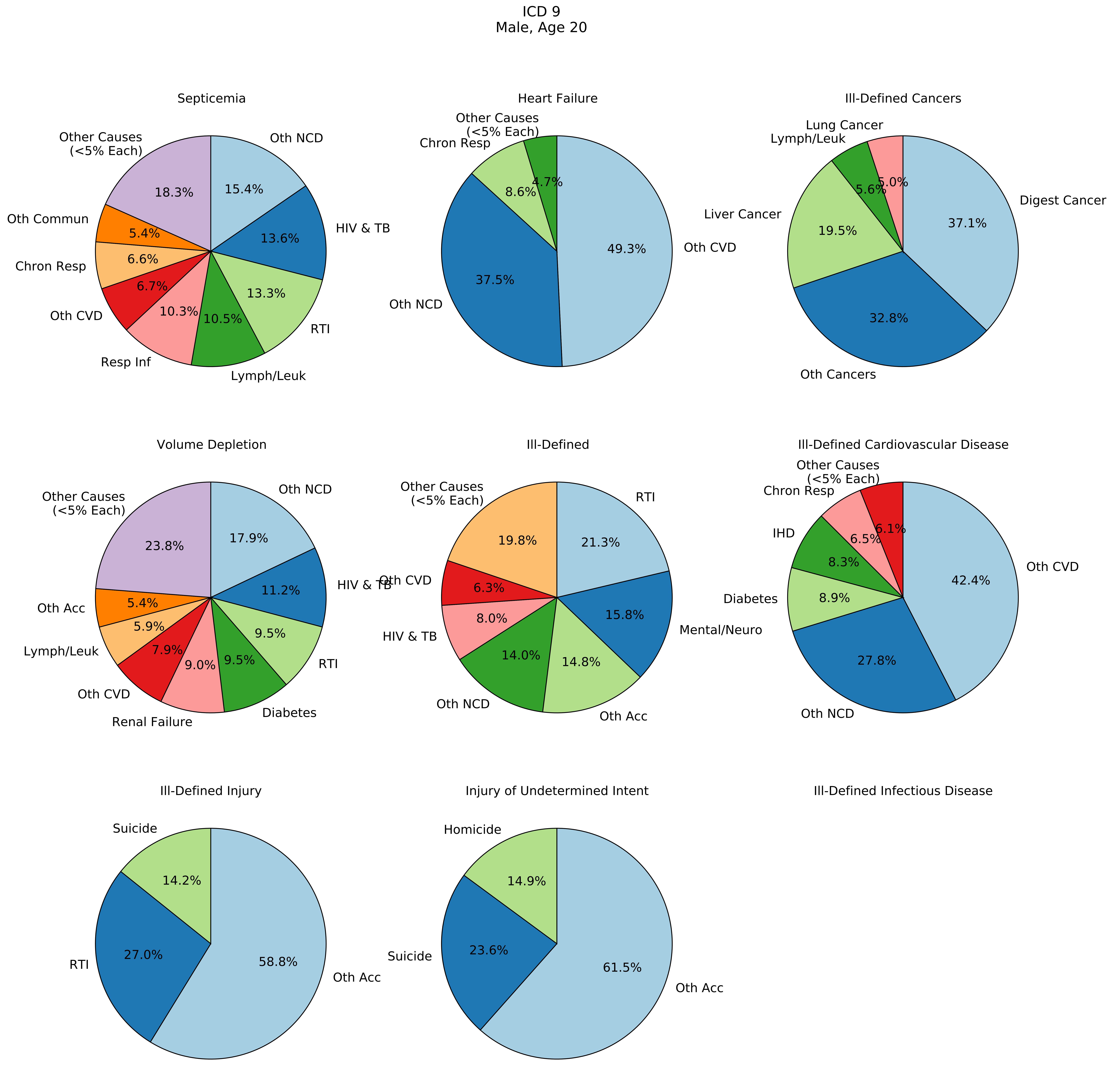

ICD 9
Male, Age 20
Septicemia
Oth NCD 15.4%
HIV & TB 13.6%
RTI 13.3%
Lymph/Leuk 10.5%
Resp Inf 10.3%
Oth CVD 6.7%
Chron Resp 6.6%
Oth Commun 5.4%
Other Causes (<5% Each) 18.3%
Heart Failure
Oth CVD 49.3%
Oth NCD 37.5%
Chron Resp 8.6%
Other Causes (<5% Each) 4.7%
Ill-Defined Cancers
Digest Cancer 37.1%
Oth Cancers 32.8%
Liver Cancer 19.5%
Lymph/Leuk 5.6%
Lung Cancer 5.0%
Volume Depletion
Oth NCD 17.9%
HIV & TB 11.2%
RTI 9.5%
Diabetes 9.5%
Renal Failure 9.0%
Oth CVD 7.9%
Lymph/Leuk 5.9%
Oth Acc 5.4%
Other Causes (<5% Each) 23.8%
Ill-Defined
RTI 21.3%
Mental/Neuro 15.8%
Oth Acc 14.8%
Oth NCD 14.0%
HIV & TB 8.0%
Oth CVD 6.3%
Other Causes (<5% Each) 19.8%
Ill-Defined Cardiovascular Disease
Oth CVD 42.4%
Oth NCD 27.8%
Diabetes 8.9%
IHD 8.3%
Chron Resp 6.5%
Other Causes (<5% Each) 6.1%
Ill-Defined Injury
Oth Acc 58.8%
RTI 27.0%
Suicide 14.2%
Injury of Undetermined Intent
Oth Acc 61.5%
Suicide 23.6%
Homicide 14.9%
Ill-Defined Infectious Disease

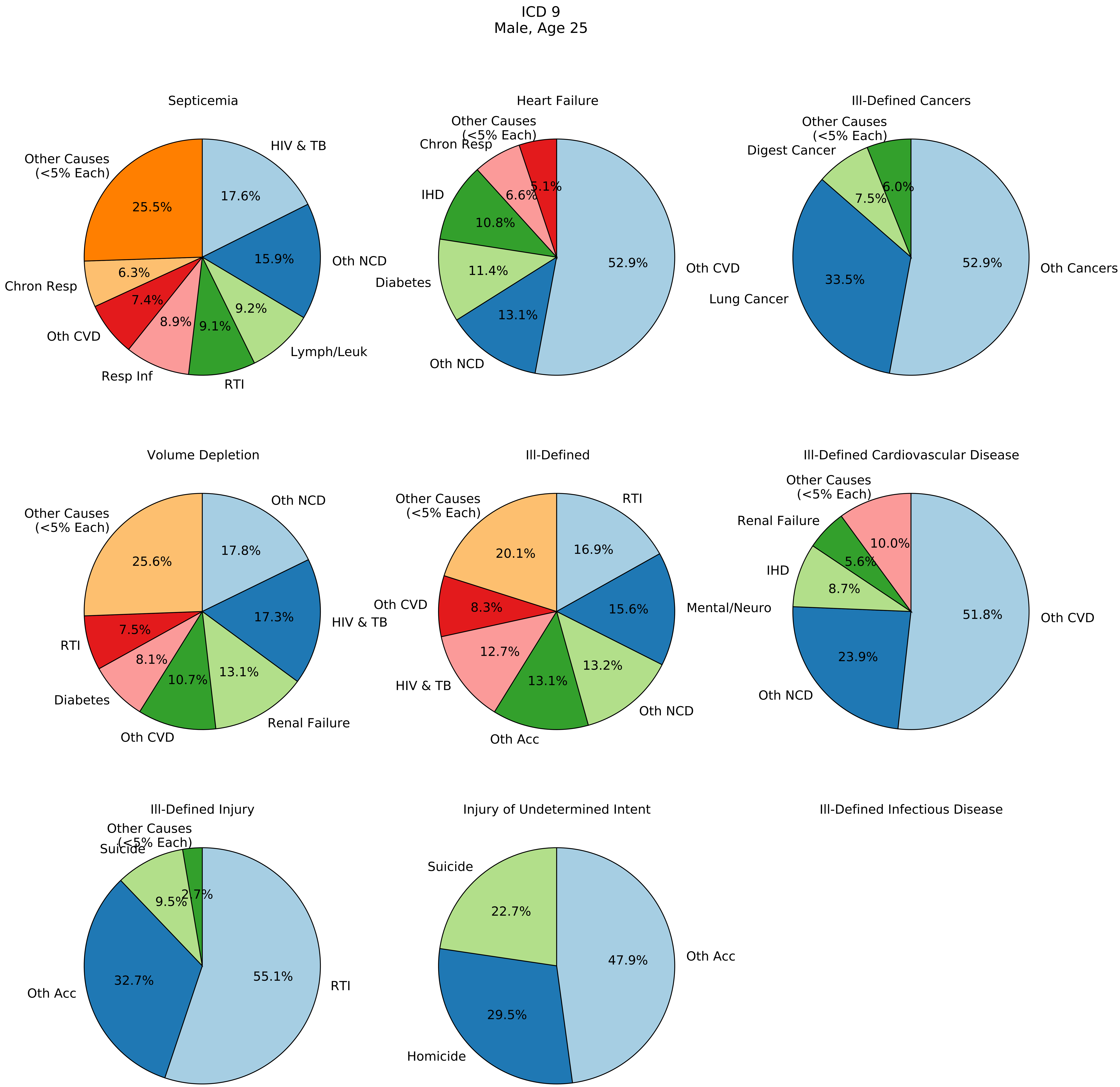

ICD 9
Male, Age 25
Septicemia
HIV & TB
17.6%
Oth NCD
15.9%
Lymph/Leuk
9.2%
RTI
9.1%
Resp Inf
8.9%
Oth CVD
7.4%
Chron Resp
6.3%
Other Causes (<5% Each)
25.5%
Heart Failure
Oth CVD
52.9%
Oth NCD
13.1%
Diabetes
11.4%
IHD
10.8%
Chron Resp
6.6%
Other Causes (<5% Each)
5.1%
Ill-Defined Cancers
Oth Cancers
52.9%
Lung Cancer
33.5%
Digest Cancer
7.5%
Other Causes (<5% Each)
6.0%
Volume Depletion
Oth NCD
17.8%
HIV & TB
17.3%
Renal Failure
13.1%
Oth CVD
10.7%
Diabetes
8.1%
RTI
7.5%
Other Causes (<5% Each)
25.6%
Ill-Defined
RTI
16.9%
Mental/Neuro
15.6%
Oth NCD
13.2%
Oth Acc
13.1%
HIV & TB
12.7%
Oth CVD
8.3%
Other Causes (<5% Each)
20.1%
Ill-Defined Cardiovascular Disease
Oth CVD
51.8%
Oth NCD
23.9%
IHD
8.7%
Renal Failure
5.6%
Other Causes (<5% Each)
10.0%
Ill-Defined Injury
RTI
55.1%
Oth Acc
32.7%
Suicide
9.5%
Other Causes (<5% Each)
2.7%
Injury of Undetermined Intent
Oth Acc
47.9%
Homicide
29.5%
Suicide
22.7%
Ill-Defined Infectious Disease

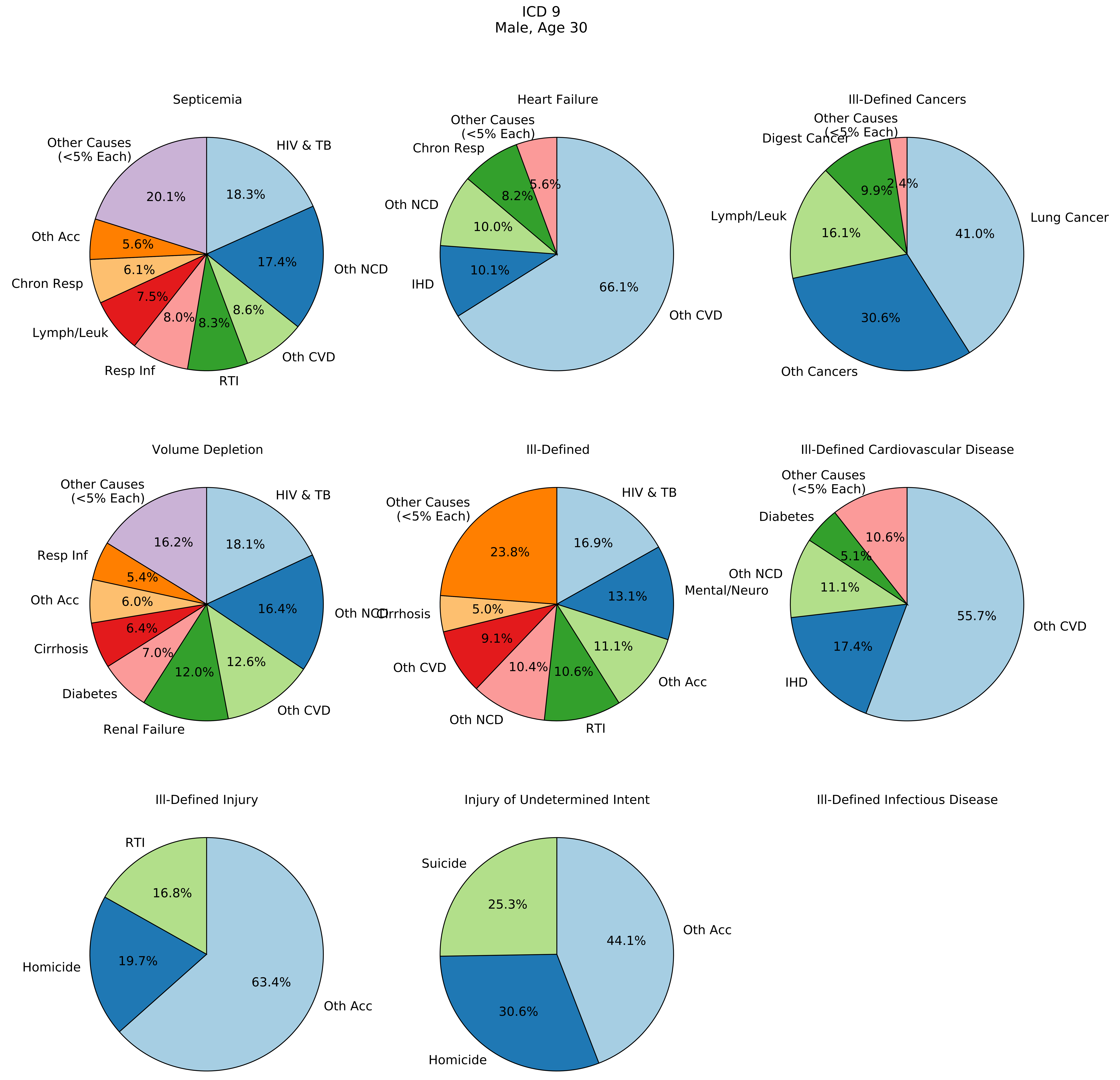
ICD 9
Male, Age 30
Septicemia
HIV & TB
18.3%
Oth NCD
17.4%
Oth CVD
8.6%
RTI
8.3%
Resp Inf
8.0%
Lymph/Leuk
7.5%
Chron Resp
6.1%
Oth Acc
5.6%
Other Causes
(<5% Each)
20.1%
Heart Failure
Oth CVD
66.1%
IHD
10.1%
Oth NCD
10.0%
Chron Resp
8.2%
Other Causes
(<5% Each)
5.6%
Ill-Defined Cancers
Lung Cancer
41.0%
Oth Cancers
30.6%
Lymph/Leuk
16.1%
Digest Cancer
9.9%
Other Causes
(<5% Each)
2.4%
Volume Depletion
HIV & TB
18.1%
Oth NCD
16.4%
Oth CVD
12.6%
Renal Failure
12.0%
Diabetes
7.0%
Cirrhosis
6.4%
Oth Acc
6.0%
Resp Inf
5.4%
Other Causes
(<5% Each)
16.2%
Ill-Defined
HIV & TB
16.9%
Mental/Neuro
13.1%
Oth Acc
11.1%
RTI
10.6%
Oth NCD
10.4%
Oth CVD
9.1%
Cirrhosis
5.0%
Other Causes
(<5% Each)
23.8%
Ill-Defined Cardiovascular Disease
Oth CVD
55.7%
IHD
17.4%
Oth NCD
11.1%
Diabetes
5.1%
Other Causes
(<5% Each)
10.6%
Ill-Defined Injury
Oth Acc
63.4%
Homicide
19.7%
RTI
16.8%
Injury of Undetermined Intent
Oth Acc
44.1%
Homicide
30.6%
Suicide
25.3%
Ill-Defined Infectious Disease

ICD 9
Male, Age 35

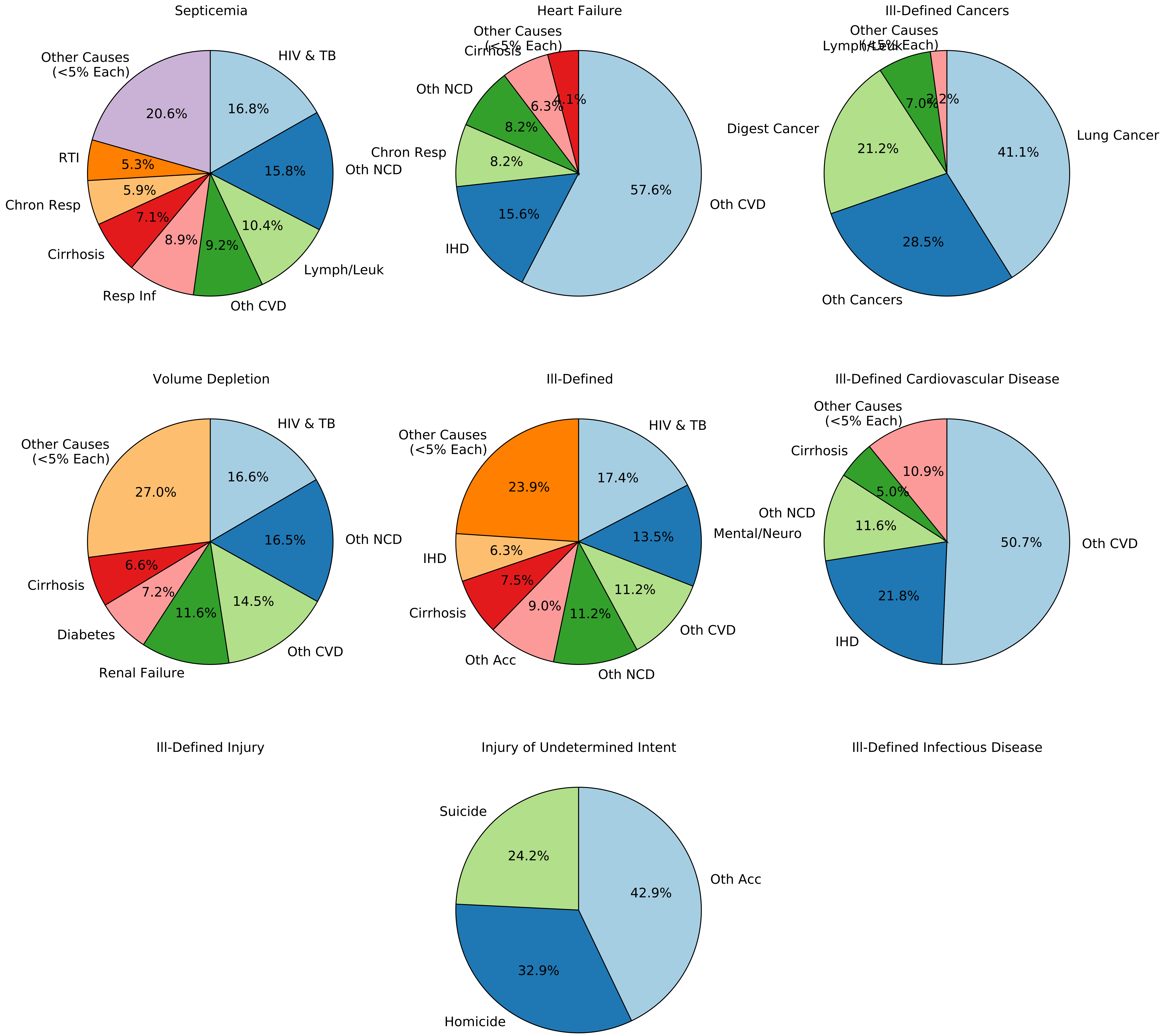

ICD 9
Male, Age 40

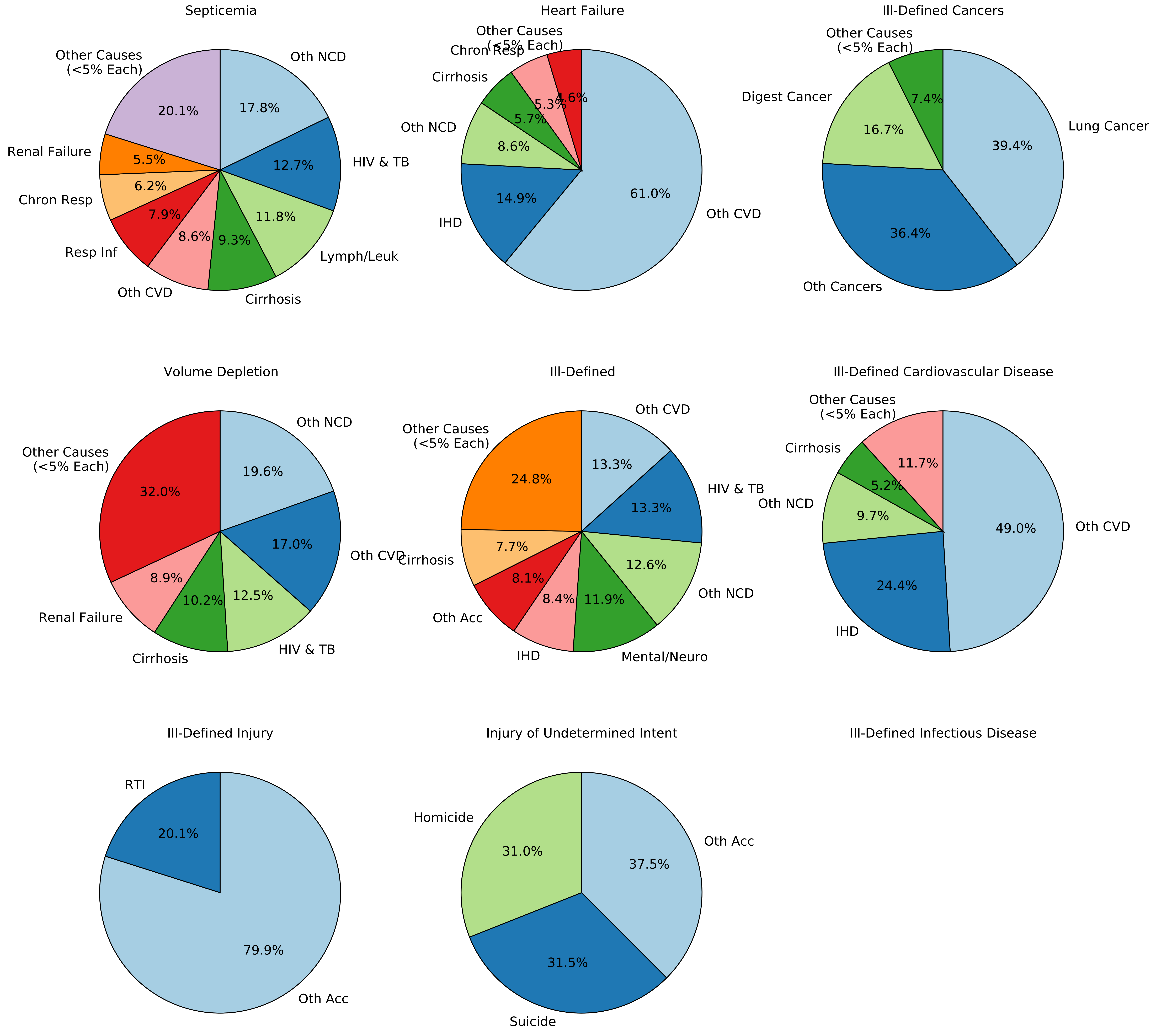

ICD 9
Male, Age 45

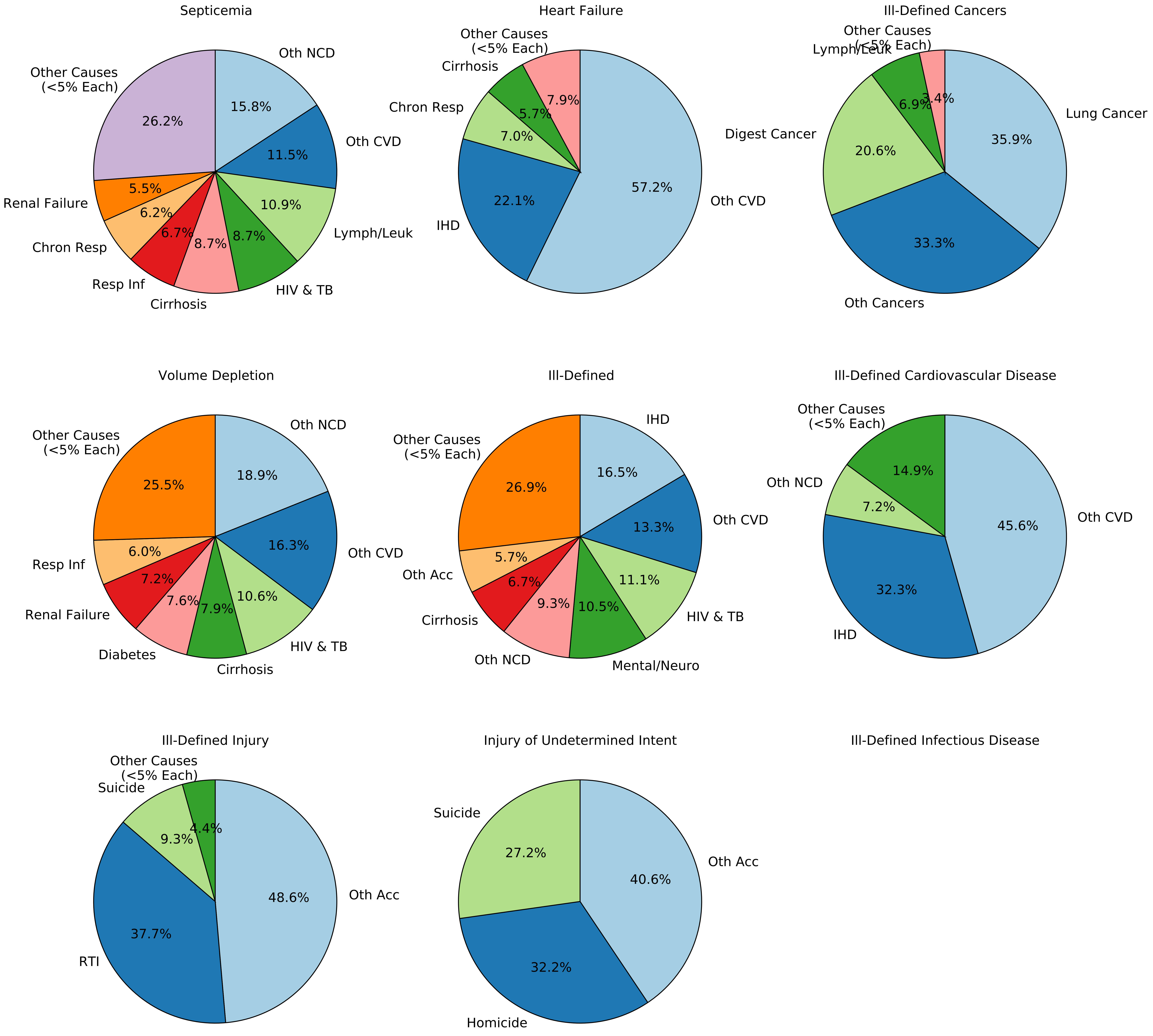

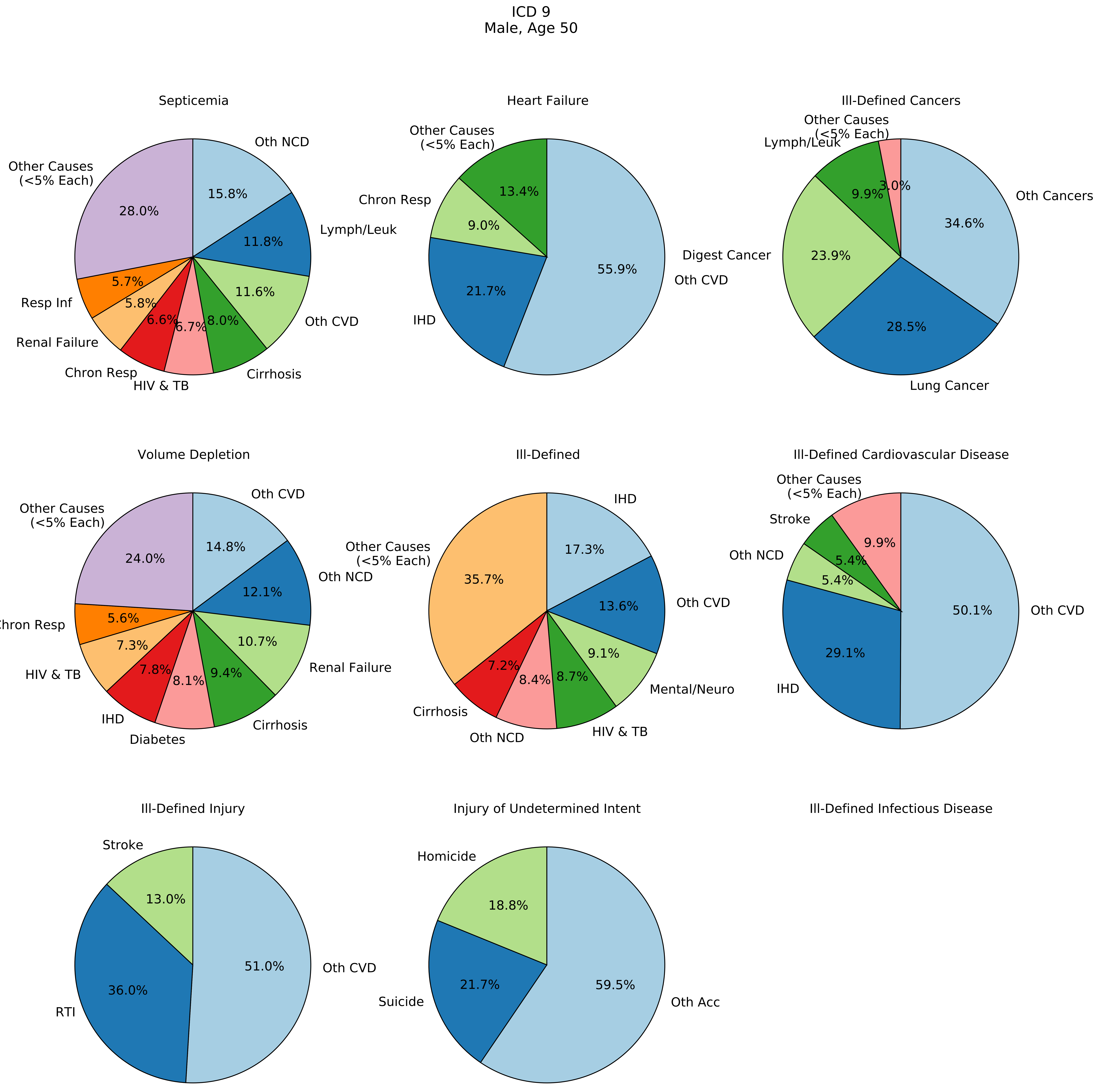
ICD 9
Male, Age 50
Septicemia
Oth NCD 15.8%
Lymph/Leuk 11.8%
Oth CVD 11.6%
Cirrhosis 8.0%
HIV & TB 6.7%
Chron Resp 6.6%
Renal Failure 5.8%
Resp Inf 5.7%
Other Causes (<5% Each) 28.0%
Heart Failure
Oth CVD 55.9%
IHD 21.7%
Chron Resp 9.0%
Other Causes (<5% Each) 13.4%
Ill-Defined Cancers
Oth Cancers 34.6%
Lung Cancer 28.5%
Digest Cancer 23.9%
Lymph/Leuk 9.9%
Other Causes (<5% Each) 3.0%
Volume Depletion
Oth CVD 14.8%
Oth NCD 12.1%
Renal Failure 10.7%
Cirrhosis 9.4%
Diabetes 8.1%
IHD 7.8%
HIV & TB 7.3%
Chron Resp 5.6%
Other Causes (<5% Each) 24.0%
Ill-Defined
IHD 17.3%
Oth CVD 13.6%
Mental/Neuro 9.1%
HIV & TB 8.7%
Oth NCD 8.4%
Cirrhosis 7.2%
Other Causes (<5% Each) 35.7%
Ill-Defined Cardiovascular Disease
Oth CVD 50.1%
IHD 29.1%
Oth NCD 5.4%
Stroke 5.4%
Other Causes (<5% Each) 9.9%
Ill-Defined Injury
Oth CVD 51.0%
RTI 36.0%
Stroke 13.0%
Injury of Undetermined Intent
Oth Acc 59.5%
Suicide 21.7%
Homicide 18.8%
Ill-Defined Infectious Disease

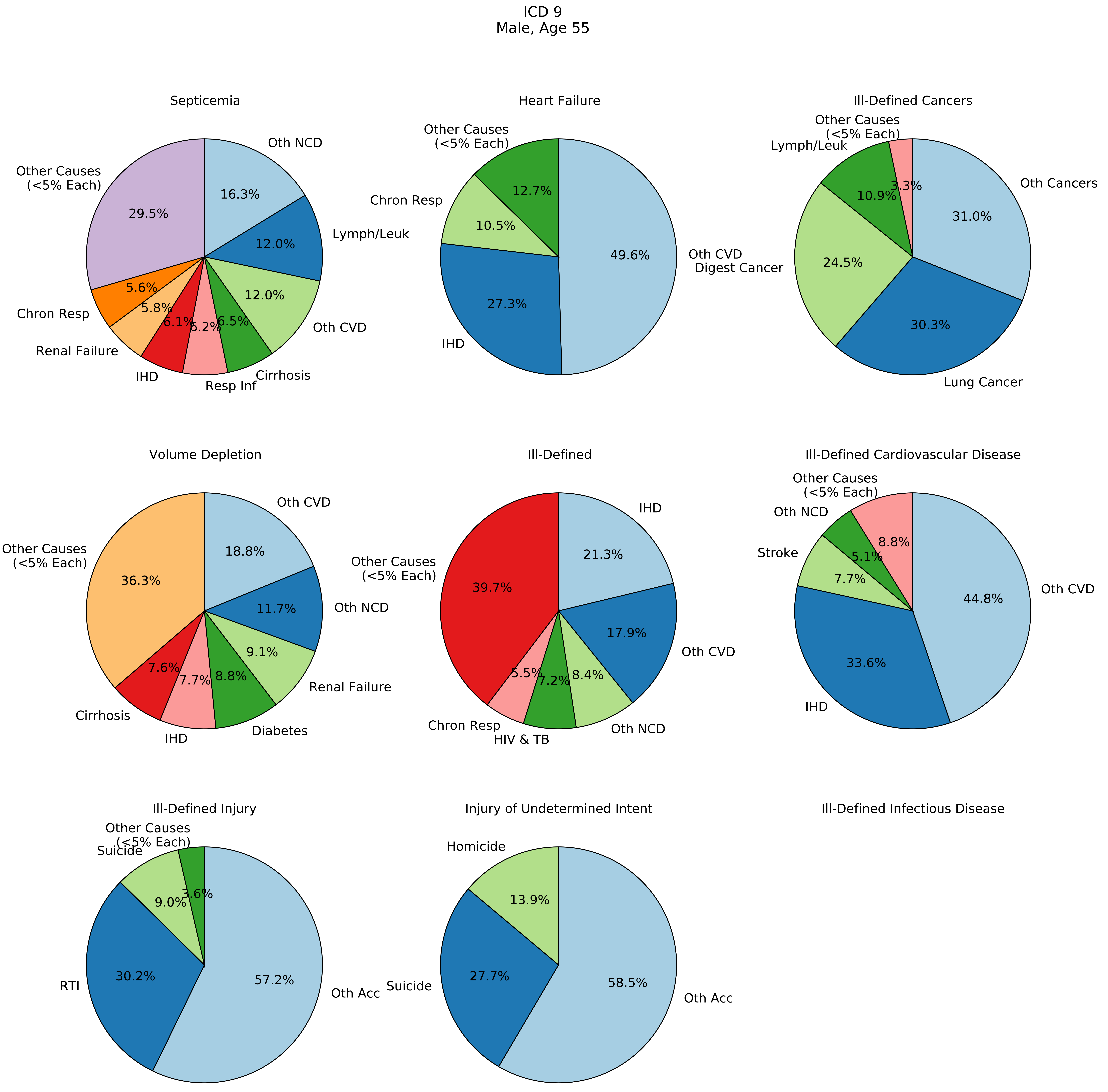
ICD 9
Male, Age 55
Septicemia
Oth NCD 16.3%
Lymph/Leuk 12.0%
Oth CVD 12.0%
Cirrhosis 6.5%
Resp Inf 6.2%
IHD 6.1%
Renal Failure 5.8%
Chron Resp 5.6%
Other Causes (<5% Each) 29.5%
Heart Failure
Oth CVD 49.6%
IHD 27.3%
Chron Resp 10.5%
Other Causes (<5% Each) 12.7%
Ill-Defined Cancers
Oth Cancers 31.0%
Lung Cancer 30.3%
Digest Cancer 24.5%
Lymph/Leuk 10.9%
Other Causes (<5% Each) 3.3%
Volume Depletion
Oth CVD 18.8%
Oth NCD 11.7%
Renal Failure 9.1%
Diabetes 8.8%
IHD 7.7%
Cirrhosis 7.6%
Other Causes (<5% Each) 36.3%
Ill-Defined
IHD 21.3%
Oth CVD 17.9%
Oth NCD 8.4%
HIV & TB 7.2%
Chron Resp 5.5%
Other Causes (<5% Each) 39.7%
Ill-Defined Cardiovascular Disease
Oth CVD 44.8%
IHD 33.6%
Stroke 7.7%
Oth NCD 5.1%
Other Causes (<5% Each) 8.8%
Ill-Defined Injury
Oth Acc 57.2%
RTI 30.2%
Suicide 9.0%
Other Causes (<5% Each) 3.6%
Injury of Undetermined Intent
Oth Acc 58.5%
Suicide 27.7%
Homicide 13.9%
Ill-Defined Infectious Disease

ICD 9
Male, Age 60

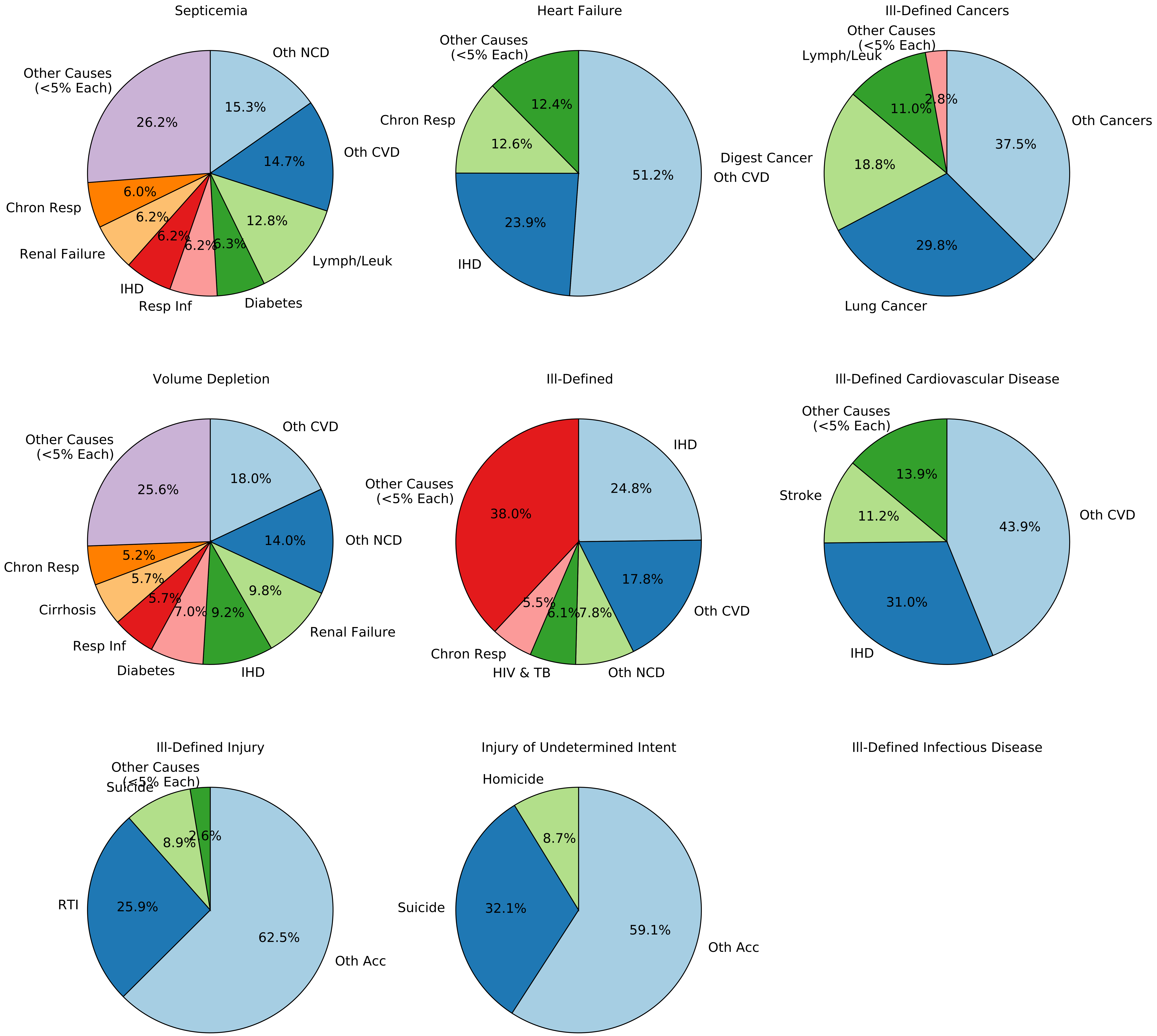

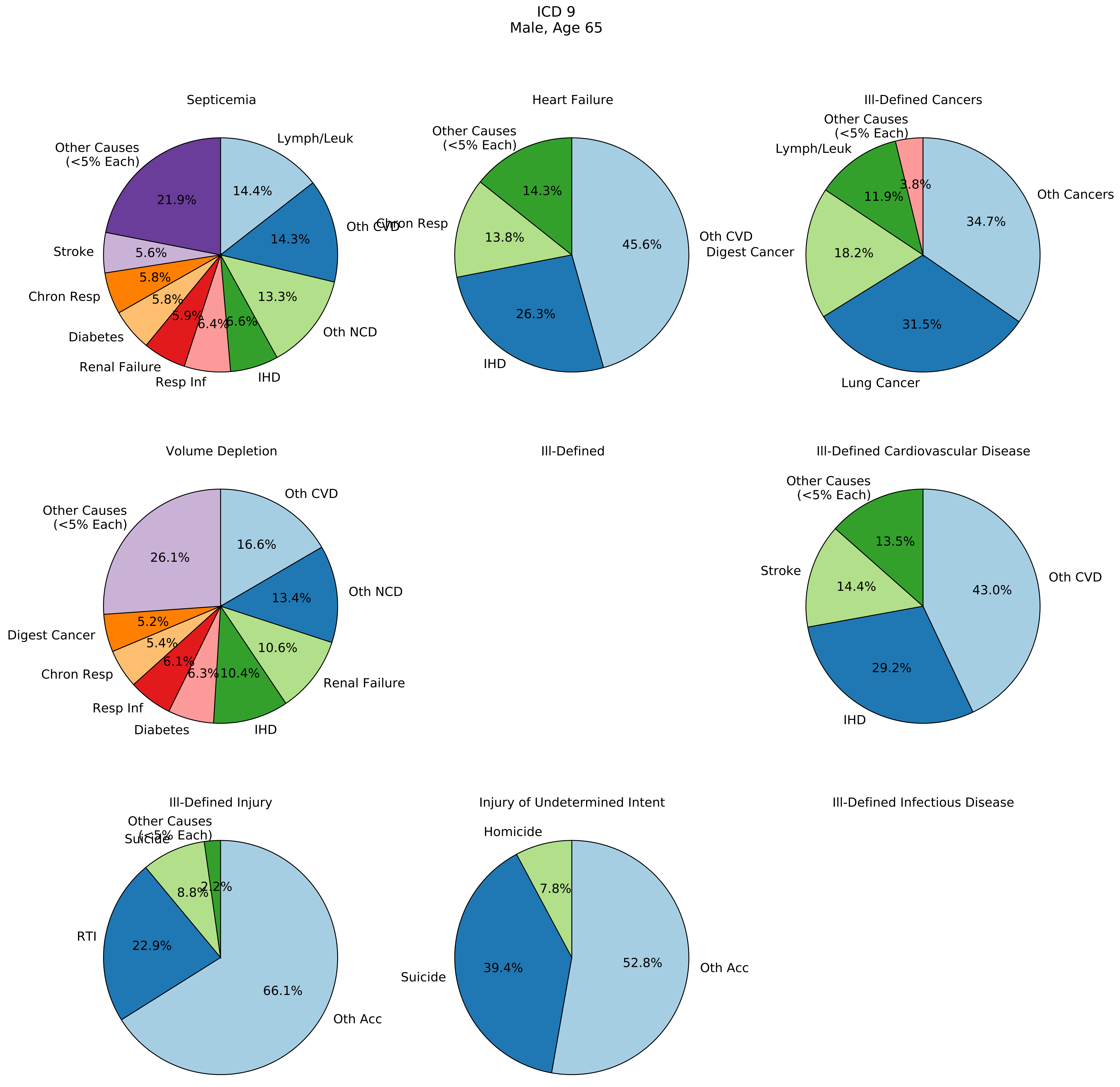

ICD 9
Male, Age 65
Septicemia
Lymph/Leuk
14.4%
Oth CVD
14.3%
Oth NCD
13.3%
IHD
6.6%
Resp Inf
6.4%
Renal Failure
5.9%
Diabetes
5.8%
Chron Resp
5.8%
Stroke
5.6%
Other Causes
(<5% Each)
21.9%
Heart Failure
Oth CVD
45.6%
IHD
26.3%
Chron Resp
13.8%
Other Causes
(<5% Each)
14.3%
Ill-Defined Cancers
Oth Cancers
34.7%
Lung Cancer
31.5%
Digest Cancer
18.2%
Lymph/Leuk
11.9%
Other Causes
(<5% Each)
3.8%
Volume Depletion
Oth CVD
16.6%
Oth NCD
13.4%
Renal Failure
10.6%
IHD
10.4%
Diabetes
6.3%
Resp Inf
6.1%
Chron Resp
5.4%
Digest Cancer
5.2%
Other Causes
(<5% Each)
26.1%
Ill-Defined
Ill-Defined Cardiovascular Disease
Oth CVD
43.0%
IHD
29.2%
Stroke
14.4%
Other Causes
(<5% Each)
13.5%
Ill-Defined Injury
Oth Acc
66.1%
RTI
22.9%
Suicide
8.8%
Other Causes
(<5% Each)
2.2%
Injury of Undetermined Intent
Oth Acc
52.8%
Suicide
39.4%
Homicide
7.8%
Ill-Defined Infectious Disease

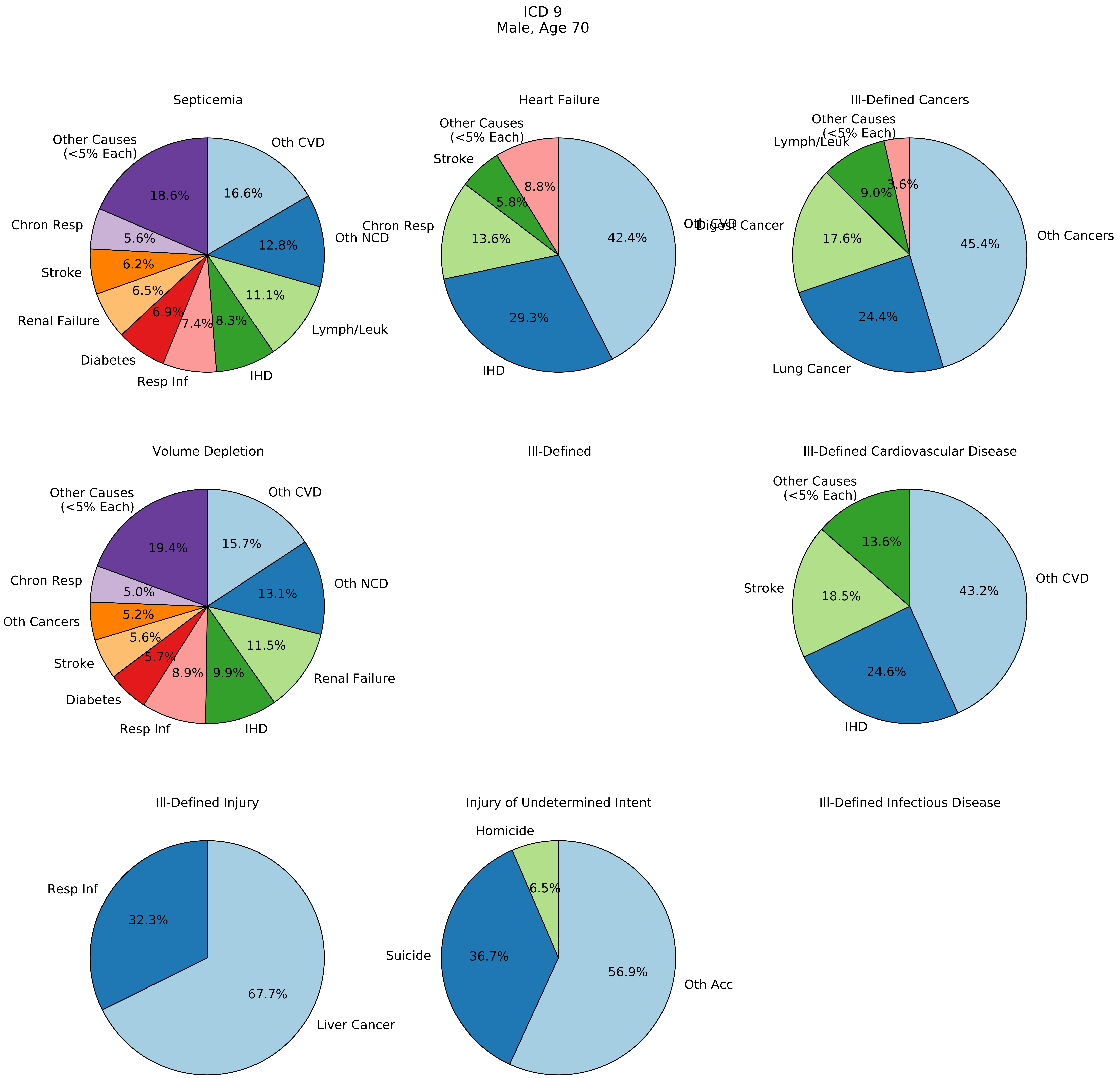
ICD 9
Male, Age 70
Septicemia
Other Causes (<5% Each) 18.6%
Oth CVD 16.6%
Oth NCD 12.8%
Lymph/Leuk 11.1%
IHD 8.3%
Resp Inf 7.4%
Diabetes 6.9%
Renal Failure 6.5%
Stroke 6.2%
Chron Resp 5.6%
Heart Failure
Oth CVD 42.4%
IHD 29.3%
Chron Resp 13.6%
Stroke 5.8%
Other Causes (<5% Each) 8.8%
Ill-Defined Cancers
Oth Cancers 45.4%
Lung Cancer 24.4%
Digest Cancer 17.6%
Lymph/Leuk 9.0%
Other Causes (<5% Each) 3.6%
Volume Depletion
Other Causes (<5% Each) 19.4%
Oth CVD 15.7%
Oth NCD 13.1%
Renal Failure 11.5%
IHD 9.9%
Resp Inf 8.9%
Diabetes 5.7%
Stroke 5.6%
Oth Cancers 5.2%
Chron Resp 5.0%
Ill-Defined
Ill-Defined Cardiovascular Disease
Oth CVD 43.2%
IHD 24.6%
Stroke 18.5%
Other Causes (<5% Each) 13.6%
Ill-Defined Injury
Resp Inf 32.3%
Liver Cancer 67.7%
Injury of Undetermined Intent
Oth Acc 56.9%
Suicide 36.7%
Homicide 6.5%
Ill-Defined Infectious Disease

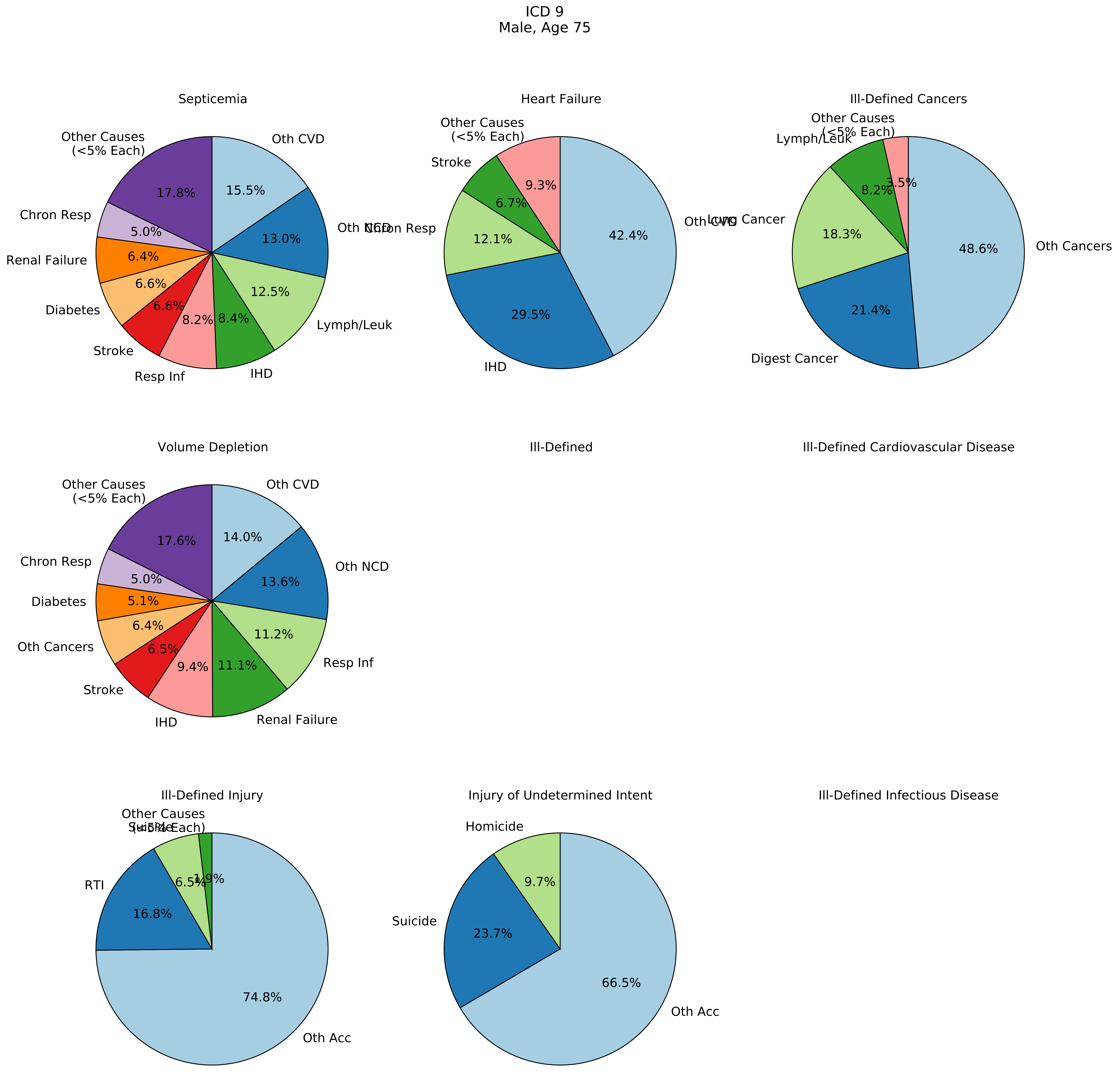
ICD 9
Male, Age 75
Septicemia
Other Causes
(<5% Each)
Oth CVD
Oth NCD
Chron Resp
Renal Failure
Diabetes
Stroke
Resp Inf
IHD
Lymph/Leuk
17.8%
15.5%
13.0%
5.0%
6.4%
6.6%
6.6%
8.2%
8.4%
12.5%
Heart Failure
Other Causes
(<5% Each)
Stroke
Chron Resp
IHD
Oth CVD
9.3%
6.7%
12.1%
42.4%
29.5%
Ill-Defined Cancers
Other Causes
(<5% Each)
Lymph/Leuk
Lung Cancer
Oth Cancers
Digest Cancer
8.2%
3.5%
18.3%
48.6%
21.4%
Volume Depletion
Other Causes
(<5% Each)
Oth CVD
Oth NCD
Chron Resp
Diabetes
Oth Cancers
Stroke
IHD
Renal Failure
Resp Inf
17.6%
14.0%
13.6%
5.0%
5.1%
6.4%
6.5%
9.4%
11.1%
11.2%
Ill-Defined
Ill-Defined Cardiovascular Disease
Ill-Defined Injury
Other Causes
(<5% Each)
Suicide
RTI
Oth Acc
6.5%
1.9%
16.8%
74.8%
Injury of Undetermined Intent
Homicide
Suicide
Oth Acc
9.7%
23.7%
66.5%
Ill-Defined Infectious Disease

ICD 9
Male, Age 80

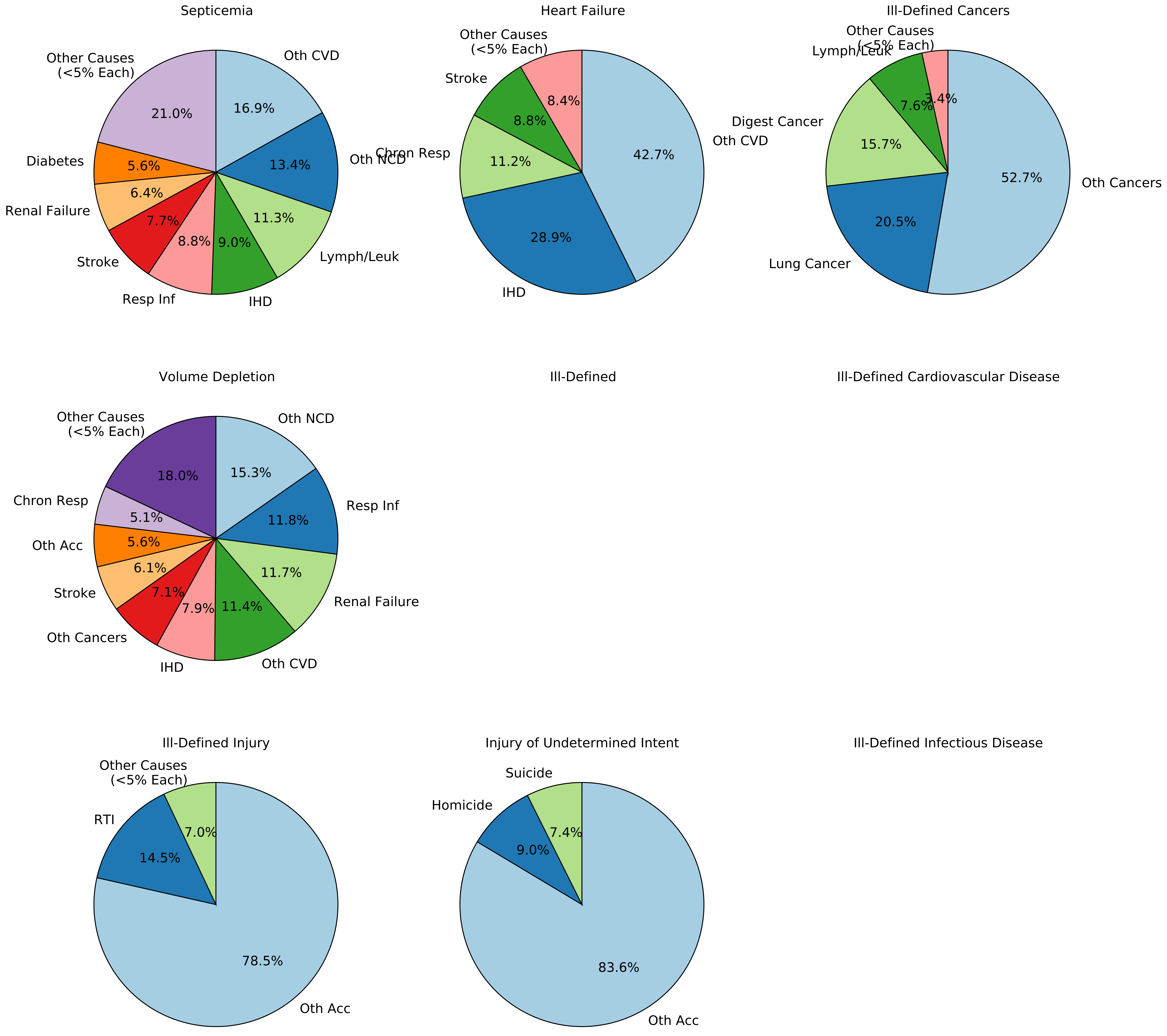

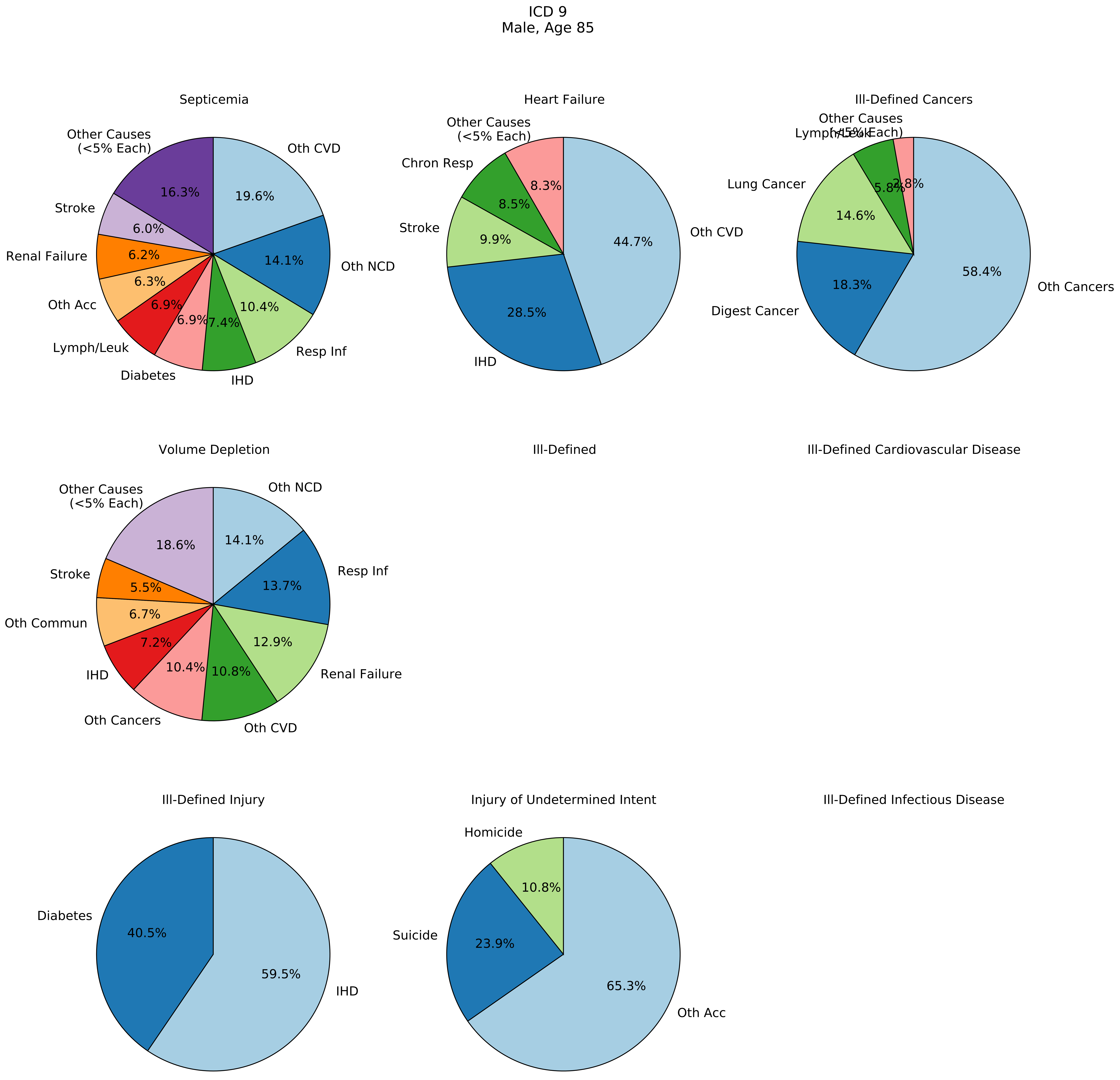
ICD 9
Male, Age 85
Septicemia
Other Causes
(<5% Each)
16.3%
Oth CVD
19.6%
Stroke
6.0%
Renal Failure
6.2%
Oth Acc
6.3%
Lymph/Leuk
6.9%
Diabetes
6.9%
IHD
7.4%
Resp Inf
10.4%
Oth NCD
14.1%
Heart Failure
Other Causes
(<5% Each)
8.3%
Chron Resp
8.5%
Stroke
9.9%
IHD
28.5%
Oth CVD
44.7%
Ill-Defined Cancers
Other Causes
Lymph/Leuk
5.8%
Lung Cancer
14.6%
Digest Cancer
18.3%
Oth Cancers
58.4%
Volume Depletion
Other Causes
(<5% Each)
18.6%
Oth NCD
14.1%
Resp Inf
13.7%
Renal Failure
12.9%
Oth CVD
10.8%
Oth Cancers
10.4%
IHD
7.2%
Oth Commun
6.7%
Stroke
5.5%
Ill-Defined
Ill-Defined Cardiovascular Disease
Ill-Defined Injury
Diabetes
40.5%
IHD
59.5%
Injury of Undetermined Intent
Homicide
10.8%
Suicide
23.9%
Oth Acc
65.3%
Ill-Defined Infectious Disease

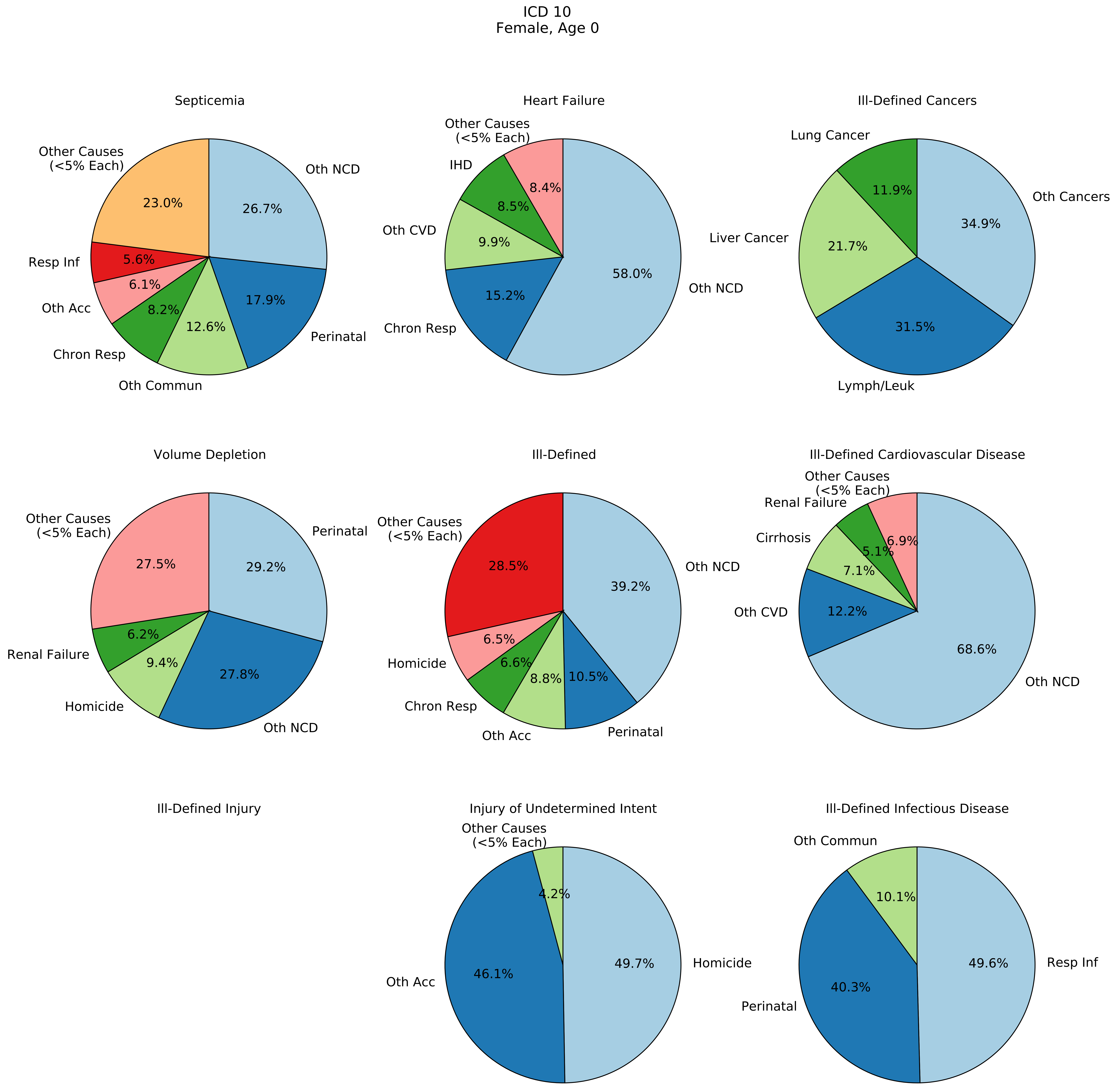
ICD 10
Female, Age 0
Septicemia
Oth NCD 26.7%
Perinatal 17.9%
Oth Commun 12.6%
Chron Resp 8.2%
Oth Acc 6.1%
Resp Inf 5.6%
Other Causes (<5% Each) 23.0%
Heart Failure
Oth NCD 58.0%
Chron Resp 15.2%
Oth CVD 9.9%
IHD 8.5%
Other Causes (<5% Each) 8.4%
Ill-Defined Cancers
Oth Cancers 34.9%
Lymph/Leuk 31.5%
Liver Cancer 21.7%
Lung Cancer 11.9%
Volume Depletion
Perinatal 29.2%
Oth NCD 27.8%
Homicide 9.4%
Renal Failure 6.2%
Other Causes (<5% Each) 27.5%
Ill-Defined
Oth NCD 39.2%
Perinatal 10.5%
Oth Acc 8.8%
Chron Resp 6.6%
Homicide 6.5%
Other Causes (<5% Each) 28.5%
Ill-Defined Cardiovascular Disease
Oth NCD 68.6%
Oth CVD 12.2%
Cirrhosis 7.1%
Renal Failure 5.1%
Other Causes (<5% Each) 6.9%
Ill-Defined Injury
Injury of Undetermined Intent
Homicide 49.7%
Oth Acc 46.1%
Other Causes (<5% Each) 4.2%
Ill-Defined Infectious Disease
Resp Inf 49.6%
Perinatal 40.3%
Oth Commun 10.1%

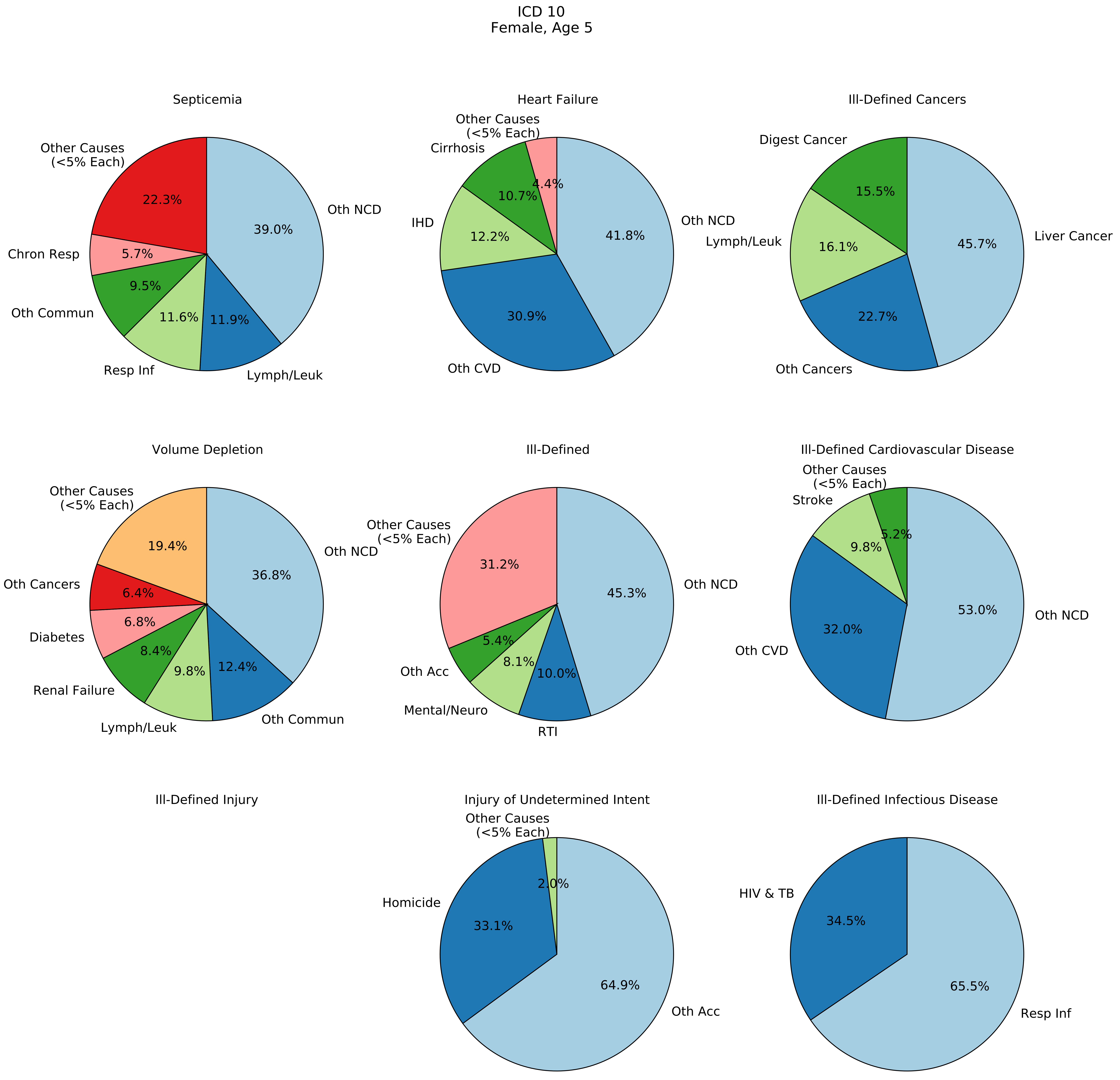
ICD 10
Female, Age 5
Septicemia
Oth NCD 39.0%
Lymph/Leuk 11.9%
Resp Inf 11.6%
Oth Commun 9.5%
Chron Resp 5.7%
Other Causes (<5% Each) 22.3%
Heart Failure
Oth NCD 41.8%
Oth CVD 30.9%
IHD 12.2%
Cirrhosis 10.7%
Other Causes (<5% Each) 4.4%
Ill-Defined Cancers
Liver Cancer 45.7%
Oth Cancers 22.7%
Lymph/Leuk 16.1%
Digest Cancer 15.5%
Volume Depletion
Oth NCD 36.8%
Oth Commun 12.4%
Lymph/Leuk 9.8%
Renal Failure 8.4%
Diabetes 6.8%
Oth Cancers 6.4%
Other Causes (<5% Each) 19.4%
Ill-Defined
Oth NCD 45.3%
RTI 10.0%
Mental/Neuro 8.1%
Oth Acc 5.4%
Other Causes (<5% Each) 31.2%
Ill-Defined Cardiovascular Disease
Oth NCD 53.0%
Oth CVD 32.0%
Stroke 9.8%
Other Causes (<5% Each) 5.2%
Ill-Defined Injury
Injury of Undetermined Intent
Oth Acc 64.9%
Homicide 33.1%
Other Causes (<5% Each) 2.0%
Ill-Defined Infectious Disease
Resp Inf 65.5%
HIV & TB 34.5%

ICD 10
Female, Age 10

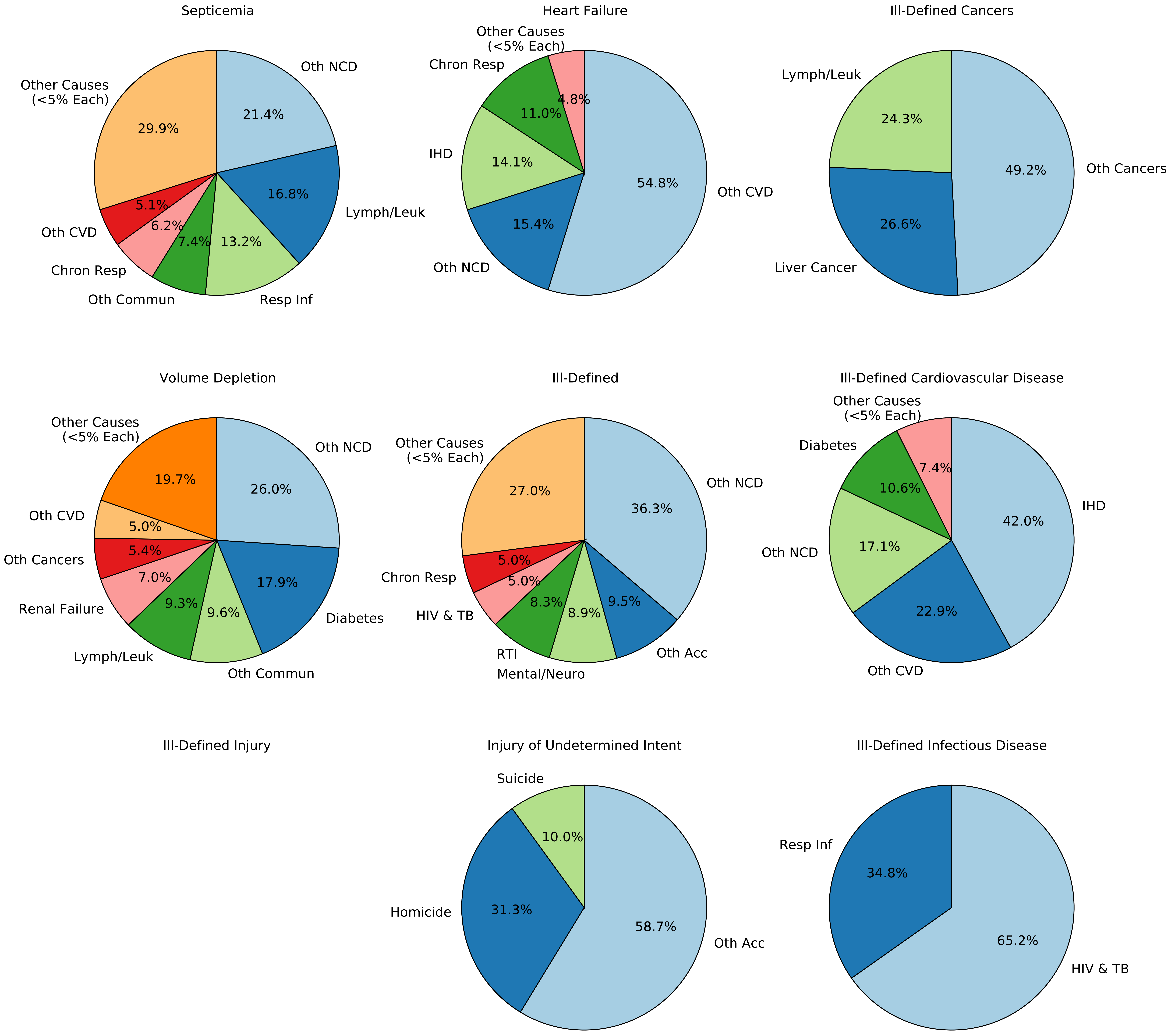

ICD 10
Female, Age 15

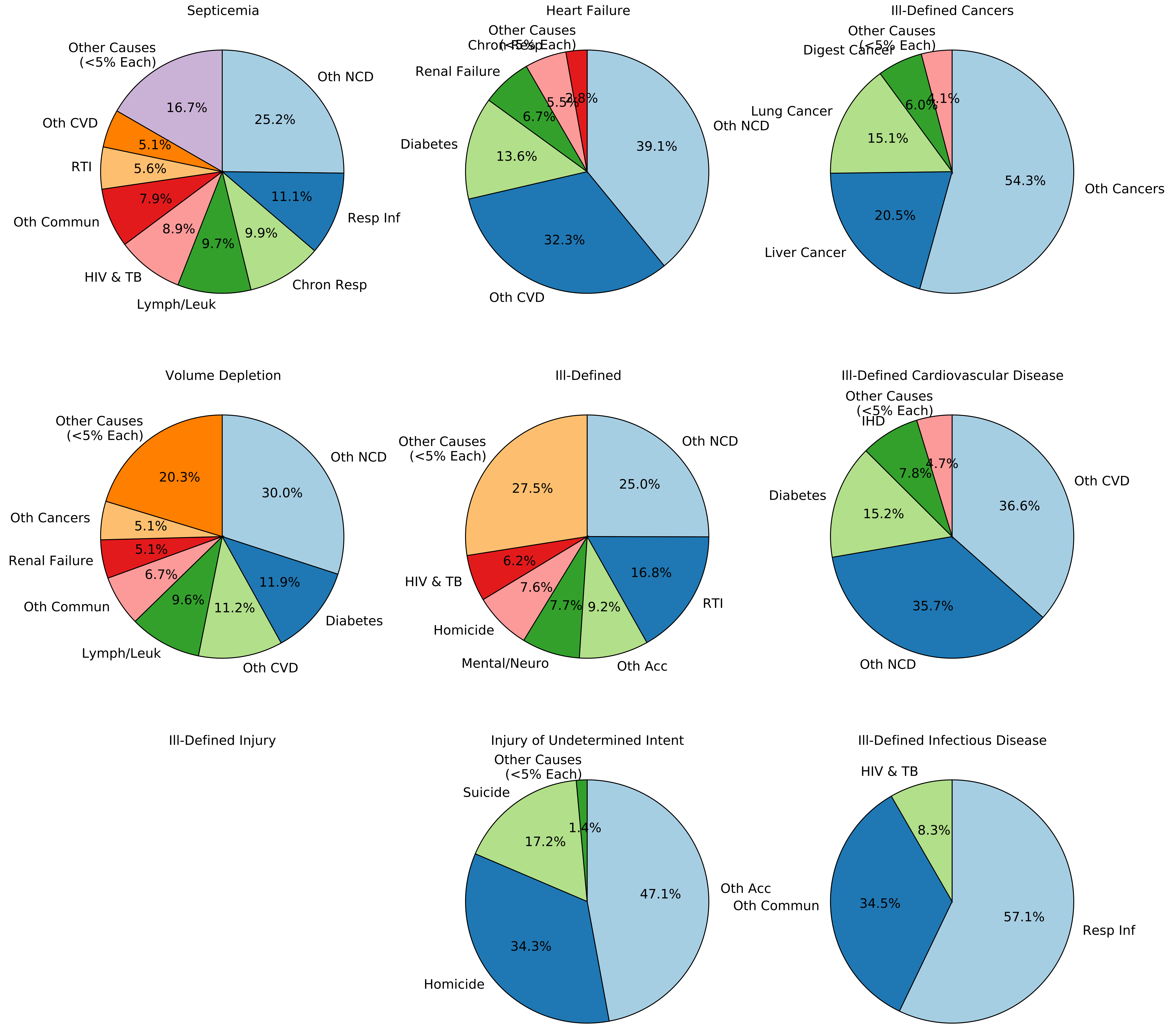

ICD 10
Female, Age 20

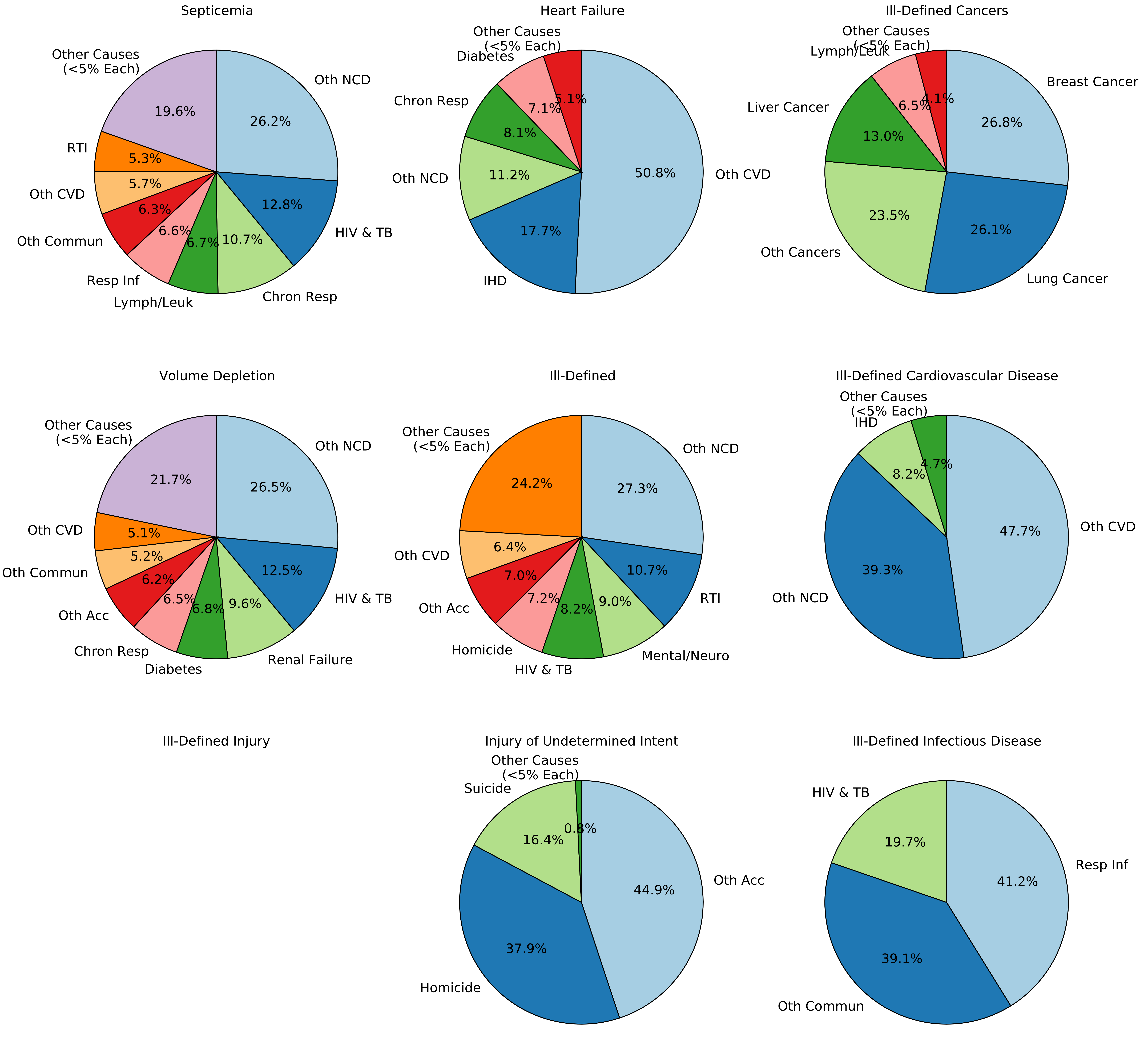

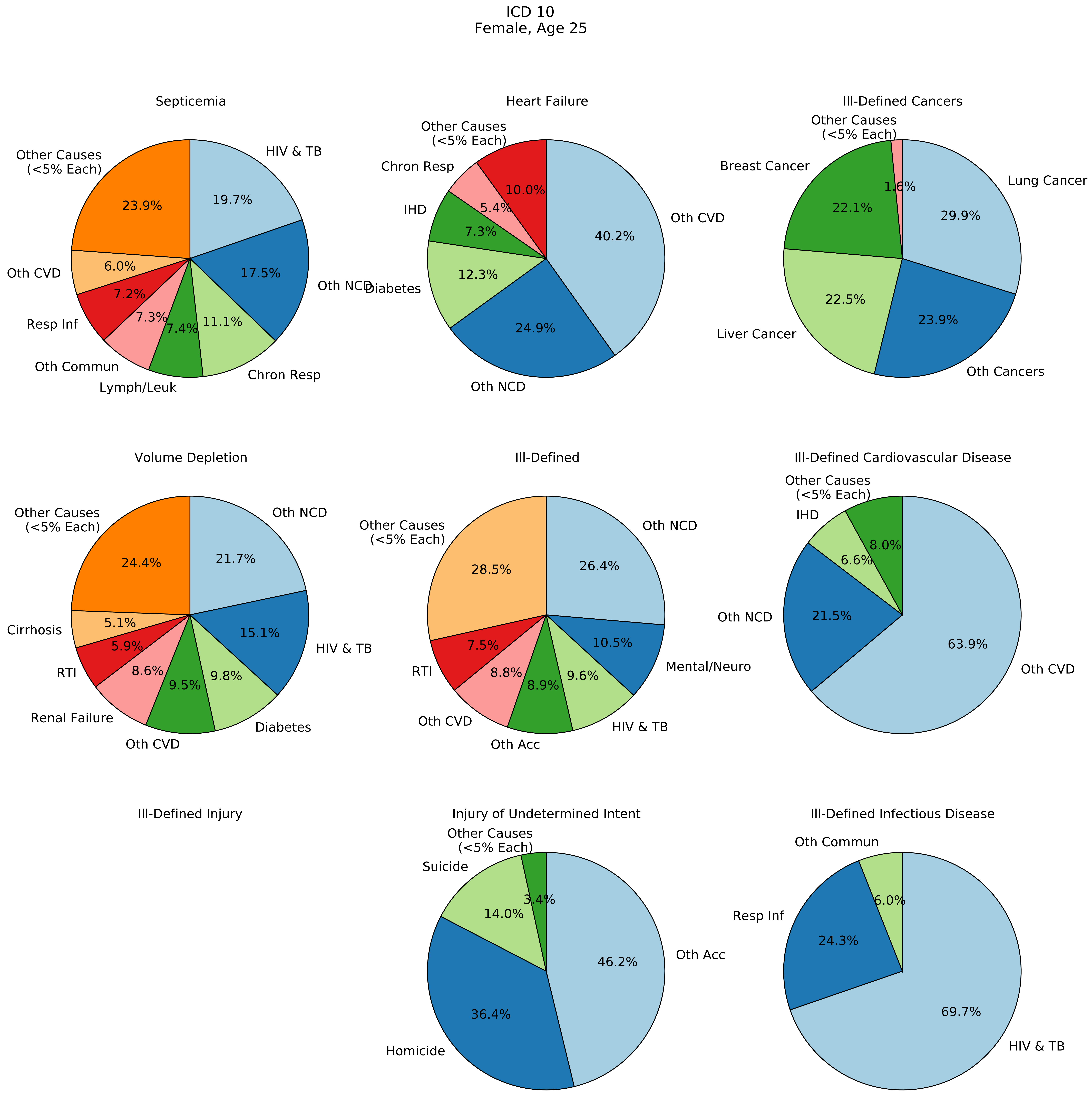
ICD 10
Female, Age 25
Septicemia
HIV & TB 19.7%
Oth NCD 17.5%
Chron Resp 11.1%
Lymph/Leuk 7.4%
Oth Commun 7.3%
Resp Inf 7.2%
Oth CVD 6.0%
Other Causes (<5% Each) 23.9%
Heart Failure
Oth CVD 40.2%
Oth NCD 24.9%
Diabetes 12.3%
IHD 7.3%
Chron Resp 5.4%
Other Causes (<5% Each) 10.0%
Ill-Defined Cancers
Lung Cancer 29.9%
Oth Cancers 23.9%
Liver Cancer 22.5%
Breast Cancer 22.1%
Other Causes (<5% Each) 1.6%
Volume Depletion
Oth NCD 21.7%
HIV & TB 15.1%
Diabetes 9.8%
Oth CVD 9.5%
Renal Failure 8.6%
RTI 5.9%
Cirrhosis 5.1%
Other Causes (<5% Each) 24.4%
Ill-Defined
Oth NCD 26.4%
Mental/Neuro 10.5%
HIV & TB 9.6%
Oth Acc 8.9%
Oth CVD 8.8%
RTI 7.5%
Other Causes (<5% Each) 28.5%
Ill-Defined Cardiovascular Disease
Oth CVD 63.9%
Oth NCD 21.5%
IHD 6.6%
Other Causes (<5% Each) 8.0%
Ill-Defined Injury
Injury of Undetermined Intent
Oth Acc 46.2%
Homicide 36.4%
Suicide 14.0%
Other Causes (<5% Each) 3.4%
Ill-Defined Infectious Disease
HIV & TB 69.7%
Resp Inf 24.3%
Oth Commun 6.0%

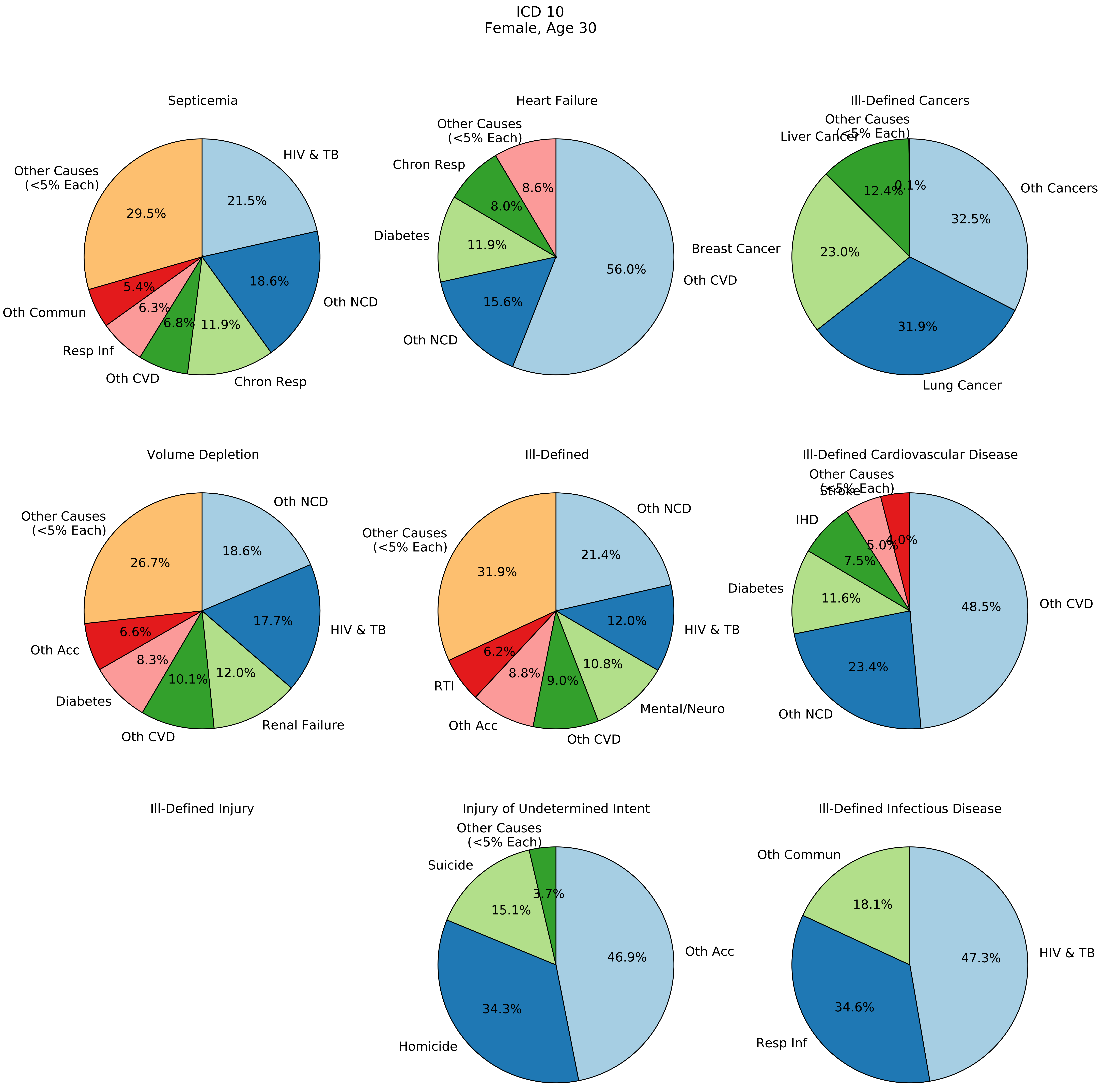
ICD 10
Female, Age 30
Septicemia
HIV & TB 21.5%
Oth NCD 18.6%
Chron Resp 11.9%
Oth CVD 6.8%
Resp Inf 6.3%
Oth Commun 5.4%
Other Causes (<5% Each) 29.5%
Heart Failure
Oth CVD 56.0%
Oth NCD 15.6%
Diabetes 11.9%
Chron Resp 8.0%
Other Causes (<5% Each) 8.6%
Ill-Defined Cancers
Oth Cancers 32.5%
Lung Cancer 31.9%
Breast Cancer 23.0%
Liver Cancer 12.4%
Other Causes (<5% Each) 0.1%
Volume Depletion
Oth NCD 18.6%
HIV & TB 17.7%
Renal Failure 12.0%
Oth CVD 10.1%
Diabetes 8.3%
Oth Acc 6.6%
Other Causes (<5% Each) 26.7%
Ill-Defined
Oth NCD 21.4%
HIV & TB 12.0%
Mental/Neuro 10.8%
Oth CVD 9.0%
Oth Acc 8.8%
RTI 6.2%
Other Causes (<5% Each) 31.9%
Ill-Defined Cardiovascular Disease
Oth CVD 48.5%
Oth NCD 23.4%
Diabetes 11.6%
IHD 7.5%
Stroke 5.0%
Other Causes (<5% Each) 4.0%
Ill-Defined Injury
Injury of Undetermined Intent
Oth Acc 46.9%
Homicide 34.3%
Suicide 15.1%
Other Causes (<5% Each) 3.7%
Ill-Defined Infectious Disease
HIV & TB 47.3%
Resp Inf 34.6%
Oth Commun 18.1%

ICD 10
Female, Age 35

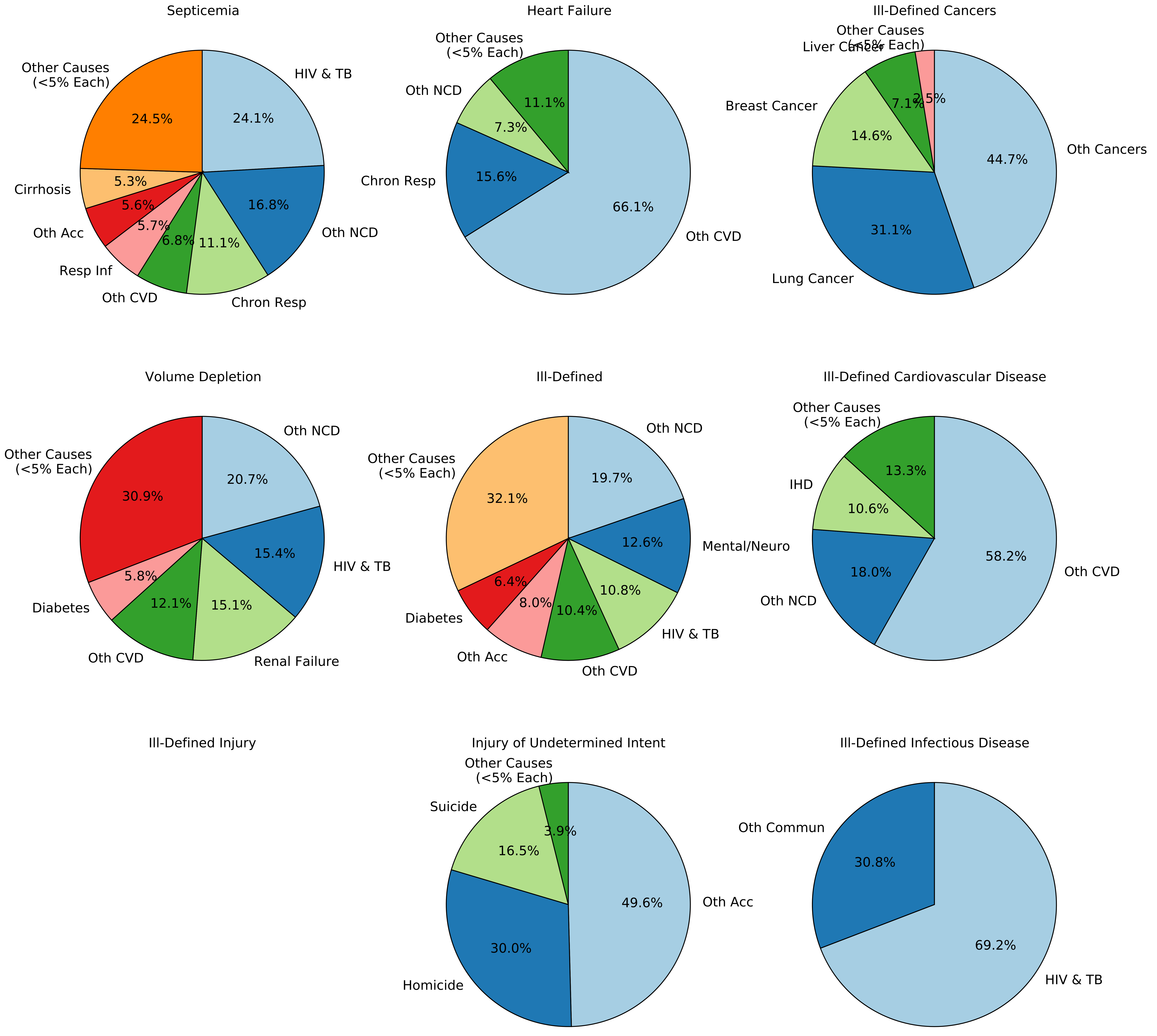

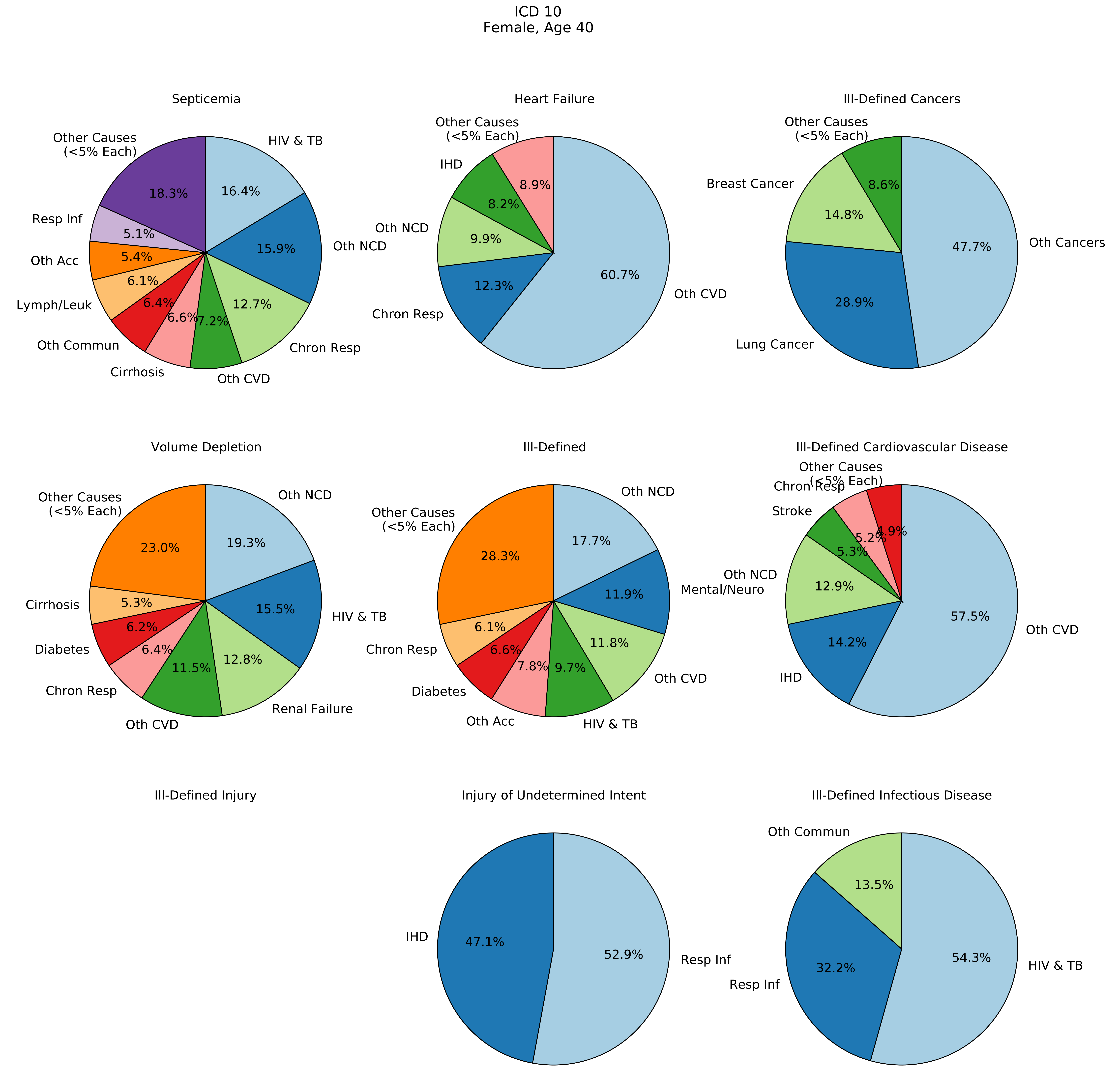
ICD 10
Female, Age 40
Septicemia
HIV & TB
16.4%
Oth NCD
15.9%
Chron Resp
12.7%
Oth CVD
7.2%
Cirrhosis
6.6%
Oth Commun
6.4%
Lymph/Leuk
6.1%
Oth Acc
5.4%
Resp Inf
5.1%
Other Causes
(<5% Each)
18.3%
Heart Failure
Oth CVD
60.7%
Chron Resp
12.3%
Oth NCD
9.9%
IHD
8.2%
Other Causes
(<5% Each)
8.9%
Ill-Defined Cancers
Oth Cancers
47.7%
Lung Cancer
28.9%
Breast Cancer
14.8%
Other Causes
(<5% Each)
8.6%
Volume Depletion
Oth NCD
19.3%
HIV & TB
15.5%
Renal Failure
12.8%
Oth CVD
11.5%
Chron Resp
6.4%
Diabetes
6.2%
Cirrhosis
5.3%
Other Causes
(<5% Each)
23.0%
Ill-Defined
Oth NCD
17.7%
Mental/Neuro
11.9%
Oth CVD
11.8%
HIV & TB
9.7%
Oth Acc
7.8%
Diabetes
6.6%
Chron Resp
6.1%
Other Causes
(<5% Each)
28.3%
Ill-Defined Cardiovascular Disease
Oth CVD
57.5%
IHD
14.2%
Oth NCD
12.9%
Stroke
5.3%
Chron Resp
5.2%
Other Causes
(<5% Each)
4.9%
Ill-Defined Injury
Injury of Undetermined Intent
Resp Inf
52.9%
IHD
47.1%
Ill-Defined Infectious Disease
HIV & TB
54.3%
Resp Inf
32.2%
Oth Commun
13.5%

ICD 10
Female, Age 45

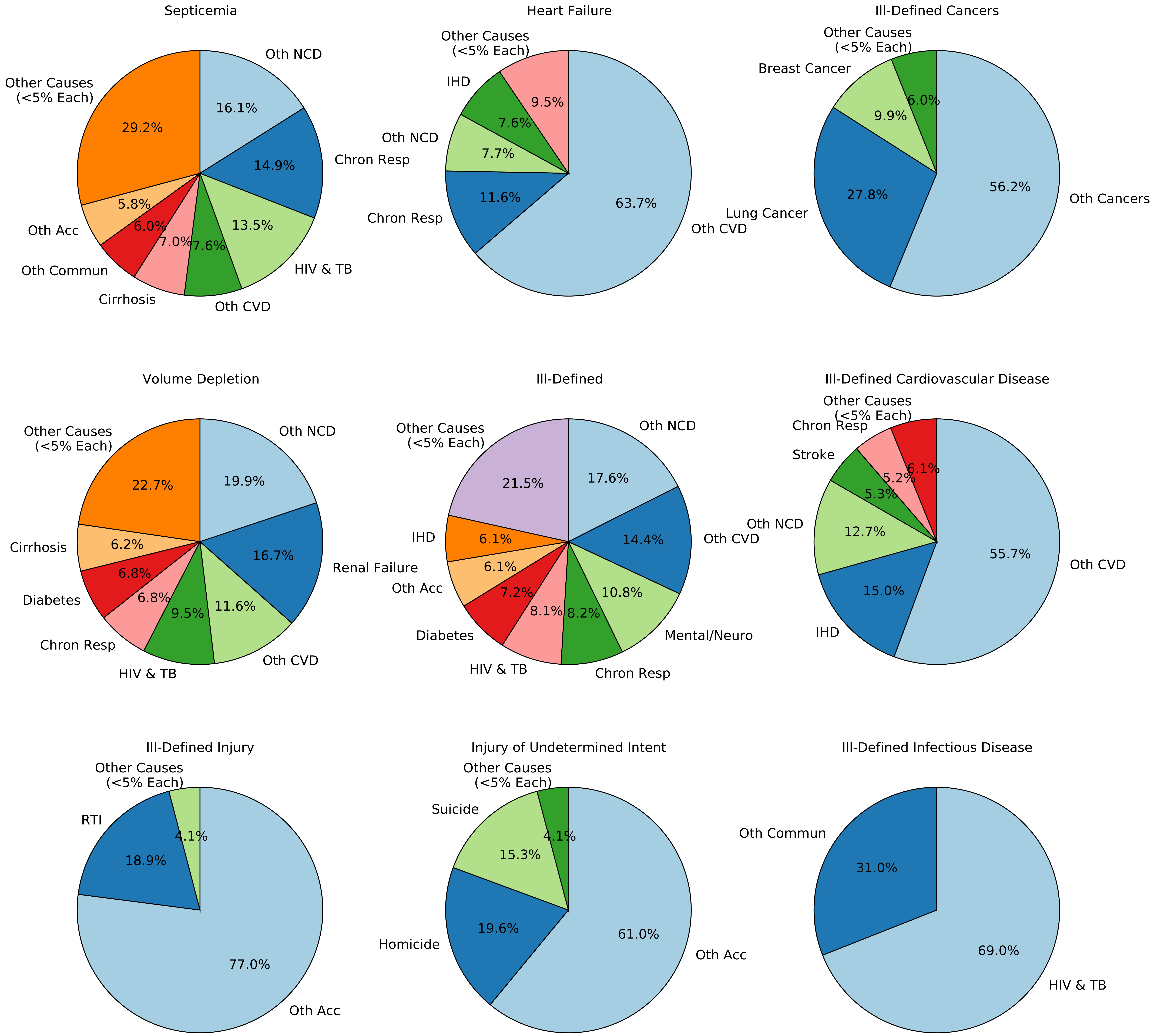

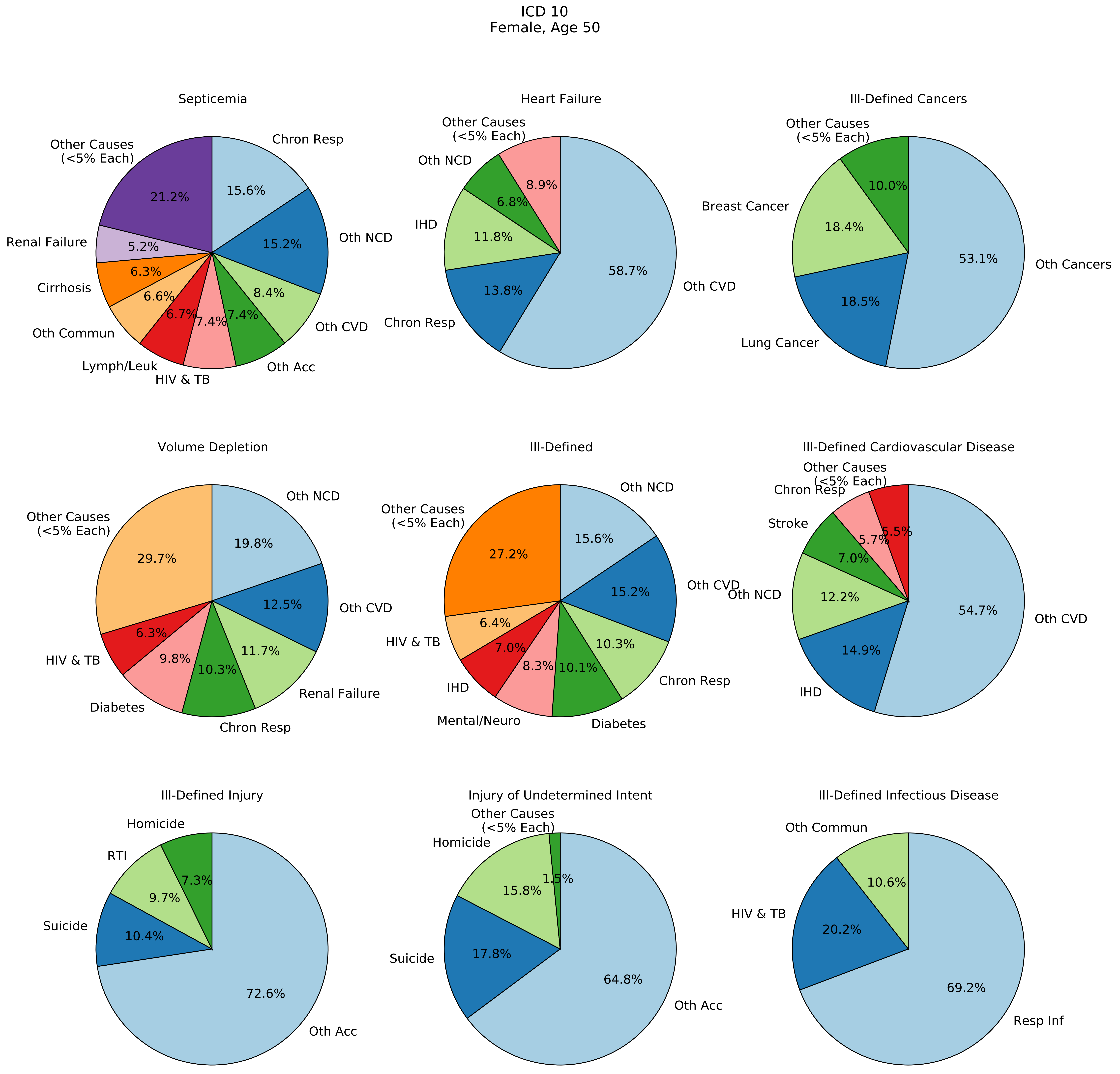
ICD 10
Female, Age 50
Septicemia
Chron Resp 15.6%
Oth NCD 15.2%
Oth CVD 8.4%
Oth Acc 7.4%
HIV & TB 7.4%
Lymph/Leuk 6.7%
Oth Commun 6.6%
Cirrhosis 6.3%
Renal Failure 5.2%
Other Causes (<5% Each) 21.2%
Heart Failure
Oth CVD 58.7%
Chron Resp 13.8%
IHD 11.8%
Oth NCD 6.8%
Other Causes (<5% Each) 8.9%
Ill-Defined Cancers
Oth Cancers 53.1%
Lung Cancer 18.5%
Breast Cancer 18.4%
Other Causes (<5% Each) 10.0%
Volume Depletion
Oth NCD 19.8%
Oth CVD 12.5%
Renal Failure 11.7%
Chron Resp 10.3%
Diabetes 9.8%
HIV & TB 6.3%
Other Causes (<5% Each) 29.7%
Ill-Defined
Oth NCD 15.6%
Oth CVD 15.2%
Chron Resp 10.3%
Diabetes 10.1%
Mental/Neuro 8.3%
IHD 7.0%
HIV & TB 6.4%
Other Causes (<5% Each) 27.2%
Ill-Defined Cardiovascular Disease
Oth CVD 54.7%
IHD 14.9%
Oth NCD 12.2%
Stroke 7.0%
Chron Resp 5.7%
Other Causes (<5% Each) 5.5%
Ill-Defined Injury
Oth Acc 72.6%
Suicide 10.4%
RTI 9.7%
Homicide 7.3%
Injury of Undetermined Intent
Oth Acc 64.8%
Suicide 17.8%
Homicide 15.8%
Other Causes (<5% Each) 1.5%
Ill-Defined Infectious Disease
Resp Inf 69.2%
HIV & TB 20.2%
Oth Commun 10.6%

ICD 10
Female, Age 55

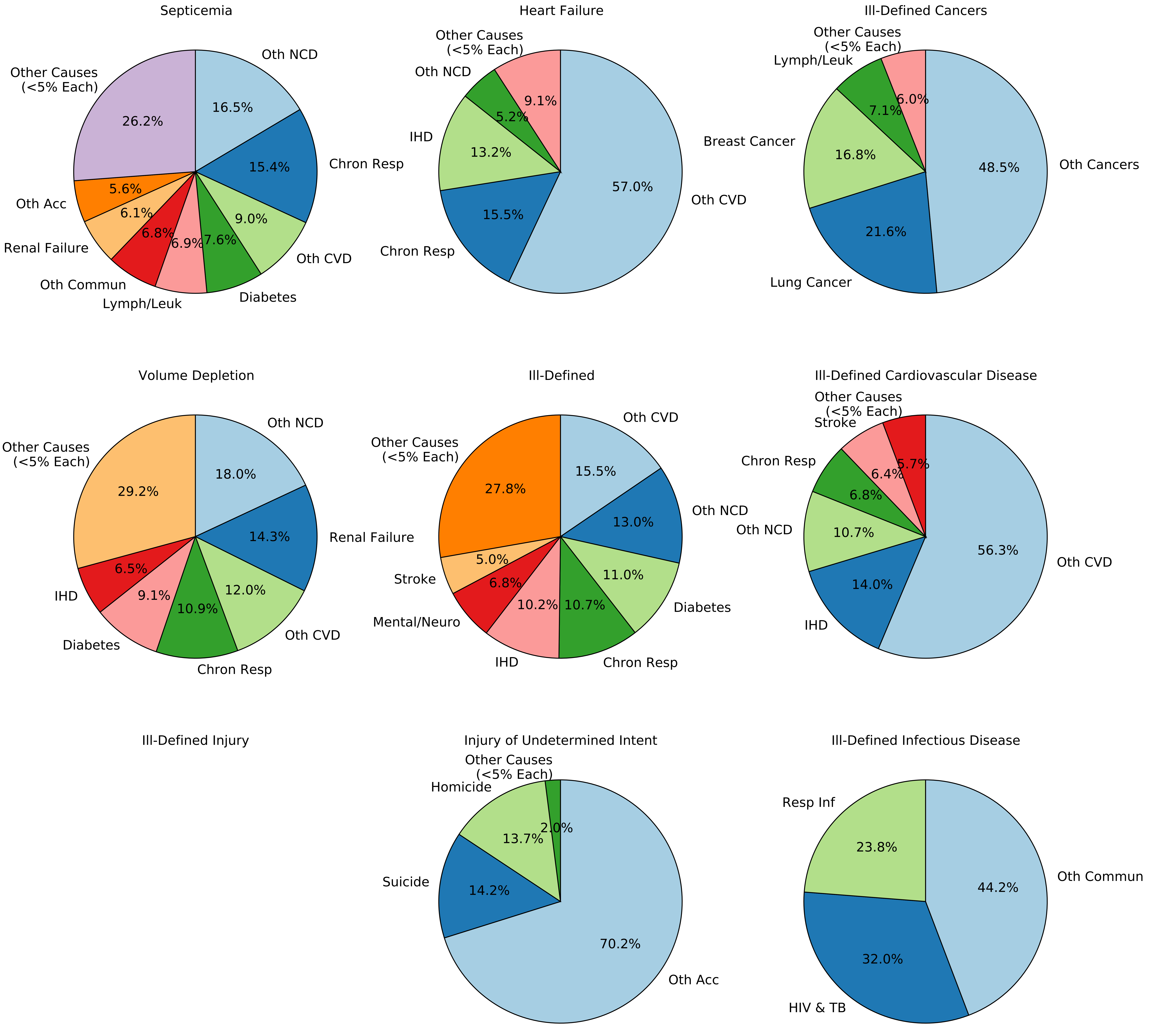

ICD 10
Female, Age 60

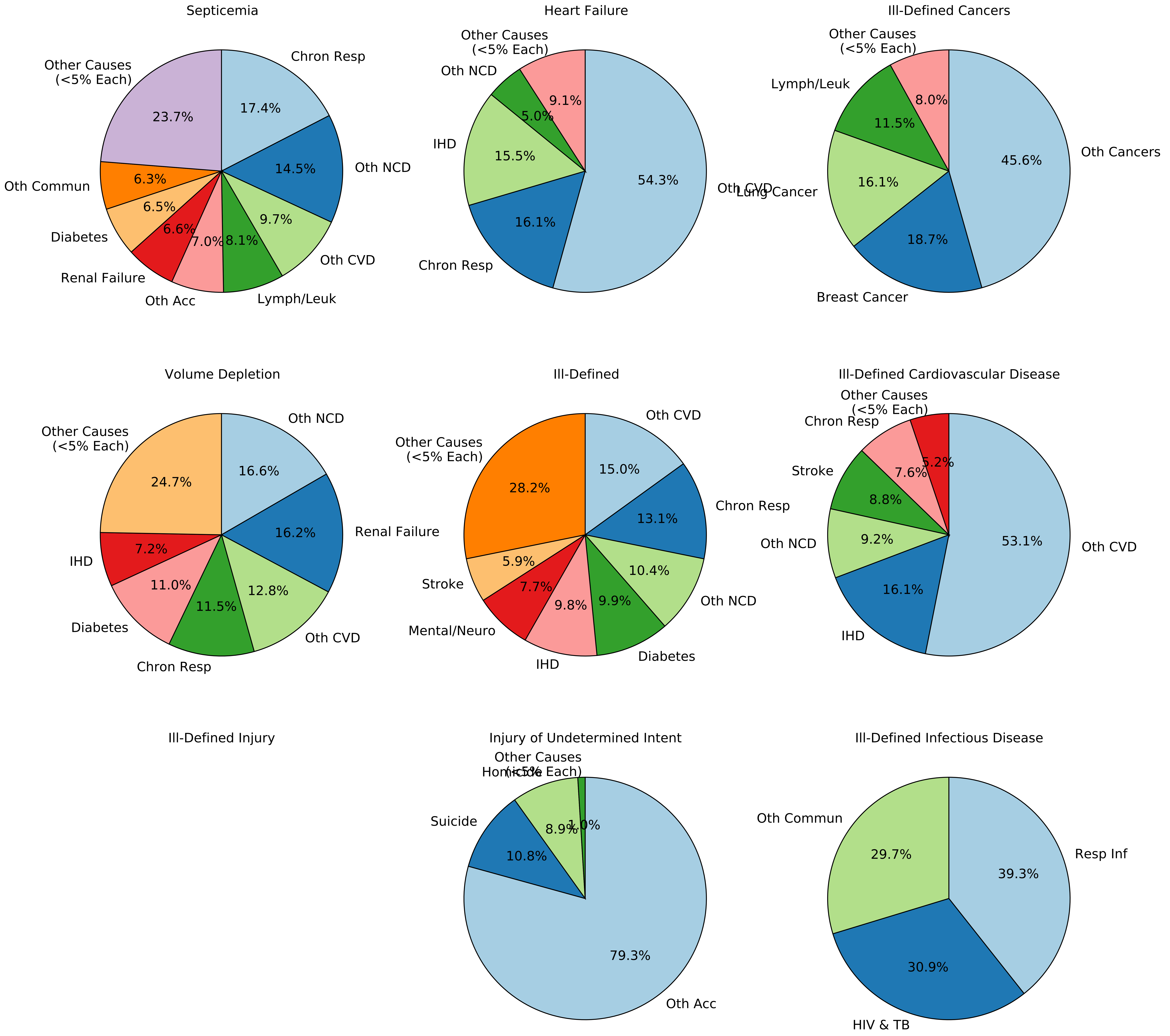

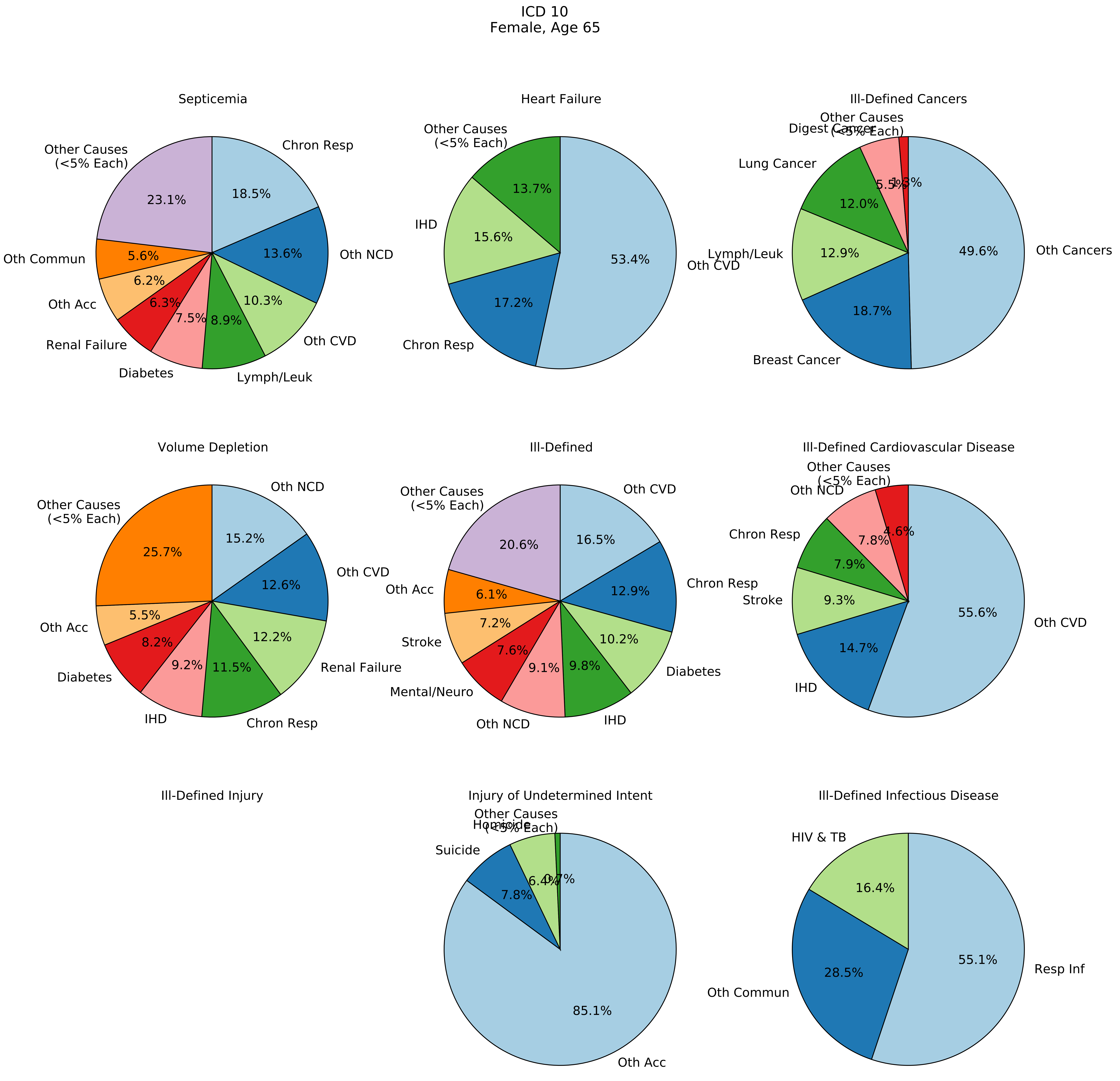

ICD 10
Female, Age 65
Septicemia
Chron Resp
18.5%
Oth NCD
13.6%
Oth CVD
10.3%
Lymph/Leuk
8.9%
Diabetes
7.5%
Renal Failure
6.3%
Oth Acc
6.2%
Oth Commun
5.6%
Other Causes (<5% Each)
23.1%
Heart Failure
Oth CVD
53.4%
Chron Resp
17.2%
IHD
15.6%
Other Causes (<5% Each)
13.7%
Ill-Defined Cancers
Oth Cancers
49.6%
Breast Cancer
18.7%
Lymph/Leuk
12.9%
Lung Cancer
12.0%
Digest Cancer
5.5%
Other Causes (<5% Each)
Volume Depletion
Oth NCD
15.2%
Oth CVD
12.6%
Renal Failure
12.2%
Chron Resp
11.5%
IHD
9.2%
Diabetes
8.2%
Oth Acc
5.5%
Other Causes (<5% Each)
25.7%
Ill-Defined
Oth CVD
16.5%
Chron Resp
12.9%
Diabetes
10.2%
IHD
9.8%
Oth NCD
9.1%
Mental/Neuro
7.6%
Stroke
7.2%
Oth Acc
6.1%
Other Causes (<5% Each)
20.6%
Ill-Defined Cardiovascular Disease
Oth CVD
55.6%
IHD
14.7%
Stroke
9.3%
Chron Resp
7.9%
Oth NCD
7.8%
Other Causes (<5% Each)
4.6%
Ill-Defined Injury
Injury of Undetermined Intent
Oth Acc
85.1%
Suicide
7.8%
Homicide
6.4%
Other Causes (<5% Each)
0.7%
Ill-Defined Infectious Disease
Resp Inf
55.1%
Oth Commun
28.5%
HIV & TB
16.4%

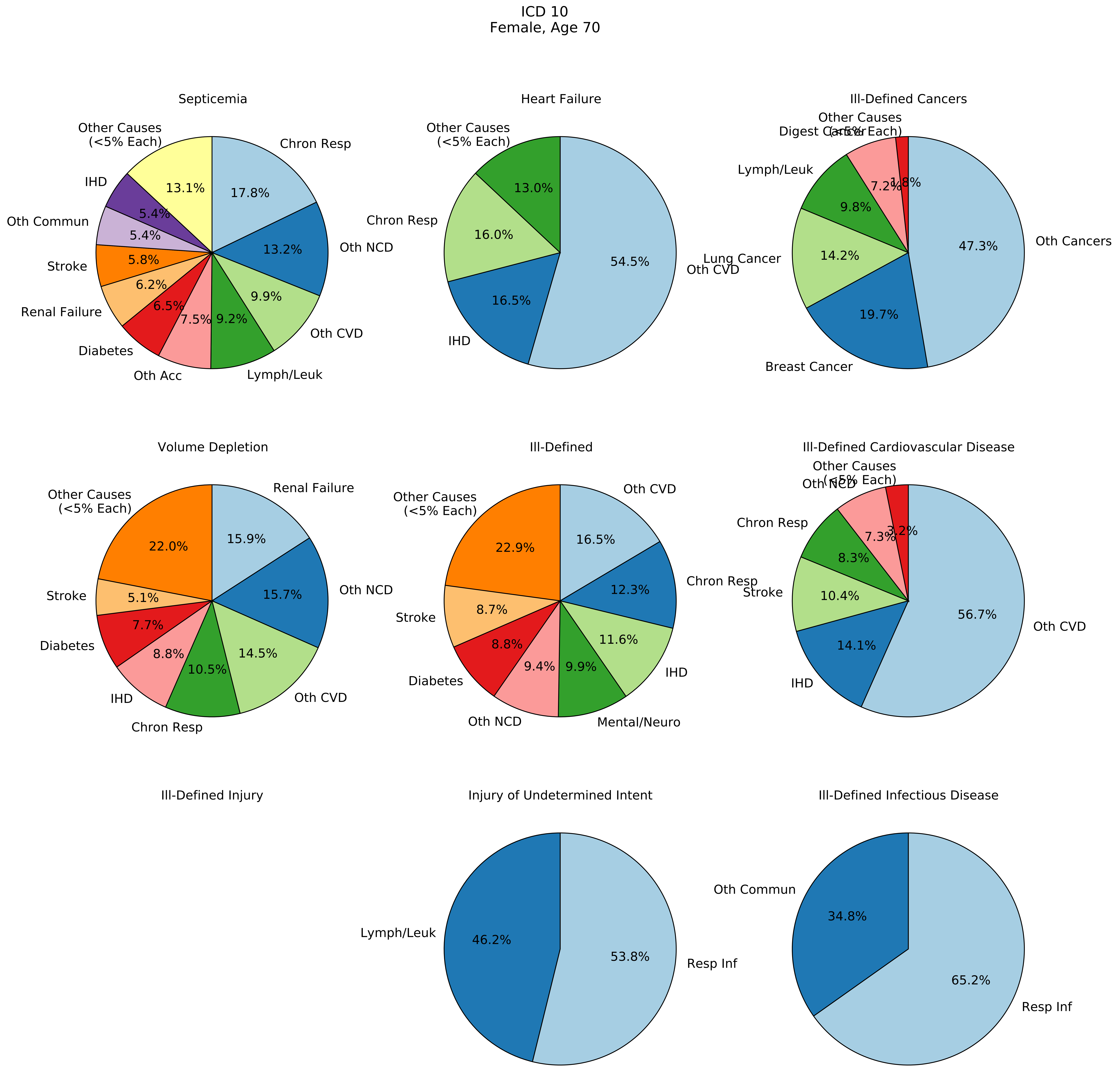
ICD 10
Female, Age 70
Septicemia
Other Causes
(<5% Each)
Chron Resp
IHD
Oth Commun
Stroke
Renal Failure
Diabetes
Oth Acc
Lymph/Leuk
Oth CVD
Oth NCD
13.1%
17.8%
5.4%
5.4%
5.8%
6.2%
6.5%
7.5%
9.2%
9.9%
13.2%
Heart Failure
Other Causes
(<5% Each)
Chron Resp
IHD
Oth CVD
13.0%
16.0%
16.5%
54.5%
Ill-Defined Cancers
Other Causes
(<5% Each)
Lymph/Leuk
Lung Cancer
Breast Cancer
Oth Cancers
7.2%
9.8%
14.2%
19.7%
47.3%
Volume Depletion
Other Causes
(<5% Each)
Renal Failure
Stroke
Diabetes
IHD
Chron Resp
Oth CVD
Oth NCD
22.0%
15.9%
5.1%
7.7%
8.8%
10.5%
14.5%
15.7%
Ill-Defined
Other Causes
(<5% Each)
Oth CVD
Chron Resp
Stroke
Diabetes
Oth NCD
Mental/Neuro
IHD
22.9%
16.5%
12.3%
8.7%
8.8%
9.4%
9.9%
11.6%
Ill-Defined Cardiovascular Disease
Other Causes
Chron Resp
Stroke
IHD
Oth CVD
7.3%
8.3%
10.4%
14.1%
56.7%
Ill-Defined Injury
Injury of Undetermined Intent
Lymph/Leuk
Resp Inf
46.2%
53.8%
Ill-Defined Infectious Disease
Oth Commun
Resp Inf
34.8%
65.2%

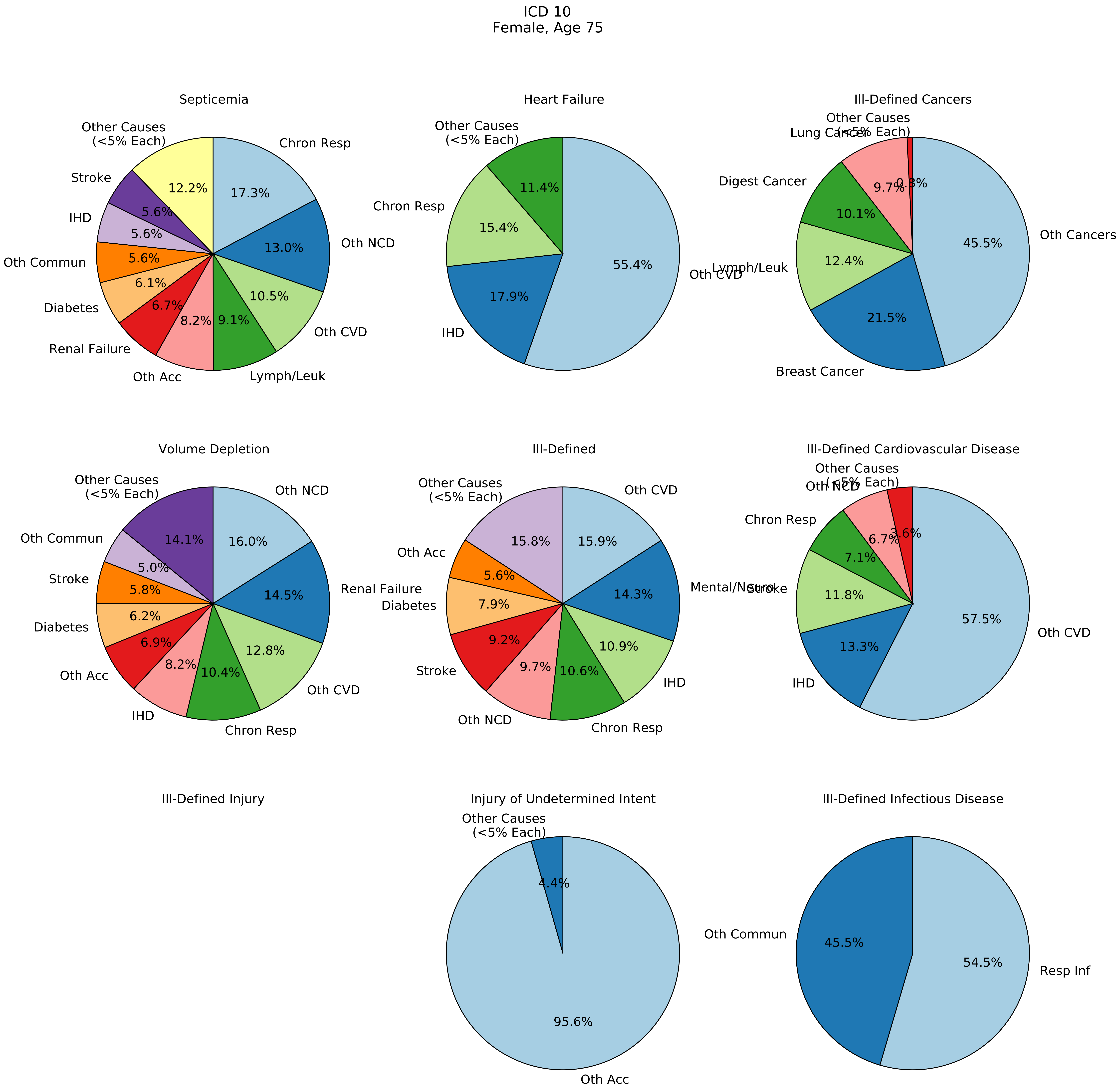
ICD 10
Female, Age 75
Septicemia
Chron Resp 17.3%
Oth NCD 13.0%
Oth CVD 10.5%
Lymph/Leuk 9.1%
Oth Acc 8.2%
Renal Failure 6.7%
Diabetes 6.1%
Oth Commun 5.6%
IHD 5.6%
Stroke 5.6%
Other Causes (<5% Each) 12.2%
Heart Failure
Oth CVD 55.4%
IHD 17.9%
Chron Resp 15.4%
Other Causes (<5% Each) 11.4%
Ill-Defined Cancers
Oth Cancers 45.5%
Breast Cancer 21.5%
Lymph/Leuk 12.4%
Digest Cancer 10.1%
Lung Cancer 9.7%
Other Causes (<5% Each) 0.8%
Volume Depletion
Oth NCD 16.0%
Renal Failure 14.5%
Oth CVD 12.8%
Chron Resp 10.4%
IHD 8.2%
Oth Acc 6.9%
Diabetes 6.2%
Stroke 5.8%
Oth Commun 5.0%
Other Causes (<5% Each) 14.1%
Ill-Defined
Oth CVD 15.9%
Mental/Neuro 14.3%
IHD 10.9%
Chron Resp 10.6%
Oth NCD 9.7%
Stroke 9.2%
Diabetes 7.9%
Oth Acc 5.6%
Other Causes (<5% Each) 15.8%
Ill-Defined Cardiovascular Disease
Oth CVD 57.5%
IHD 13.3%
Stroke 11.8%
Chron Resp 7.1%
Oth NCD 6.7%
Other Causes (<5% Each) 3.6%
Ill-Defined Injury
Injury of Undetermined Intent
Oth Acc 95.6%
Other Causes (<5% Each) 4.4%
Ill-Defined Infectious Disease
Resp Inf 54.5%
Oth Commun 45.5%

ICD 10
Female, Age 80

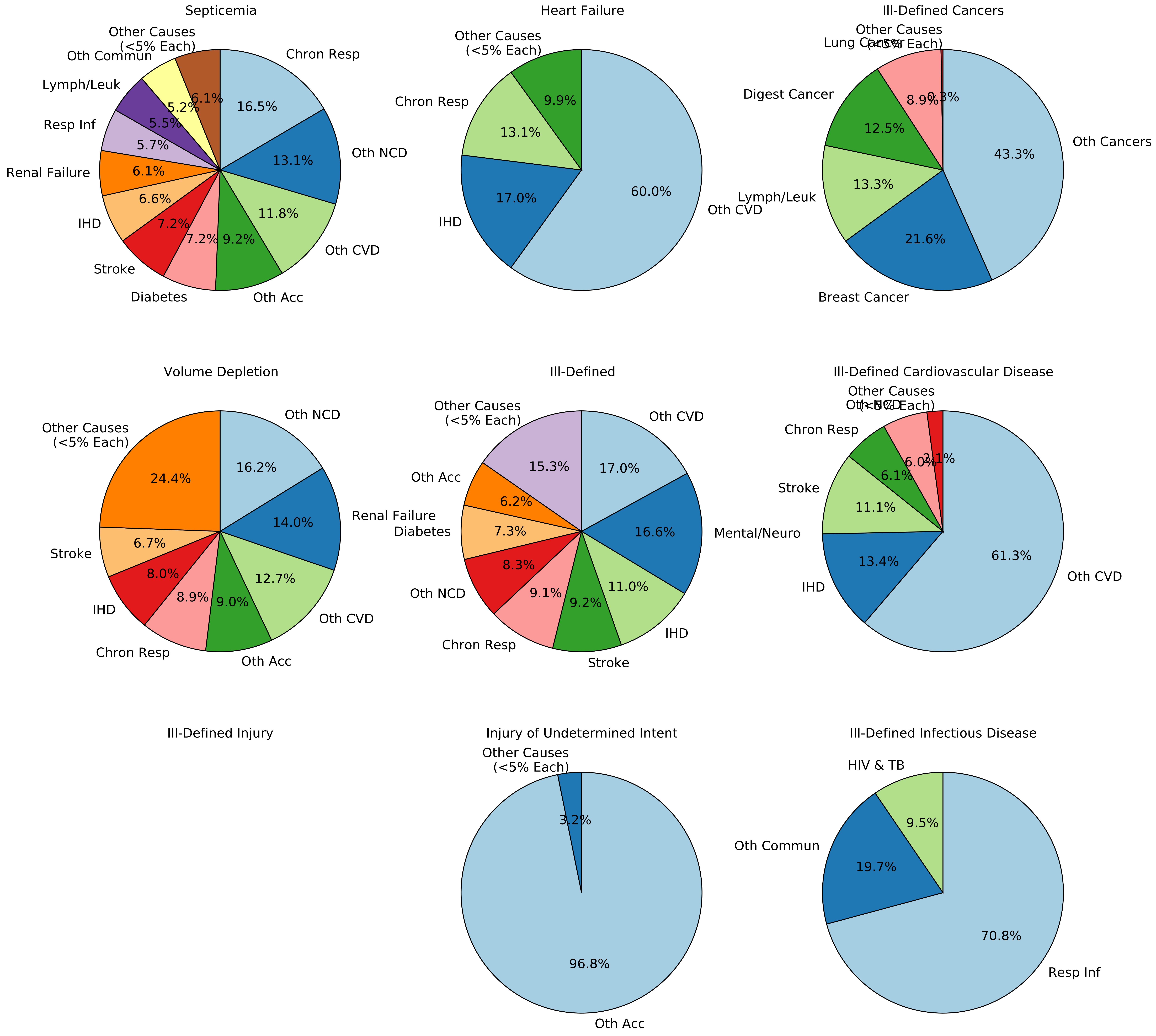

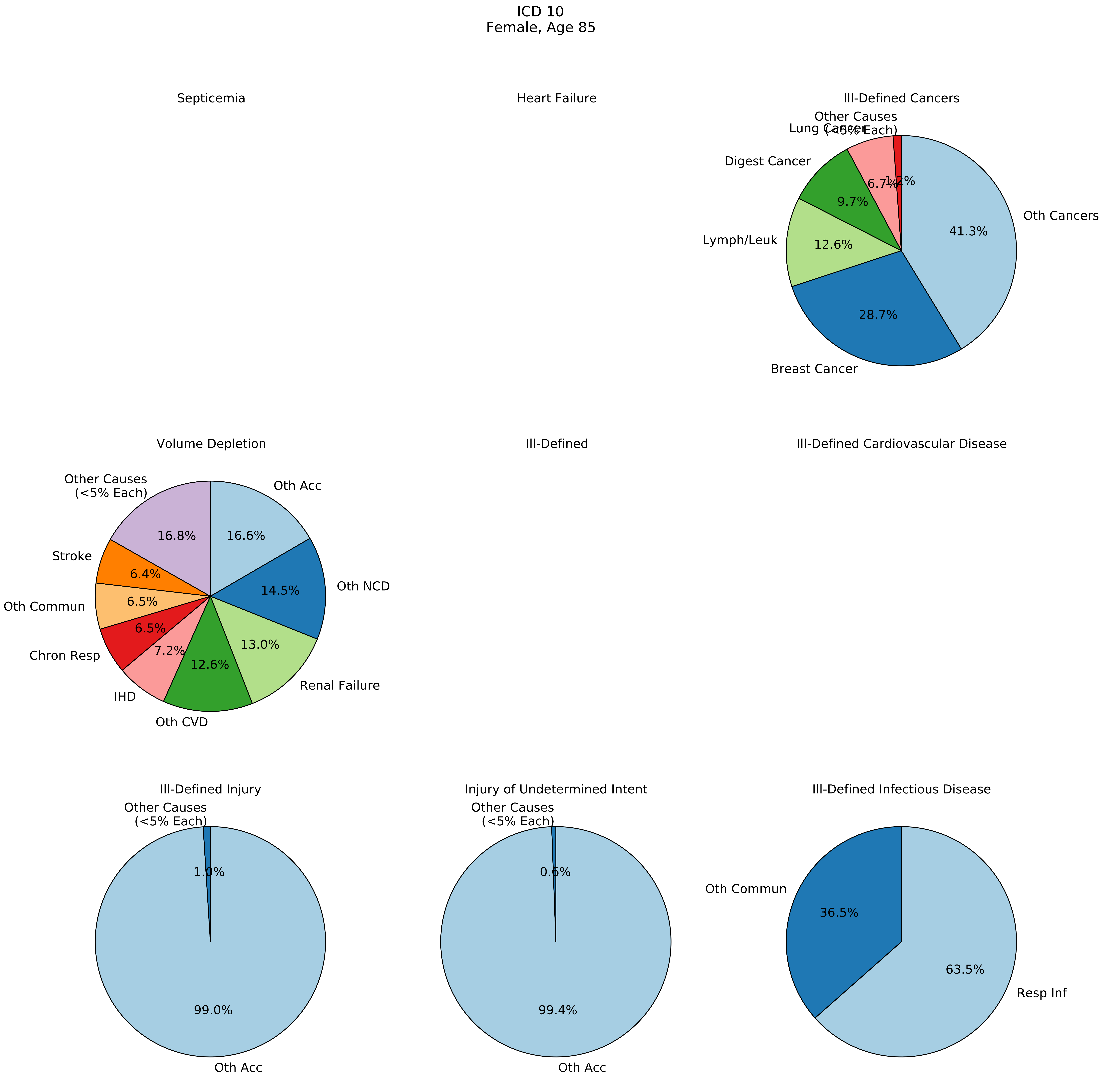

ICD 10
Female, Age 85
Septicemia
Heart Failure
Ill-Defined Cancers
Other Causes
(<5% Each)
Lung Cancer
Digest Cancer
Lymph/Leuk
Oth Cancers
Breast Cancer
6.7%
1.2%
9.7%
12.6%
41.3%
28.7%
Volume Depletion
Other Causes
(<5% Each)
Oth Acc
Stroke
Oth Commun
Chron Resp
IHD
Oth CVD
Renal Failure
Oth NCD
16.8%
16.6%
14.5%
6.4%
6.5%
6.5%
7.2%
12.6%
13.0%
Ill-Defined
Ill-Defined Cardiovascular Disease
Ill-Defined Injury
Other Causes
(<5% Each)
1.0%
99.0%
Oth Acc
Injury of Undetermined Intent
Other Causes
(<5% Each)
0.6%
99.4%
Oth Acc
Ill-Defined Infectious Disease
Oth Commun
36.5%
63.5%
Resp Inf

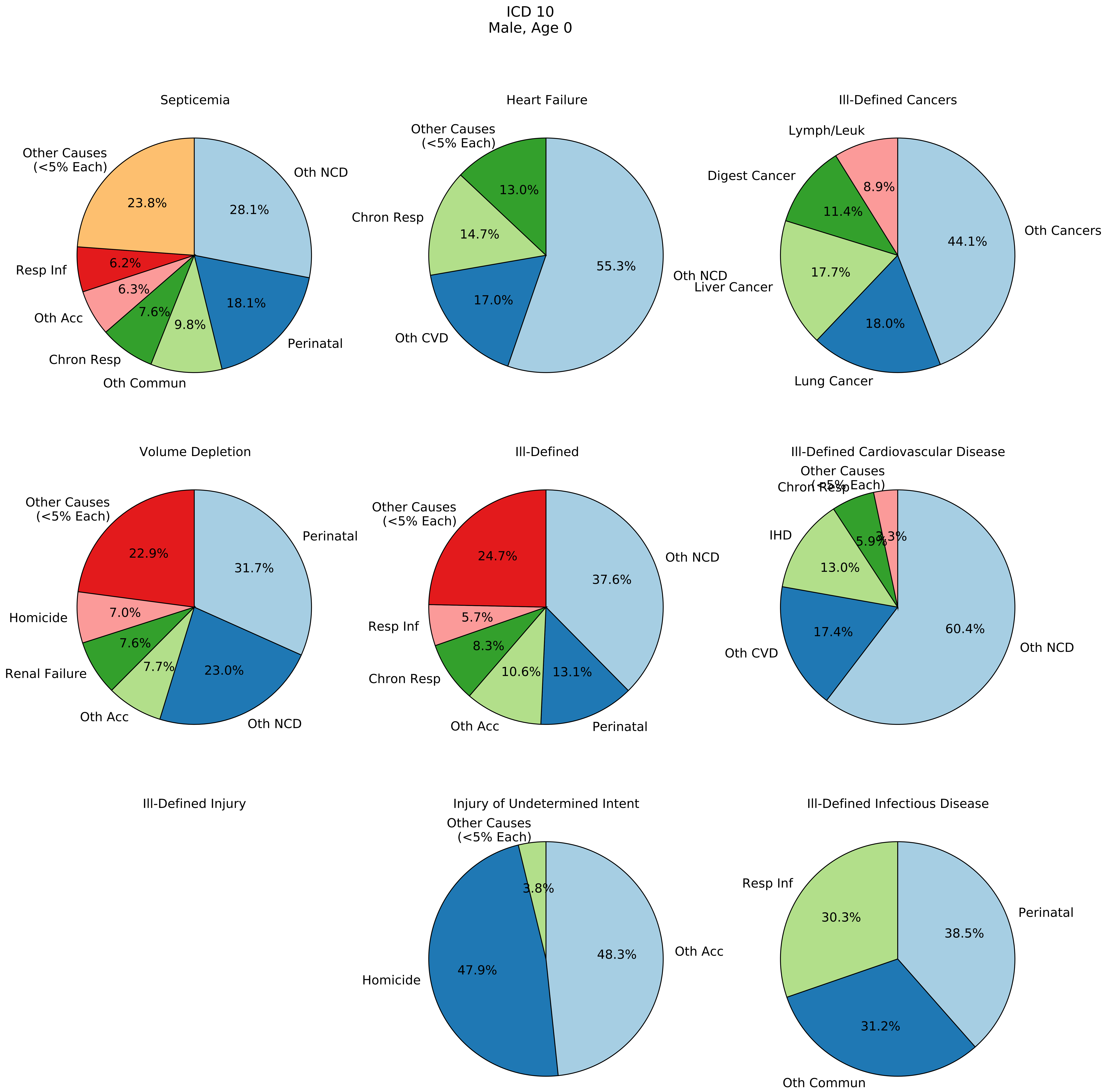
ICD 10
Male, Age 0
Septicemia
Other Causes
(<5% Each)
23.8%
Oth NCD
28.1%
Resp Inf
6.2%
Oth Acc
6.3%
Chron Resp
7.6%
Oth Commun
9.8%
18.1%
Perinatal
Heart Failure
Other Causes
(<5% Each)
13.0%
Chron Resp
14.7%
55.3%
Oth NCD
17.0%
Oth CVD
Ill-Defined Cancers
Lymph/Leuk
Digest Cancer
8.9%
11.4%
44.1%
Oth Cancers
17.7%
Liver Cancer
18.0%
Lung Cancer
Volume Depletion
Other Causes
(<5% Each)
22.9%
Perinatal
31.7%
Homicide
7.0%
Renal Failure
7.6%
7.7%
Oth Acc
23.0%
Oth NCD
Ill-Defined
Other Causes
(<5% Each)
24.7%
Oth NCD
37.6%
Resp Inf
5.7%
Chron Resp
8.3%
10.6%
13.1%
Oth Acc
Perinatal
Ill-Defined Cardiovascular Disease
Other Causes
Chron Resp
IHD
5.9%
13.0%
17.4%
Oth CVD
60.4%
Oth NCD
Ill-Defined Injury
Injury of Undetermined Intent
Other Causes
(<5% Each)
3.8%
48.3%
Oth Acc
Homicide
47.9%
Ill-Defined Infectious Disease
Resp Inf
30.3%
38.5%
Perinatal
31.2%
Oth Commun

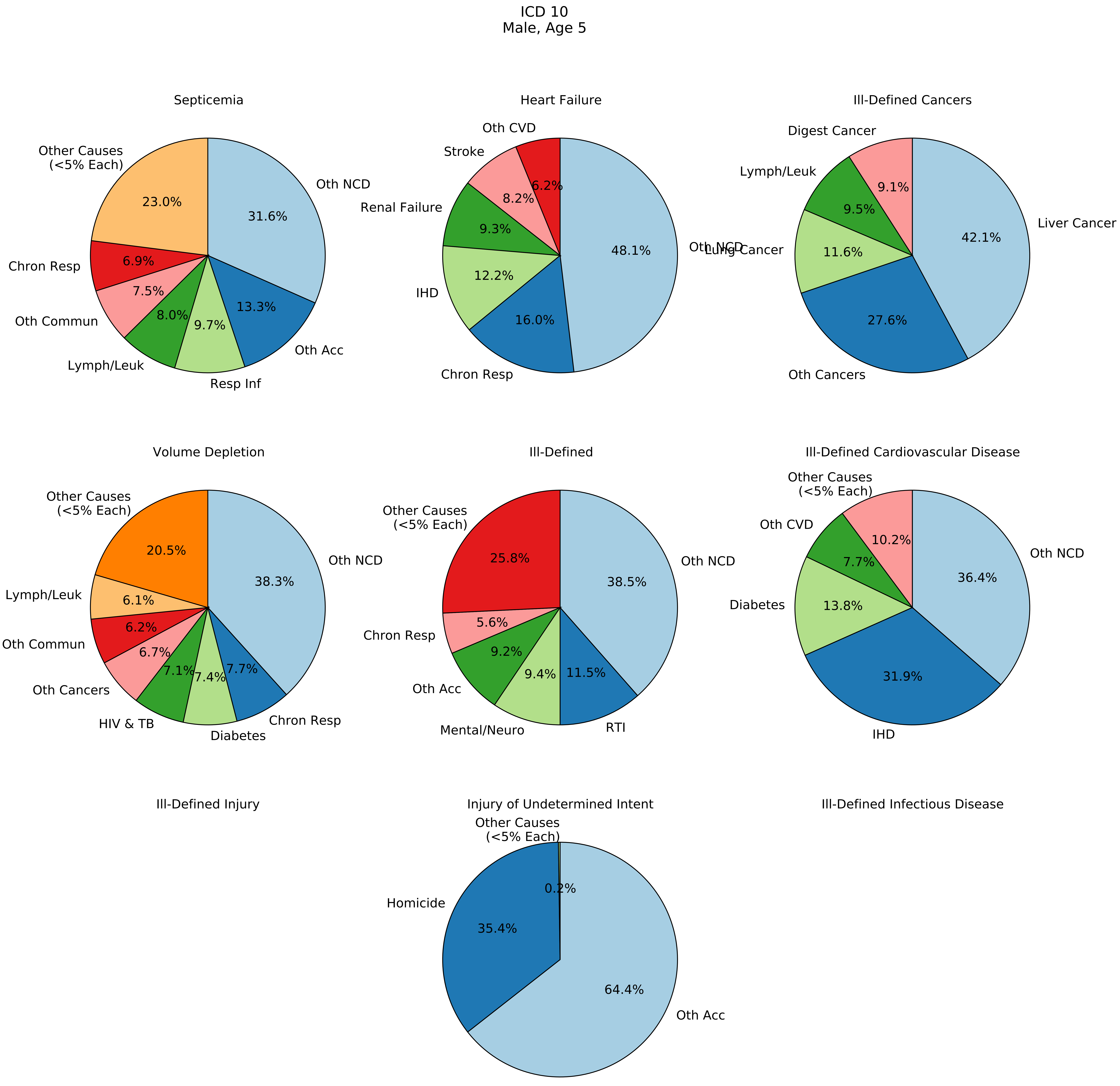
ICD 10
Male, Age 5
Septicemia
Other Causes (<5% Each)
23.0%
Oth NCD
31.6%
Chron Resp
6.9%
Oth Commun
7.5%
Lymph/Leuk
8.0%
Resp Inf
9.7%
Oth Acc
13.3%
Heart Failure
Oth CVD
6.2%
Stroke
8.2%
Renal Failure
9.3%
IHD
12.2%
Chron Resp
16.0%
Oth NCD
48.1%
Ill-Defined Cancers
Digest Cancer
9.1%
Lymph/Leuk
9.5%
Lung Cancer
11.6%
Oth Cancers
27.6%
Liver Cancer
42.1%
Volume Depletion
Other Causes (<5% Each)
20.5%
Lymph/Leuk
6.1%
Oth Commun
6.2%
Oth Cancers
6.7%
HIV & TB
7.1%
Diabetes
7.4%
Chron Resp
7.7%
Oth NCD
38.3%
Ill-Defined
Other Causes (<5% Each)
25.8%
Chron Resp
5.6%
Oth Acc
9.2%
Mental/Neuro
9.4%
RTI
11.5%
Oth NCD
38.5%
Ill-Defined Cardiovascular Disease
Other Causes (<5% Each)
10.2%
Oth CVD
7.7%
Diabetes
13.8%
IHD
31.9%
Oth NCD
36.4%
Ill-Defined Injury
Injury of Undetermined Intent
Other Causes (<5% Each)
0.2%
Homicide
35.4%
Oth Acc
64.4%
Ill-Defined Infectious Disease

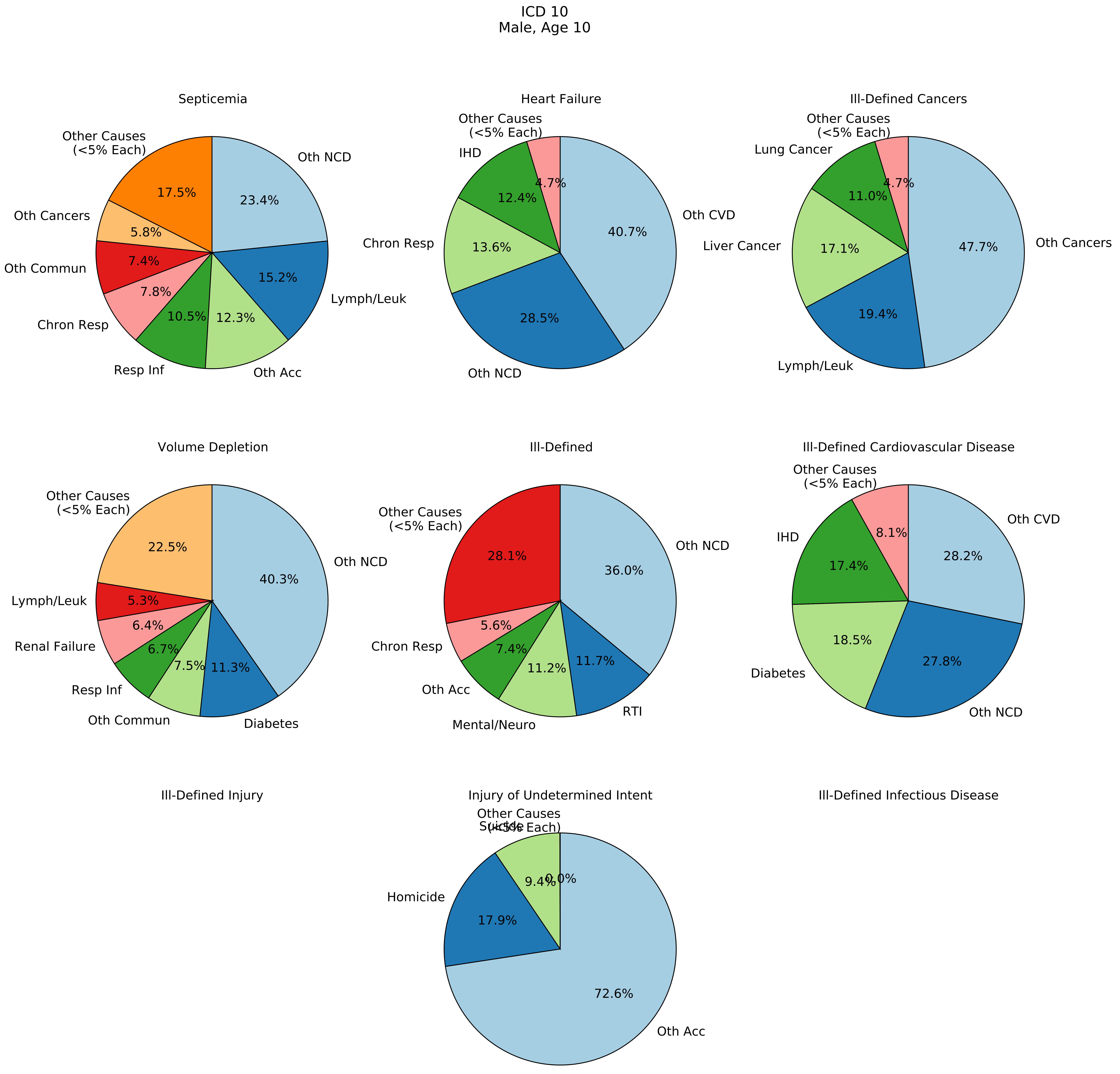
ICD 10
Male, Age 10
Septicemia
Other Causes
(<5% Each)
17.5%
Oth NCD
23.4%
Oth Cancers
5.8%
Oth Commun
7.4%
Chron Resp
7.8%
Resp Inf
10.5%
Oth Acc
12.3%
Lymph/Leuk
15.2%
Heart Failure
Other Causes
(<5% Each)
4.7%
IHD
12.4%
Chron Resp
13.6%
Oth CVD
40.7%
Oth NCD
28.5%
Ill-Defined Cancers
Other Causes
(<5% Each)
4.7%
Lung Cancer
11.0%
Liver Cancer
17.1%
Oth Cancers
47.7%
Lymph/Leuk
19.4%
Volume Depletion
Other Causes
(<5% Each)
22.5%
Oth NCD
40.3%
Lymph/Leuk
5.3%
Renal Failure
6.4%
Resp Inf
6.7%
Oth Commun
7.5%
Diabetes
11.3%
Ill-Defined
Other Causes
(<5% Each)
28.1%
Oth NCD
36.0%
Chron Resp
5.6%
Oth Acc
7.4%
Mental/Neuro
11.2%
RTI
11.7%
Ill-Defined Cardiovascular Disease
Other Causes
(<5% Each)
8.1%
Oth CVD
28.2%
IHD
17.4%
Diabetes
18.5%
Oth NCD
27.8%
Ill-Defined Injury
Injury of Undetermined Intent
Other Causes
(<5% Each)
Suicide
0.0%
9.4%
Homicide
17.9%
Oth Acc
72.6%
Ill-Defined Infectious Disease

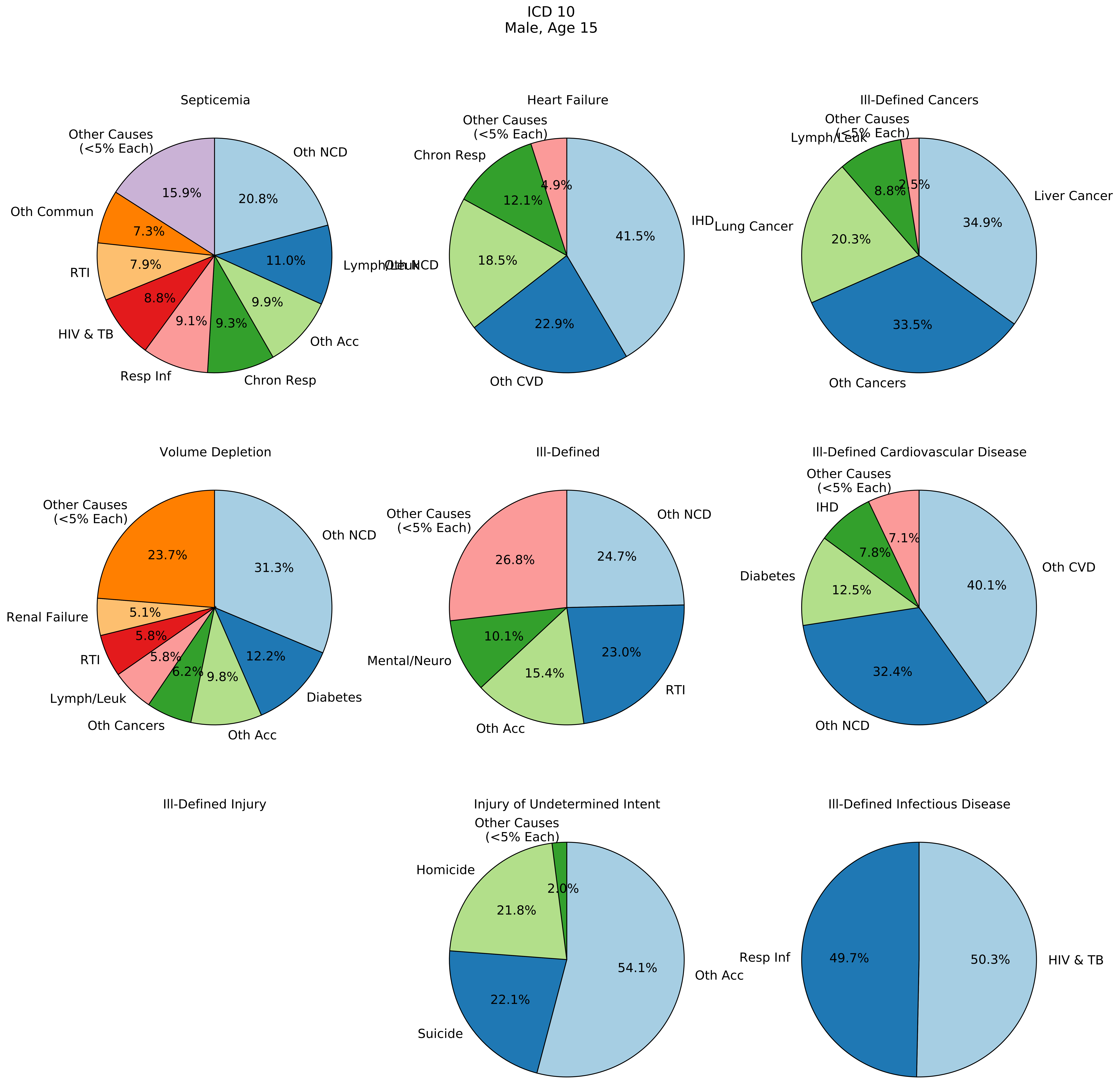
ICD 10
Male, Age 15
Septicemia
Other Causes (<5% Each)
15.9%
Oth NCD
20.8%
Lymph/Leuk
11.0%
Oth Acc
9.9%
Chron Resp
9.3%
Resp Inf
9.1%
HIV & TB
8.8%
RTI
7.9%
Oth Commun
7.3%
Heart Failure
Other Causes (<5% Each)
4.9%
IHD
41.5%
Oth CVD
22.9%
Oth NCD
18.5%
Chron Resp
12.1%
Ill-Defined Cancers
Other Causes (<5% Each)
2.5%
Liver Cancer
34.9%
Oth Cancers
33.5%
Lung Cancer
20.3%
Lymph/Leuk
8.8%
Volume Depletion
Other Causes (<5% Each)
23.7%
Oth NCD
31.3%
Diabetes
12.2%
Oth Acc
9.8%
Oth Cancers
6.2%
Lymph/Leuk
5.8%
RTI
5.8%
Renal Failure
5.1%
Ill-Defined
Other Causes (<5% Each)
26.8%
Oth NCD
24.7%
RTI
23.0%
Oth Acc
15.4%
Mental/Neuro
10.1%
Ill-Defined Cardiovascular Disease
Other Causes (<5% Each)
7.1%
Oth CVD
40.1%
Oth NCD
32.4%
Diabetes
12.5%
IHD
7.8%
Ill-Defined Injury
Injury of Undetermined Intent
Other Causes (<5% Each)
2.0%
Oth Acc
54.1%
Suicide
22.1%
Homicide
21.8%
Ill-Defined Infectious Disease
HIV & TB
50.3%
Resp Inf
49.7%

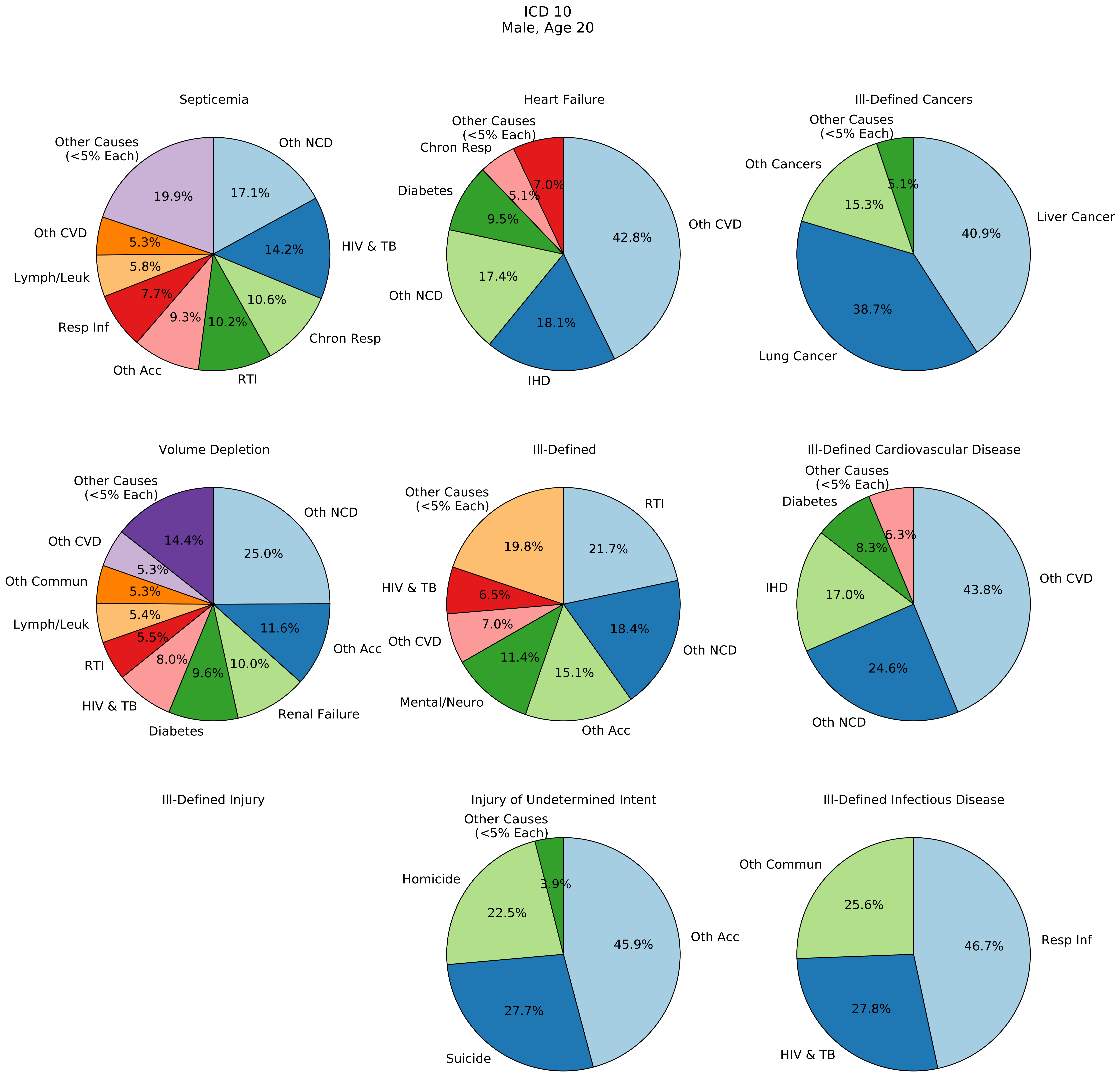
ICD 10
Male, Age 20
Septicemia
Oth NCD 17.1%
HIV & TB 14.2%
Chron Resp 10.6%
RTI 10.2%
Oth Acc 9.3%
Resp Inf 7.7%
Lymph/Leuk 5.8%
Oth CVD 5.3%
Other Causes (<5% Each) 19.9%
Heart Failure
Oth CVD 42.8%
IHD 18.1%
Oth NCD 17.4%
Diabetes 9.5%
Chron Resp 5.1%
Other Causes (<5% Each) 7.0%
Ill-Defined Cancers
Liver Cancer 40.9%
Lung Cancer 38.7%
Oth Cancers 15.3%
Other Causes (<5% Each) 5.1%
Volume Depletion
Oth NCD 25.0%
Oth Acc 11.6%
Renal Failure 10.0%
Diabetes 9.6%
HIV & TB 8.0%
RTI 5.5%
Lymph/Leuk 5.4%
Oth Commun 5.3%
Oth CVD 5.3%
Other Causes (<5% Each) 14.4%
Ill-Defined
RTI 21.7%
Oth NCD 18.4%
Oth Acc 15.1%
Mental/Neuro 11.4%
Oth CVD 7.0%
HIV & TB 6.5%
Other Causes (<5% Each) 19.8%
Ill-Defined Cardiovascular Disease
Oth CVD 43.8%
Oth NCD 24.6%
IHD 17.0%
Diabetes 8.3%
Other Causes (<5% Each) 6.3%
Ill-Defined Injury
Injury of Undetermined Intent
Oth Acc 45.9%
Suicide 27.7%
Homicide 22.5%
Other Causes (<5% Each) 3.9%
Ill-Defined Infectious Disease
Resp Inf 46.7%
HIV & TB 27.8%
Oth Commun 25.6%

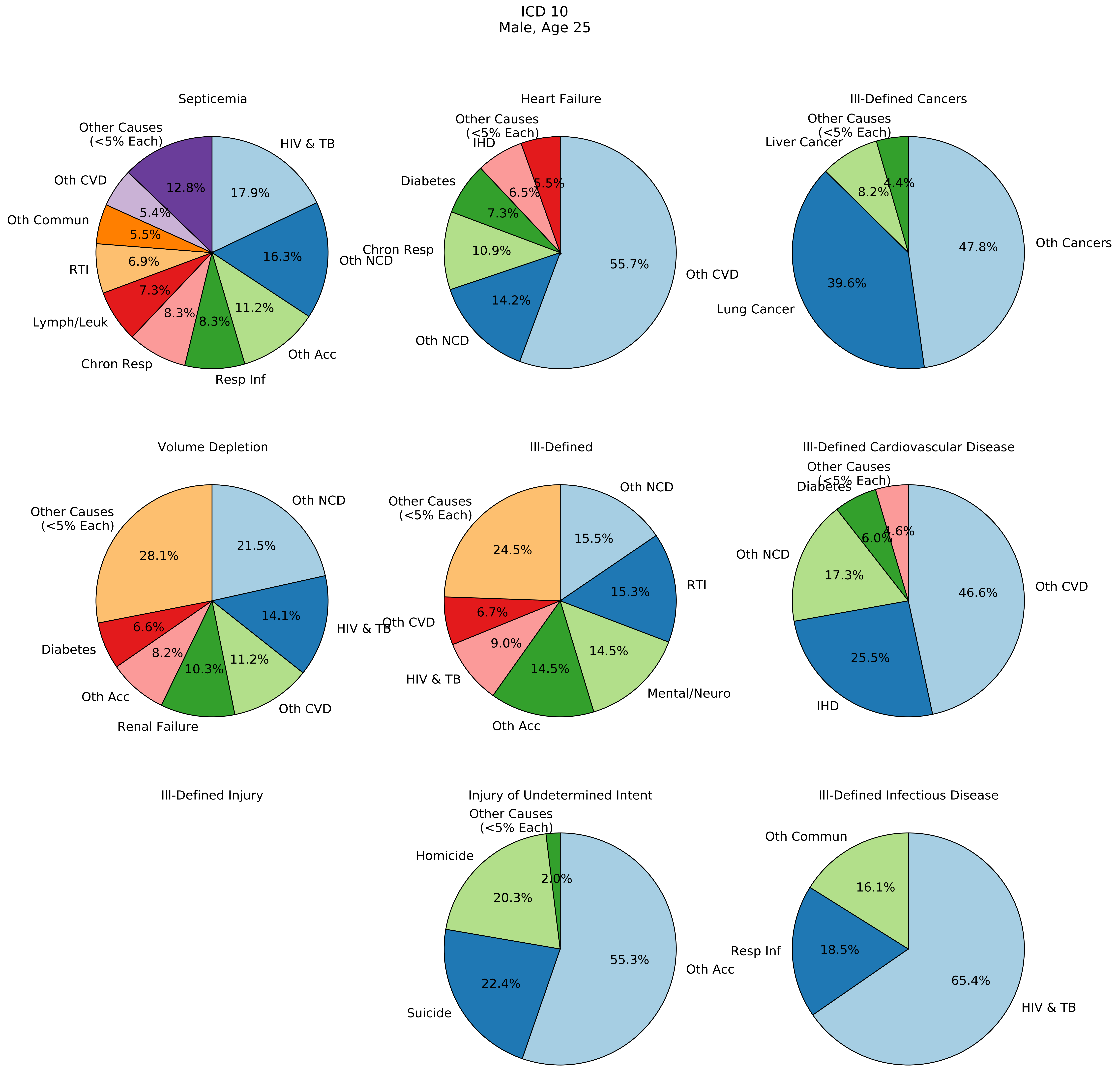
ICD 10
Male, Age 25
Septicemia
HIV & TB 17.9%
Oth NCD 16.3%
Oth Acc 11.2%
Resp Inf 8.3%
Chron Resp 8.3%
Lymph/Leuk 7.3%
RTI 6.9%
Oth Commun 5.5%
Oth CVD 5.4%
Other Causes (<5% Each) 12.8%
Heart Failure
Oth CVD 55.7%
Oth NCD 14.2%
Chron Resp 10.9%
Diabetes 7.3%
IHD 6.5%
Other Causes (<5% Each) 5.5%
Ill-Defined Cancers
Oth Cancers 47.8%
Lung Cancer 39.6%
Liver Cancer 8.2%
Other Causes (<5% Each) 4.4%
Volume Depletion
Oth NCD 21.5%
HIV & TB 14.1%
Oth CVD 11.2%
Renal Failure 10.3%
Oth Acc 8.2%
Diabetes 6.6%
Other Causes (<5% Each) 28.1%
Ill-Defined
Oth NCD 15.5%
RTI 15.3%
Mental/Neuro 14.5%
Oth Acc 14.5%
HIV & TB 9.0%
Oth CVD 6.7%
Other Causes (<5% Each) 24.5%
Ill-Defined Cardiovascular Disease
Oth CVD 46.6%
IHD 25.5%
Oth NCD 17.3%
Diabetes 6.0%
Other Causes (<5% Each) 4.6%
Ill-Defined Injury
Injury of Undetermined Intent
Oth Acc 55.3%
Suicide 22.4%
Homicide 20.3%
Other Causes (<5% Each) 2.0%
Ill-Defined Infectious Disease
HIV & TB 65.4%
Resp Inf 18.5%
Oth Commun 16.1%

ICD 10
Male, Age 30

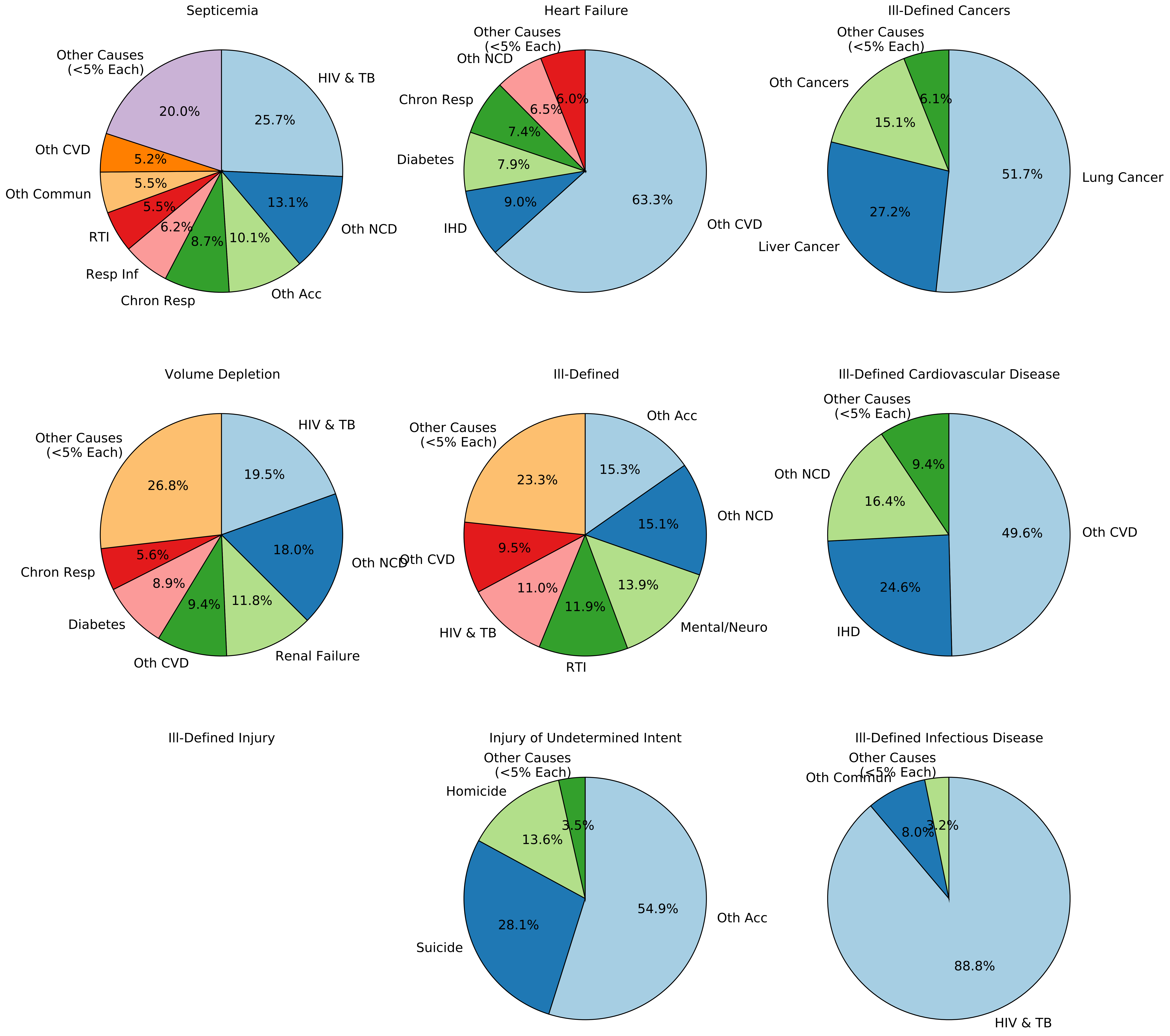

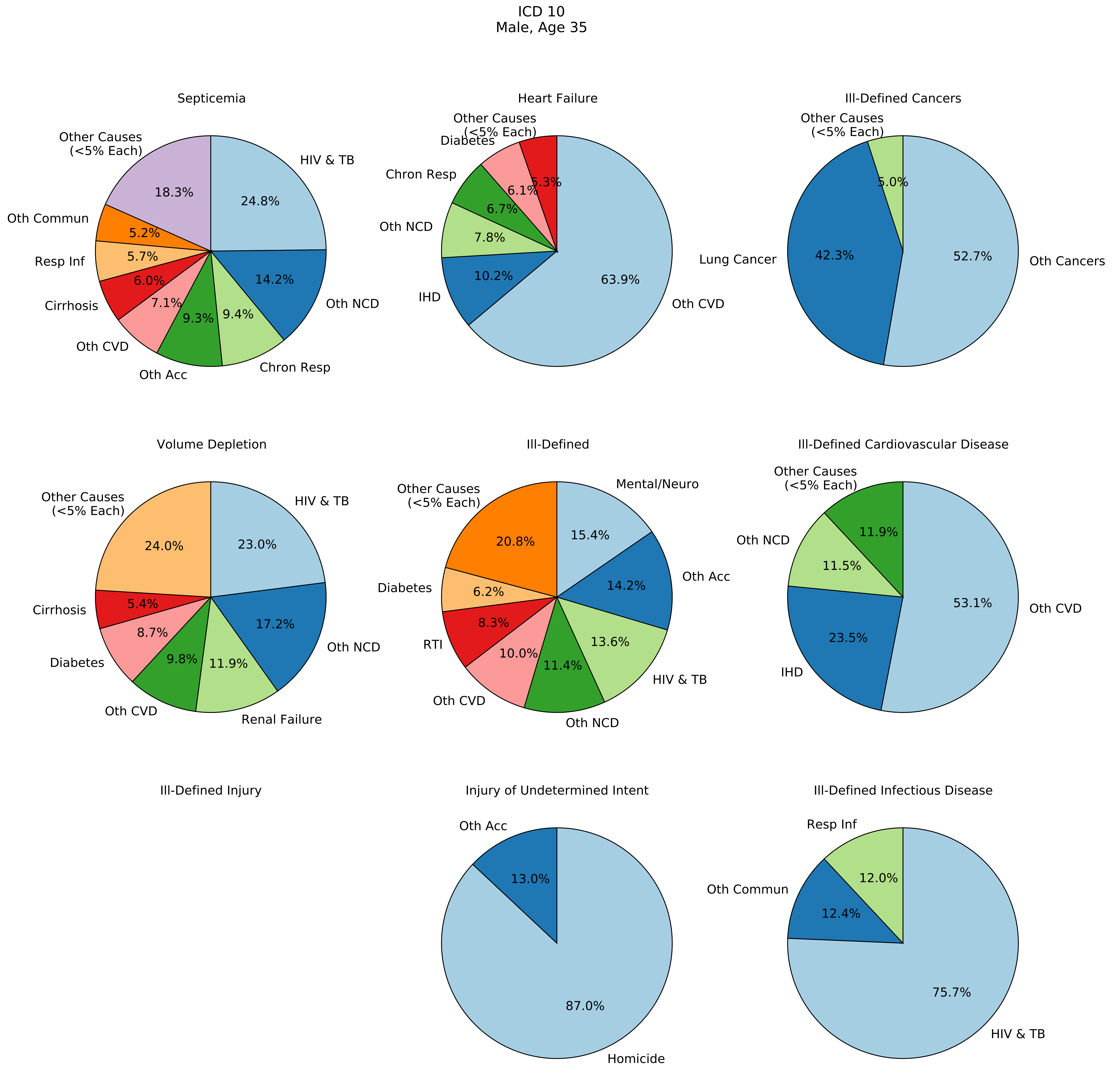
ICD 10
Male, Age 35
Septicemia
Other Causes (<5% Each)
18.3%
HIV & TB
24.8%
Oth Commun
5.2%
Resp Inf
5.7%
Cirrhosis
6.0%
Oth CVD
7.1%
Oth Acc
9.3%
Chron Resp
9.4%
Oth NCD
14.2%
Heart Failure
Other Causes (<5% Each)
5.3%
Diabetes
6.1%
Chron Resp
6.7%
Oth NCD
7.8%
IHD
10.2%
Oth CVD
63.9%
Ill-Defined Cancers
Other Causes (<5% Each)
5.0%
Lung Cancer
42.3%
Oth Cancers
52.7%
Volume Depletion
Other Causes (<5% Each)
24.0%
HIV & TB
23.0%
Cirrhosis
5.4%
Diabetes
8.7%
Oth CVD
9.8%
Renal Failure
11.9%
Oth NCD
17.2%
Ill-Defined
Other Causes (<5% Each)
20.8%
Mental/Neuro
15.4%
Oth Acc
14.2%
HIV & TB
13.6%
Oth NCD
11.4%
Oth CVD
10.0%
RTI
8.3%
Diabetes
6.2%
Ill-Defined Cardiovascular Disease
Other Causes (<5% Each)
11.9%
Oth NCD
11.5%
IHD
23.5%
Oth CVD
53.1%
Ill-Defined Injury
Injury of Undetermined Intent
Oth Acc
13.0%
Homicide
87.0%
Ill-Defined Infectious Disease
Resp Inf
12.0%
Oth Commun
12.4%
HIV & TB
75.7%

ICD 10
Male, Age 40

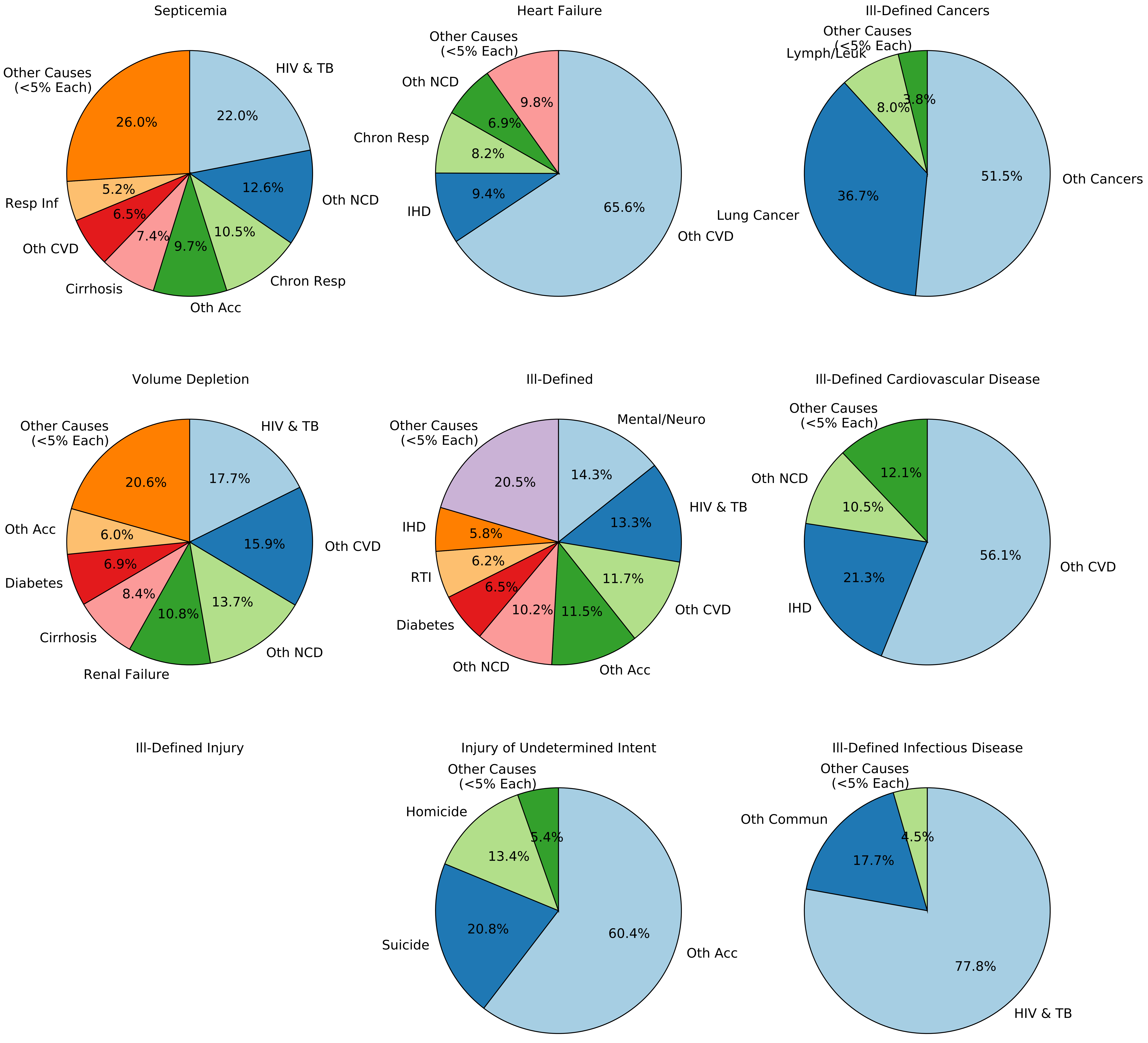

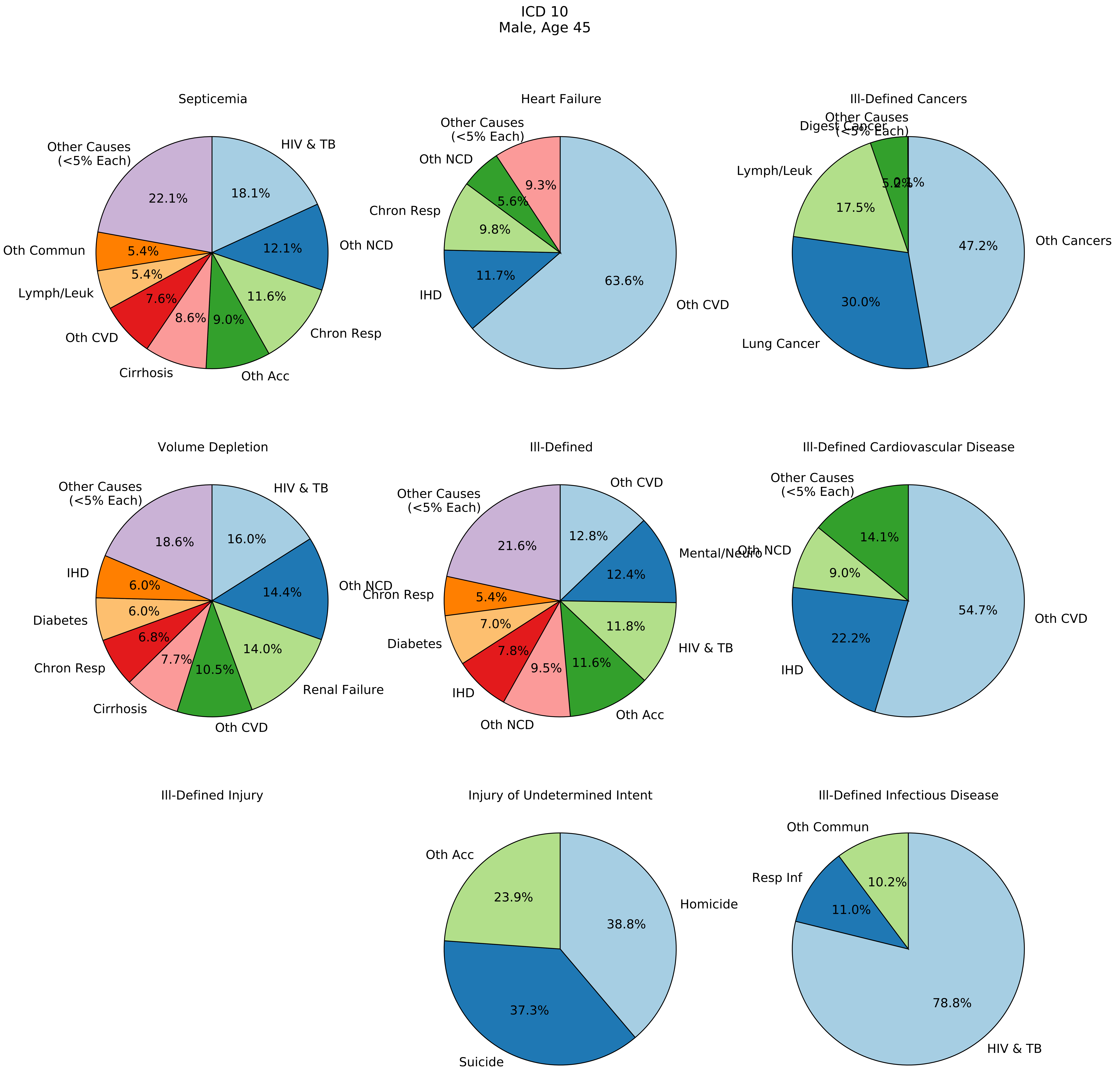

ICD 10
Male, Age 45
Septicemia
HIV & TB
18.1%
Oth NCD
12.1%
Chron Resp
11.6%
Oth Acc
9.0%
Cirrhosis
8.6%
Oth CVD
7.6%
Lymph/Leuk
5.4%
Oth Commun
5.4%
Other Causes
(<5% Each)
22.1%
Heart Failure
Oth CVD
63.6%
IHD
11.7%
Chron Resp
9.8%
Oth NCD
5.6%
Other Causes
(<5% Each)
9.3%
Ill-Defined Cancers
Oth Cancers
47.2%
Lung Cancer
30.0%
Lymph/Leuk
17.5%
Digest Cancer
Other Causes
(<5% Each)
Volume Depletion
HIV & TB
16.0%
Oth NCD
14.4%
Renal Failure
14.0%
Oth CVD
10.5%
Cirrhosis
7.7%
Chron Resp
6.8%
Diabetes
6.0%
IHD
6.0%
Other Causes
(<5% Each)
18.6%
Ill-Defined
Oth CVD
12.8%
Mental/Neuro
12.4%
HIV & TB
11.8%
Oth Acc
11.6%
Oth NCD
9.5%
IHD
7.8%
Diabetes
7.0%
Chron Resp
5.4%
Other Causes
(<5% Each)
21.6%
Ill-Defined Cardiovascular Disease
Oth CVD
54.7%
IHD
22.2%
Oth NCD
9.0%
Other Causes
(<5% Each)
14.1%
Ill-Defined Injury
Injury of Undetermined Intent
Homicide
38.8%
Suicide
37.3%
Oth Acc
23.9%
Ill-Defined Infectious Disease
HIV & TB
78.8%
Resp Inf
11.0%
Oth Commun
10.2%

ICD 10
Male, Age 50

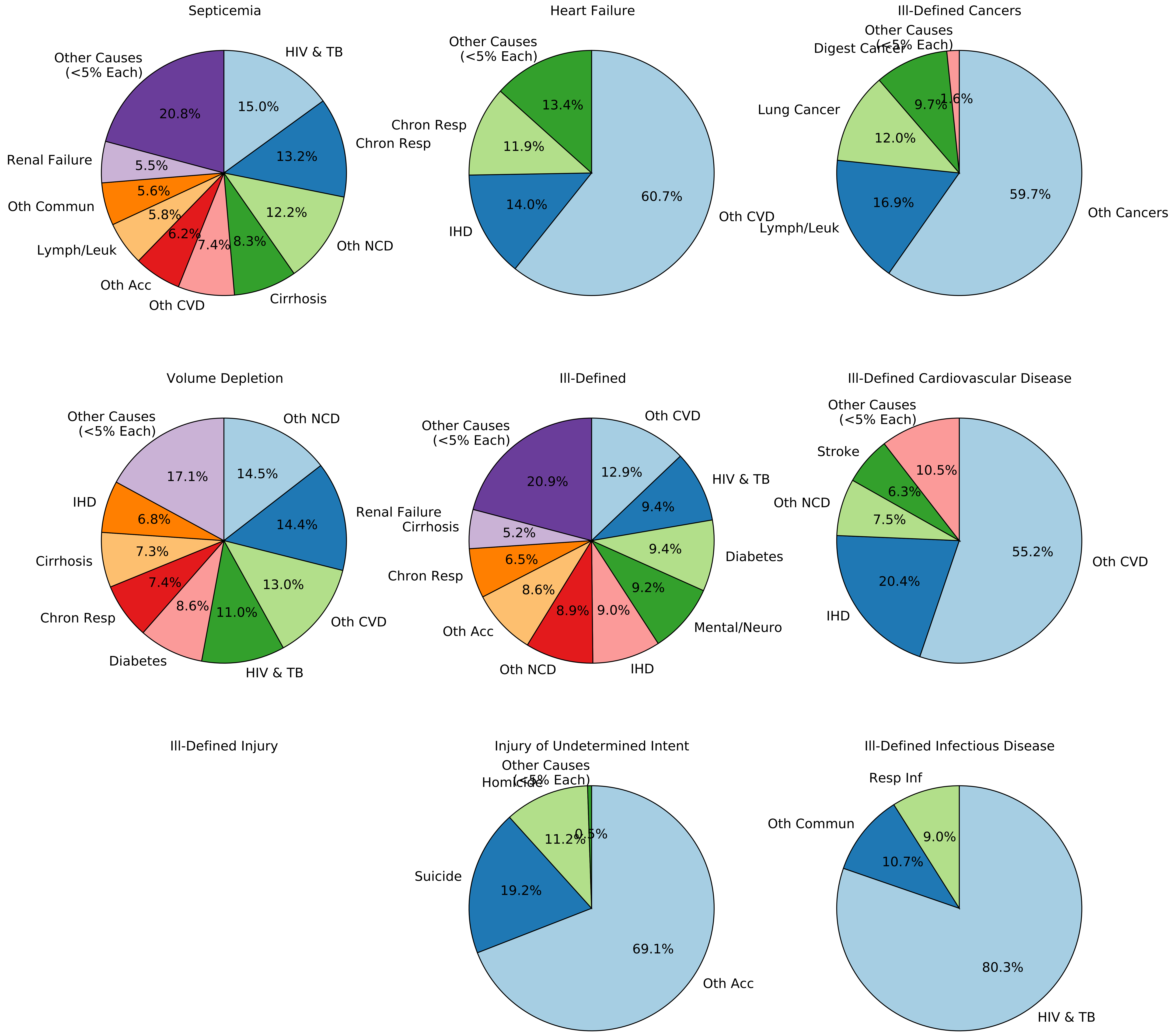

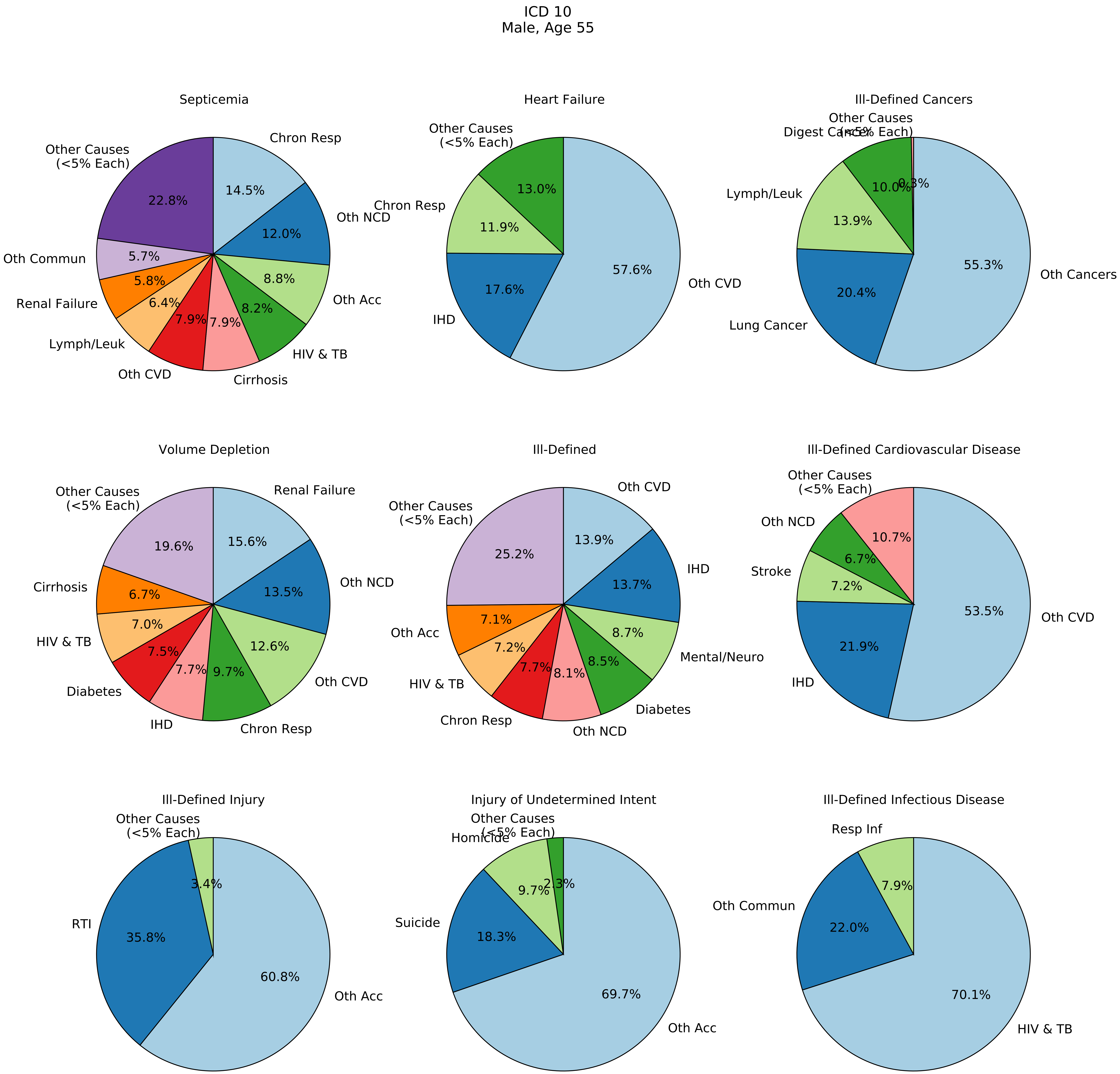

ICD 10
Male, Age 55
Septicemia
Chron Resp
14.5%
Oth NCD
12.0%
Oth Acc
8.8%
HIV & TB
8.2%
Cirrhosis
7.9%
Oth CVD
7.9%
Lymph/Leuk
6.4%
Renal Failure
5.8%
Oth Commun
5.7%
Other Causes (<5% Each)
22.8%
Heart Failure
Oth CVD
57.6%
IHD
17.6%
Chron Resp
11.9%
Other Causes (<5% Each)
13.0%
Ill-Defined Cancers
Oth Cancers
55.3%
Lung Cancer
20.4%
Lymph/Leuk
13.9%
Digest Cancer
10.0%
Other Causes (<5% Each)
Volume Depletion
Renal Failure
15.6%
Oth NCD
13.5%
Oth CVD
12.6%
Chron Resp
9.7%
IHD
7.7%
Diabetes
7.5%
HIV & TB
7.0%
Cirrhosis
6.7%
Other Causes (<5% Each)
19.6%
Ill-Defined
Oth CVD
13.9%
IHD
13.7%
Mental/Neuro
8.7%
Diabetes
8.5%
Oth NCD
8.1%
Chron Resp
7.7%
HIV & TB
7.2%
Oth Acc
7.1%
Other Causes (<5% Each)
25.2%
Ill-Defined Cardiovascular Disease
Oth CVD
53.5%
IHD
21.9%
Stroke
7.2%
Oth NCD
6.7%
Other Causes (<5% Each)
10.7%
Ill-Defined Injury
Oth Acc
60.8%
RTI
35.8%
Other Causes (<5% Each)
3.4%
Injury of Undetermined Intent
Oth Acc
69.7%
Suicide
18.3%
Homicide
9.7%
Other Causes (<5% Each)
2.3%
Ill-Defined Infectious Disease
HIV & TB
70.1%
Oth Commun
22.0%
Resp Inf
7.9%

ICD 10
Male, Age 60

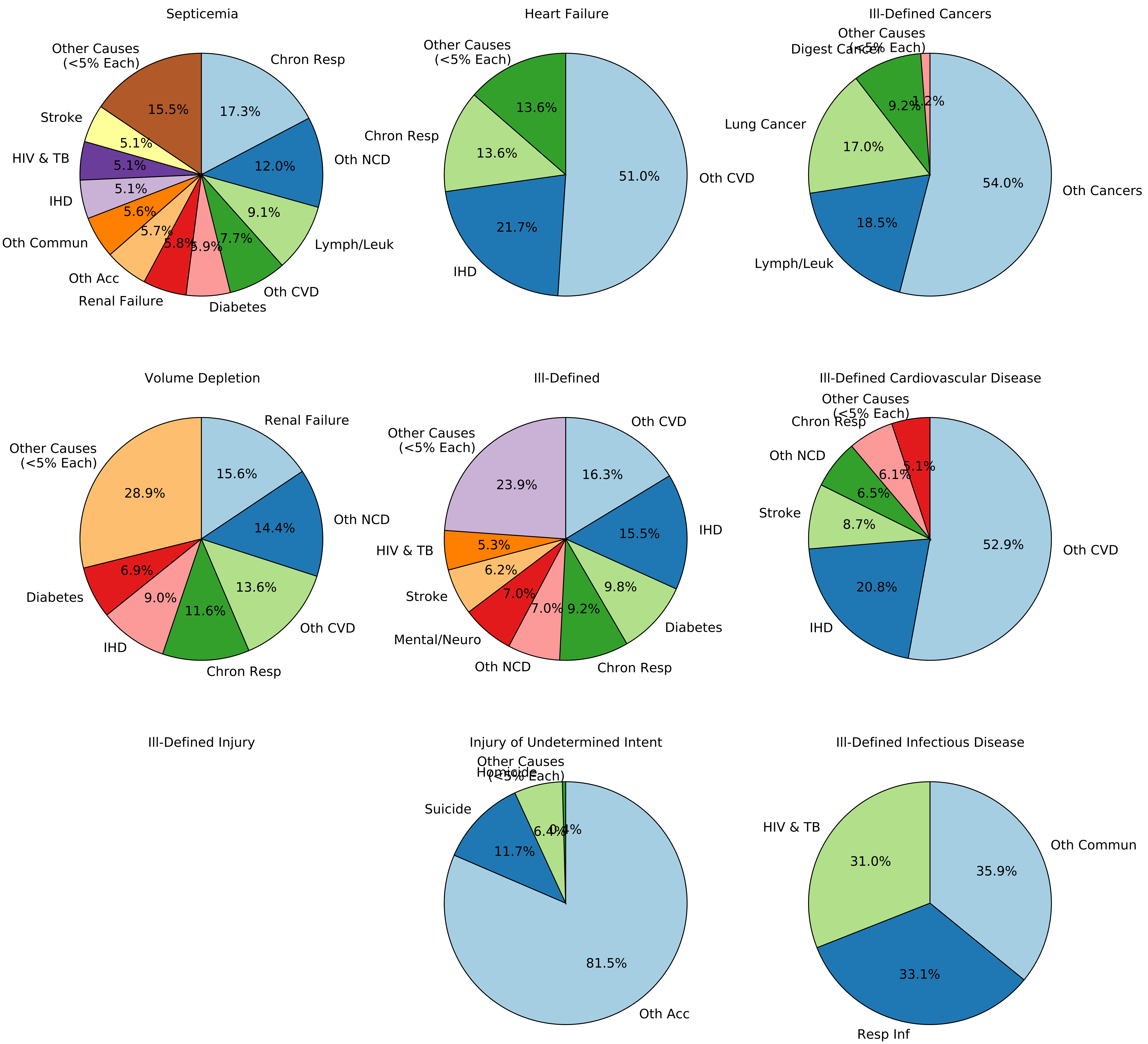

ICD 10
Male, Age 65

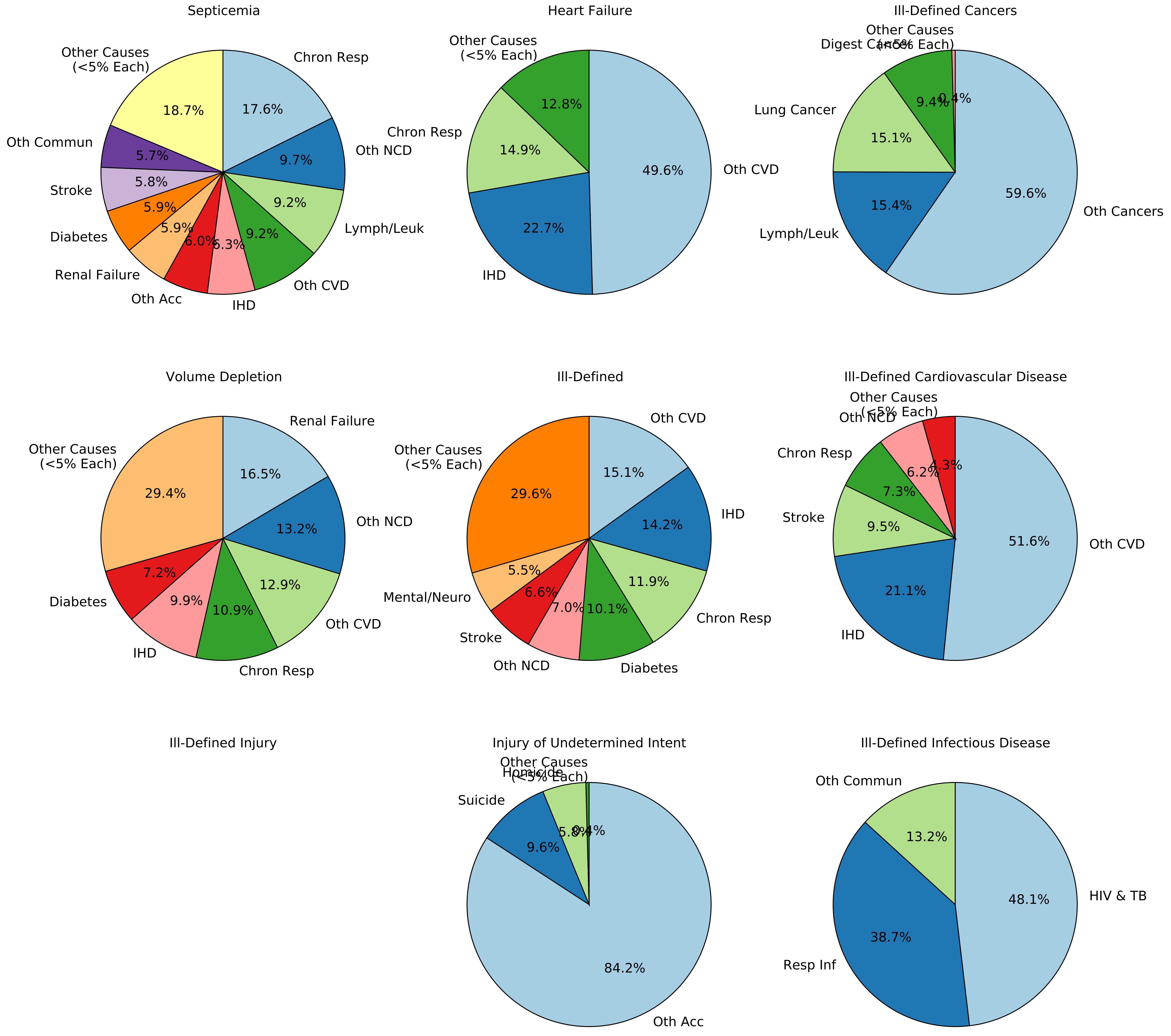

ICD 10
Male, Age 70

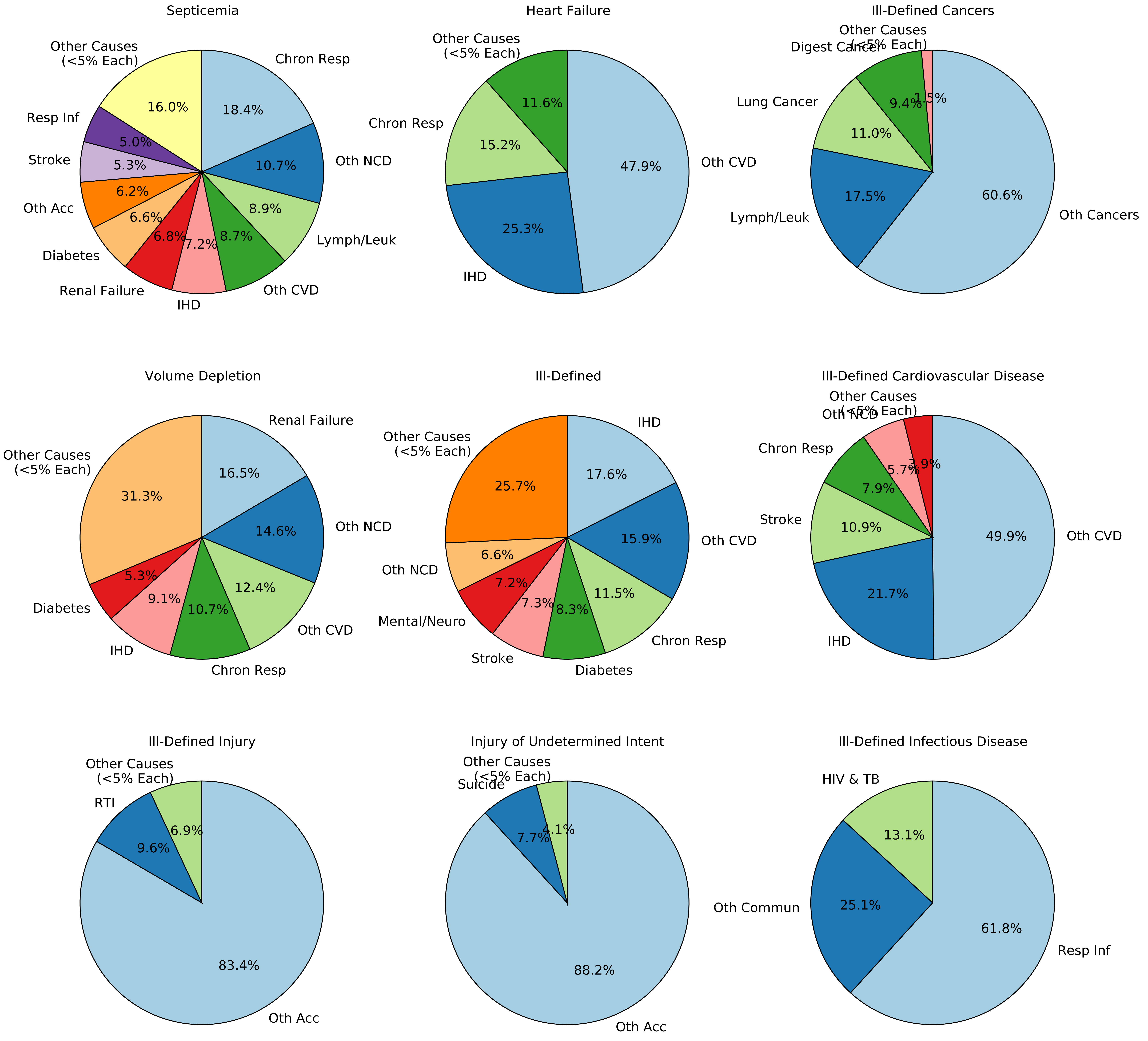

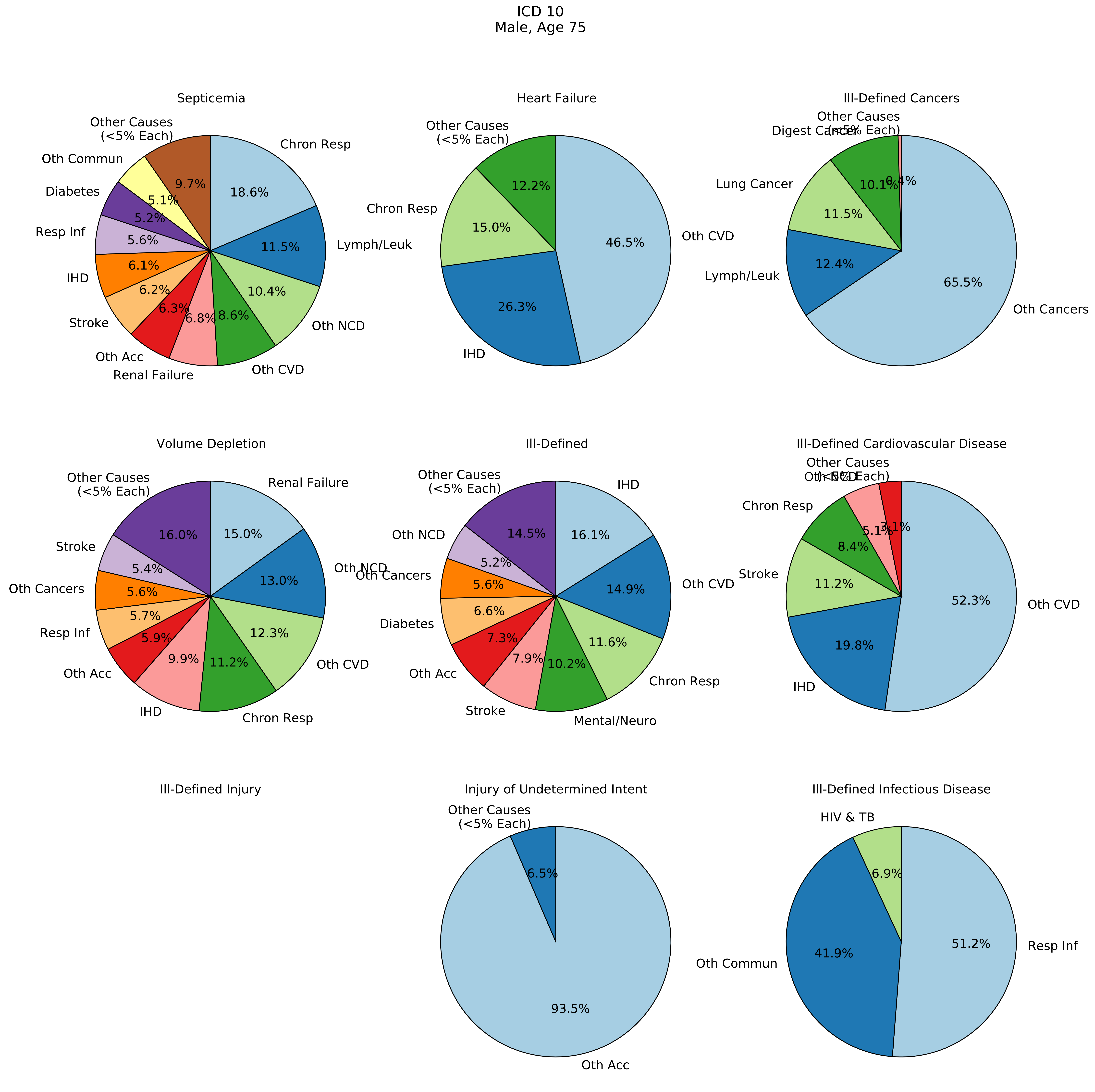
ICD 10
Male, Age 75
Septicemia
Chron Resp 18.6%
Lymph/Leuk 11.5%
Oth NCD 10.4%
Oth CVD 8.6%
Renal Failure 6.8%
Oth Acc 6.3%
Stroke 6.2%
IHD 6.1%
Resp Inf 5.6%
Diabetes 5.2%
Oth Commun 5.1%
Other Causes (<5% Each) 9.7%
Heart Failure
Oth CVD 46.5%
IHD 26.3%
Chron Resp 15.0%
Other Causes (<5% Each) 12.2%
Ill-Defined Cancers
Oth Cancers 65.5%
Lymph/Leuk 12.4%
Lung Cancer 11.5%
Digest Cancer 10.1%
Other Causes (<5% Each) 0.4%
Volume Depletion
Renal Failure 15.0%
Oth NCD 13.0%
Oth CVD 12.3%
Chron Resp 11.2%
IHD 9.9%
Oth Acc 5.9%
Resp Inf 5.7%
Oth Cancers 5.6%
Stroke 5.4%
Other Causes (<5% Each) 16.0%
Ill-Defined
IHD 16.1%
Oth CVD 14.9%
Chron Resp 11.6%
Mental/Neuro 10.2%
Stroke 7.9%
Oth Acc 7.3%
Diabetes 6.6%
Oth Cancers 5.6%
Oth NCD 5.2%
Other Causes (<5% Each) 14.5%
Ill-Defined Cardiovascular Disease
Oth CVD 52.3%
IHD 19.8%
Stroke 11.2%
Chron Resp 8.4%
Oth NCD 5.1%
Other Causes (<5% Each) 3.1%
Ill-Defined Injury
Injury of Undetermined Intent
Oth Acc 93.5%
Other Causes (<5% Each) 6.5%
Ill-Defined Infectious Disease
Resp Inf 51.2%
Oth Commun 41.9%
HIV & TB 6.9%

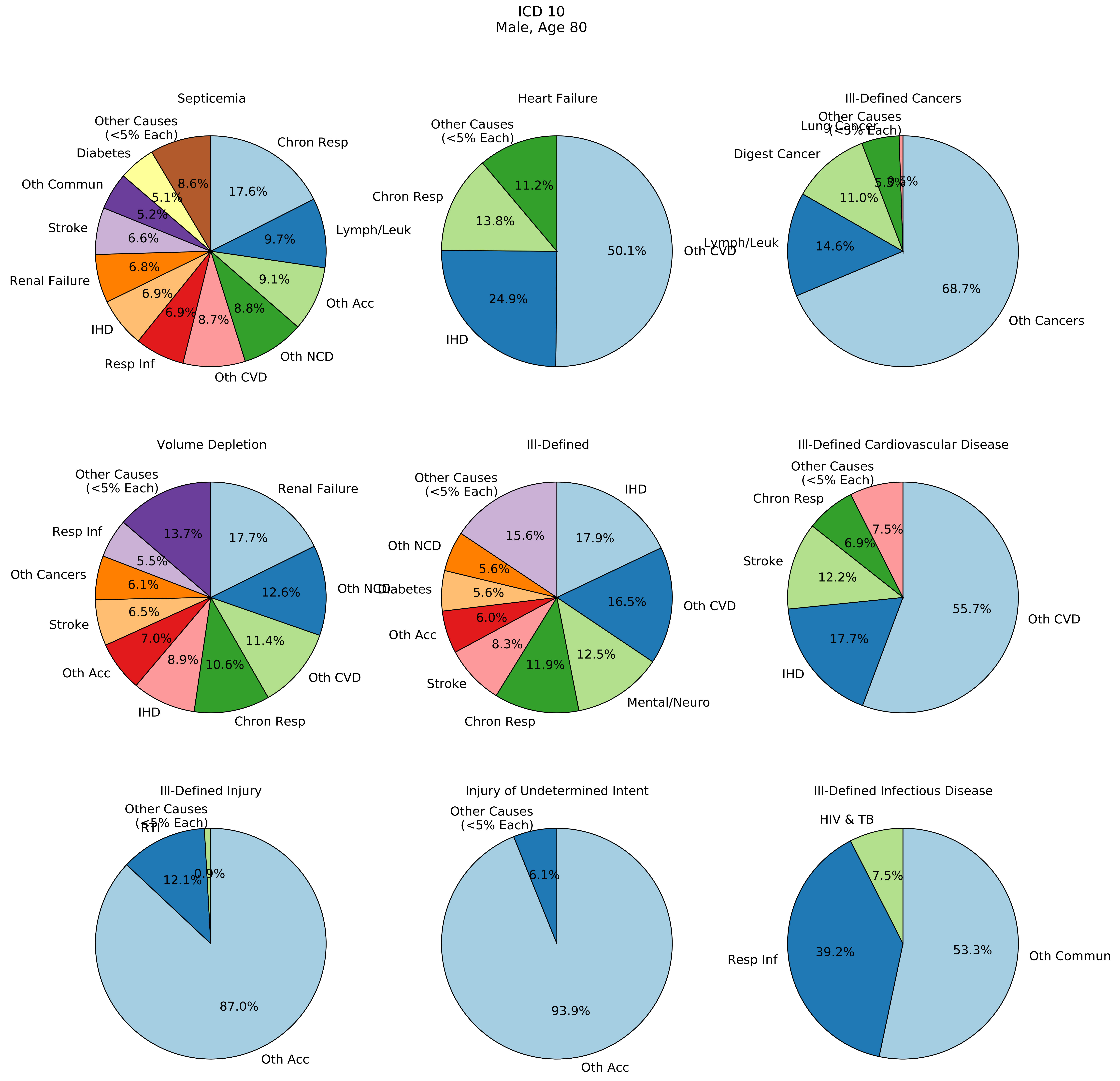
ICD 10
Male, Age 80
Septicemia
Chron Resp 17.6%
Lymph/Leuk 9.7%
Oth Acc 9.1%
Oth NCD 8.8%
Oth CVD 8.7%
Resp Inf 6.9%
IHD 6.9%
Renal Failure 6.8%
Stroke 6.6%
Oth Commun 5.2%
Diabetes 5.1%
Other Causes (<5% Each) 8.6%
Heart Failure
Oth CVD 50.1%
IHD 24.9%
Chron Resp 13.8%
Other Causes (<5% Each) 11.2%
Ill-Defined Cancers
Oth Cancers 68.7%
Lymph/Leuk 14.6%
Digest Cancer 11.0%
Lung Cancer
Other Causes (<5% Each)
Volume Depletion
Renal Failure 17.7%
Oth NCD 12.6%
Oth CVD 11.4%
Chron Resp 10.6%
IHD 8.9%
Oth Acc 7.0%
Stroke 6.5%
Oth Cancers 6.1%
Resp Inf 5.5%
Other Causes (<5% Each) 13.7%
Ill-Defined
IHD 17.9%
Oth CVD 16.5%
Mental/Neuro 12.5%
Chron Resp 11.9%
Stroke 8.3%
Oth Acc 6.0%
Diabetes 5.6%
Oth NCD 5.6%
Other Causes (<5% Each) 15.6%
Ill-Defined Cardiovascular Disease
Oth CVD 55.7%
IHD 17.7%
Stroke 12.2%
Chron Resp 6.9%
Other Causes (<5% Each) 7.5%
Ill-Defined Injury
Oth Acc 87.0%
RTI 12.1%
Other Causes (<5% Each) 0.9%
Injury of Undetermined Intent
Oth Acc 93.9%
Other Causes (<5% Each) 6.1%
Ill-Defined Infectious Disease
Oth Commun 53.3%
Resp Inf 39.2%
HIV & TB 7.5%
